# The circulating dihydrotestosterone/testosterone ratio is increased by gut microbial 5α-reductase activity in females

**DOI:** 10.1016/j.ebiom.2025.105978

**Published:** 2025-10-21

**Authors:** Claes Ohlsson, Lei Li, Karin Horkeby, Lina Lawenius, Hannah Colldén, Klara Sjögren, Gabriel Baldanzi, Gunnar Engström, Johan Ärnlöv, Marju Orho-Melander, Tove Fall, Louise Grahnemo

**Affiliations:** a Department of Internal Medicine and Clinical Nutrition, Institute of Medicine, Sahlgrenska Osteoporosis Centre, Centre for Bone and Arthritis Research at the Sahlgrenska Academy, University of Gothenburg, Gothenburg, Sweden; b Region Västra Götaland, Sahlgrenska University Hospital, Department of Drug Treatment, Gothenburg, Sweden; c Wallenberg Laboratory for Cardiovascular and Metabolic Research, Department of Molecular and Clinical Medicine, Institute of Medicine, Sahlgrenska Academy at University of Gothenburg, Gothenburg, Sweden; d Molecular Epidemiology, Department of Medical Sciences, Uppsala University, Uppsala, Sweden; e Department CIBIO, University of Trento, Trento, Italy; f Department of Clinical Sciences in Malmö, Lund University, Malmö, Sweden; g Department of Neurobiology, Care Sciences and Society/Section of Family Medicine and Primary Care, Karolinska Institutet, Stockholm, Sweden; h School of Health and Social Sciences, Dalarna University, Falun, Sweden; i Science for Life Laboratory, Uppsala University, Uppsala, Sweden

**Keywords:** Dihydrotestosterone to testosterone ratio, Gut microbiota, 5α-reductase, Women

## Abstract

**Background:**

Dihydrotestosterone (DHT), the most potent ligand to the androgen receptor, is synthesised from testosterone (T) by 5α-reductase type 1 and 2. While type 1 is expressed in several non-reproductive tissues in both sexes, men also express high levels of the high-affinity type 2 isoform in reproductive tissues; yet women have a higher circulating DHT to T (DHT/T) ratio than men. We hypothesised that the high DHT/T ratio in women is caused by high gut microbiota (GM) 5α-reductase activity or altered β-glucuronidase-induced androgen reabsorption from the gut.

**Methods:**

We used a large cross-sectional subsample of the Swedish CArdioPulmonary bioImage Study (2897 women and 4338 men, 50–65 years of age) with GM composition and functionality determined by metagenome sequencing and circulating androgens determined by liquid chromatography-tandem mass spectrometry.

**Findings:**

We confirmed that women had higher (+194%) circulating DHT/T ratio than men. The relative abundance of microbial genes for 5α-reductase type 1 (P = 3 × 10^−4^), but not β-glucuronidase, was positively associated with the DHT/T ratio in women. In women, the GM relative abundances of *Odoribacter splanchnicus* and *Parabacteroides distasonis* were positively associated with the relative abundance of microbial genes for 5α-reductase type 1 (P < 2 × 10^−149^) and the circulating DHT/T ratio (*O. splanchnicus* P = 3 × 10^−6^; *P. distasonis* P = 5 × 10^−5^). In mechanistic studies, we observed very high DHT/T ratio in intestinal content of female conventionally-raised but not germ-free mice. In female mice, the DHT/T ratio was 86.9% higher in serum from the portal vein than in inferior vena cava (P = 0.007).

**Interpretation:**

These findings demonstrate that the circulating DHT/T ratio is increased by GM 5α-reductase activity in females. We propose that the GM acts as an endocrine organ influencing the androgenic status in females.

**Funding:**

See Acknowledgements.

**Research in context**

**Evidence before this study**

Androgens, mainly testosterone (T) and dihydrotestosterone (DHT), have implications for the well-being of not only men but also for women, exemplified in women by the androgen excess observed in polycystic ovary syndrome. Although men have higher androgen levels than women, a recent study showed that the DHT to T (DHT/T) ratio is higher in women than in men. However, the reason for the higher ratio in women compared with men was not investigated.

**Added value of this study**

Using a large population-based study (2897 women and 4338 men) with sensitive measures of androgens, we confirmed the finding from the recent small-scale study showing that premenopausal women have higher circulating DHT/T ratio compared with men, and we also showed that the DHT/T ratio was higher in postmenopausal women compared with men. Using metagenome sequencing, we further extended the previous study by showing that the relative abundance of microbial genes for 5α-reductase type 1, an enzyme converting testosterone to dihydrotestosterone, was positively associated with the DHT/T ratio in women. The presence of gut microbial conversion of T to DHT was supported by our experimental studies in mice.

**Implications of all the available evidence**

Women have a higher ratio of DHT/T; a finding that is explained at least partly by a higher gut microbial conversion of T to DHT via an increased 5α-reductase activity in females. We propose that the gut microbiota acts as an endocrine organ influencing the androgenic status in females. Further studies are needed to determine the importance of gut microbial conversion of T to DHT in men with low endogenous androgen production and to determine the role for DHT/T ratio for health.

## Introduction

The main androgens testosterone (T) and dihydrotestosterone (DHT) are found in both men and women. In men, androgens are important for a variety of physiological processes including sexual development, fertility, and muscle mass.^1^^,^^2^ Although less obvious than in men, androgens also exert important physiological and pathophysiological effects in women. First, T is important as a precursor for oestrogen synthesis.^3^ In addition, women with androgen deficiency display reduced well-being^4^ and T treatment increases the sexual drive and satisfaction in postmenopausal women.^5^ Furthermore, androgen excess in females can be harmful, as demonstrated by the close connection between androgen excess and polycystic ovary syndrome (PCOS).^6^ As in men, the conversion of T into DHT is of importance in females, exemplified by delayed menarche, reduced body hair, and no history of acne in women with genetically reduced DHT levels.^7^

DHT is the most potent ligand to the androgen receptor. It is synthesised from T by 5α-reductase type 1 or type 2. The conversion of T into the more potent androgen DHT could be regarded as an amplification step in androgen signalling. In addition, this conversion results in a specific androgen receptor targeted signalling as DHT cannot be converted to oestrogens, excluding oestrogen receptor signalling activity. 5α-reductase type 2, with high affinity for T, is highly expressed in male reproductive organs including the prostate, while 5α-reductase type 1 is expressed in several non-reproductive tissues (mainly skin, liver, oesophagus) in both women and men.^8^^,^^9^

The circulating levels of androgens are not only affected by their production, but also by their metabolism. The metabolism of androgens is complex, with the liver inactivating androgens and destining them for excretion following enzymatic conjugation to, for example, glucuronic acid or sulphate.^3^ Although the regulation of androgen metabolism is not fully understood, the gut microbiota (GM) might be involved.^10^, ^11^, ^12^, ^13^

Despite the high expression of 5α-reductase type 2 in male reproductive organs, women have been reported to have higher circulating DHT/T ratio than men.^14^ In the present study, we first confirmed the higher circulating DHT/T ratio in women compared with men, using a large subsample of the Swedish CArdioPulmonary bioImage Study (SCAPIS; n = 2897 women and 4338 men, in total 7235). We hypothesised that the high circulating DHT/T ratio in women is caused by elevated bacterial 5α-reductase activity or altered β-glucuronidase-induced androgen reabsorption from the gut. Using a translational approach, we herein demonstrate that the circulating DHT/T ratio is increased by GM 5α-reductase activity in females.

## Methods

### Study participants

SCAPIS is a prospective population-based study of 30,154 men and women who were 50–65 years old and living in six municipality areas in Sweden at recruitment.^15^ Participants were randomly selected from the population register and recruited via mail between 2013 and 2018. The participation rate was 50%. Participants underwent extensive imaging and anthropometric measurements, contributed with blood and faecal samples, and responded to detailed questionnaires regarding their health, medication use, menstruations, etc.^15^

**Fig. 1:**
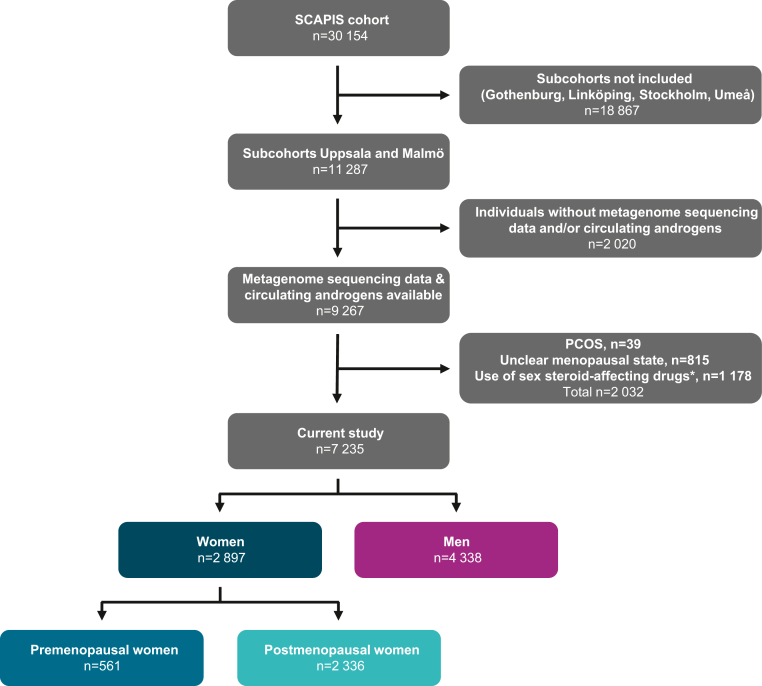
**Flow chart.** Numbers of excluded and included participants. ∗See Table S1 for details.

The present study was based on participants from Uppsala and Malmö with high-quality metagenome sequencing data (n = 9816) that also had data on circulating DHT and T levels (n = 9267, Fig. 1). No sample size calculation was performed; to optimise power, we used all available participants that were not subjected to exclusion. We excluded participants with PCOS, unclear menopausal state, use of medications that interfere with sex steroid levels (including drugs with ATC code G03, to which hormone replacement therapy belongs to, dispensed within 12 months before baseline), and participants with probable unreported use of sex steroid-affecting drugs, indicated by unreasonably high sex steroid levels for their sex and menopausal state (Table S1). In total, 2032 participants were excluded, leaving 7235 participants for analysis (2897 women [561 premenopausal and 2336 postmenopausal] and 4338 men, Fig. 1).

All study participants provided written informed consent at their first site visit. The present study adheres to the Declaration of Helsinki and was approved by the regional ethics committees or the Swedish Ethics Review Authority (Etikprövningsmyndigheten Dnr 2010-228-31M, Dnr 2018-315, Dnr 2023-06572-02). The participants did not receive any compensation for their participation.

### Mouse experiments

The mouse experiments were performed in accordance with the relevant guidelines and regulations and reported in accordance with ARRIVE guidelines. The experimental unit is single mice. Mice were supervised several times a week and terminated if their general condition was deteriorated; if in doubt, a veterinarian was consulted. As determined a priori, mice that were unhealthy were excluded from the study before statistical analyses were performed (four mice from experimental study 1 was removed because of complications with surgery or fighting, but none in the other studies), with no further exclusions of data. Potential confounders were minimised by performing treatments and terminations of mice in a mixed order of cages. No sample size calculation was performed; to optimise power, we used all available mice (from previously performed experiments and current breeding). For the previously performed experiments, the sample size was based on earlier experience of studies on the effect of changes in the microbiome on parameters related to bone health.

#### Experimental study 1—Relative abundance of microbial genes for 5α-reductase type 1 and β-glucuronidase in caecal contents of mice

C57BL/6J female mice (Charles River, Germany) were housed in a standard animal facility under controlled temperature (22 °C) and photoperiod (12 h light/dark cycle), with free access to fresh water and pellet diet with 12% kcal from fat (Teklad diet 2016, Envigo, Indianapolis, Indiana, USA). The mice were randomised into groups (n = 12/group and 4 mice/cage) and acclimatized for two weeks to the animal facility. Originally, the experiment was designed to study probiotic treatment, and those data have previously been published.^16^ Here, we present relative abundance data for microbial genes determined by metagenome sequencing, only from the control group (sham operated and treated with vehicle) of the original study.

At 10 weeks of age, the mice used in the current study were sham operated under anaesthesia with isoflurane (Baxter Medical AB), and Meloxicam (Metacam, Boehringer Ingelheim Animal Health Nordics A/S) was given as postoperative analgesic. Following surgery, the mice had free access to water supplemented with maltodextrin and a pellet diet with 10% kcal from fat (D12450J, Research Diets, New Brunswick, NJ, US). A few mice were removed during the study due to complications with the surgery or fighting, ending up with n = 8 mice in the group used in this study. Twelve weeks later, mice were fasted for 3 h, anaesthetised with Ketador (VetViva Richter GmbH, Wels, Austria) and Dexdomitor (Orion Pharma AB, Animal Health, Sollentuna, Sweden), bled from the axillary vein, and killed by cervical dislocation. Caecal contents were collected and snap frozen in liquid nitrogen. Group allocation was unknown at termination, except for the one person removing the mice from the cages. This experiment was approved by the regional animal ethics committee in Gothenburg (ethics number: 4593/22).

#### Experimental study 2—DHT/T ratio in germ-free mice

DHT/T ratios in different tissues were tested in conventionally raised murine pathogen-free and germ-free male and female C57BL/6 mice (n = 10 per group). Other data from these mice have previously been published.^10^ Briefly, the mice were housed in Taconic facilities (Germantown, NY) and fed autoclaved NIH-31M diet. Sterility of germ-free mice was routinely confirmed by aerobic and anaerobic faecal cultures and 16S bacterial RNA screening via PCR of faecal samples. At 8 weeks of age, the mice were anaesthetised, bled from the axillary vein, and killed by cervical dislocation. Tissues and intestinal contents were collected, weighed, and snap frozen in liquid nitrogen.

#### Experimental study 3—vena cava and porta androgen levels in female mice

Androgen levels in serum from vena cava and vena porta were determined in female C57BL/6 mice (n = 12) bred at the animal facility at University of Gothenburg. The mice were housed in a standard animal facility under controlled temperature (22 °C) and photoperiod (12 h light/dark cycle), with free access to fresh water and pellet diet (Teklad diet 2016, Envigo). At 5–6 months of age, the mice were anaesthetised with Ketador (VetViva Richter GmbH) and Dexdomitor (Orion Pharma AB). Blood was collected from the inferior vena cava and vena porta as previously described,^17^ except that we used a micro serrefine clamp (18055-03, AgnTho's AB, Lidingö, Sweden) instead of a suture to increase the blood volume in the vena porta. Blood was allowed to clot for at least 30 min, centrifuged for 10 min, and stored in −80 °C. This experiment was approved by the regional animal ethics committee in Gothenburg (ethics number: 4593/22).

### Metagenome sequencing

For SCAPIS participants, the GM sample collection, DNA extraction, and metagenome sequencing has previously been described.^18^ Briefly, faecal samples were collected at home after the first visit and kept in the freezer at home until they were returned at the second visit (except for a minority of samples that were returned later). Following shipment to Clinical Microbiomics A/S (Copenhagen, Denmark), samples were subjected to DNA extraction, metagenome sequencing using an Illumina Novaseq 6000 system (Illumina, USA), bioinformatic processing, gut microbiome profiling using the Clinical Microbiomics Human Microbiome Profiler (CHAMP™) pipeline which utilises the GTDB r214 for taxonomic annotation of prokaryotes,^19^ and functional annotation and profiling using the EggNOG orthologous groups database (v. 5.0)^20^ and Kyoto Encyclopedia of Genes and Genomes (KEGG) orthology database as the reference (http://www.kegg.jp/kegg/).^21^^,^^22^

For mice in experimental study 1, the GM sample collection, DNA extraction, metagenome sequencing, and bioinformatic processing has previously been described.^16^ Briefly, total DNA was extracted from caecal content using the QIAamp Fast DNA Stool kit (Qiagen, Hilden, Germany), and samples were then sent to Novogene (UK) Company Limited for library construction, metagenome sequencing using an Illumina NovaSeq x Plus platform, bioinformatic processing, and annotation of functional orthologs using the KEGG orthology database as the reference.^16^^,^^21^^,^^22^

### Androgen analyses

For measurements of androgens, commercial ELISA kits are not as accurate and specific as mass spectrometry methods, especially at the low levels of androgens anticipated in women and mice.^23^^,^^24^ We, therefore, used our own developed^25^^,^^26^ efficient mass spectrometry methods that are highly specific and sensitive. Details on the condition for the liquid-phase mass spectrometry and gas-phase mass spectrometry methods that are previously developed by us are given in our previously published articles describing the establishment of these methods^25^^,^^26^ and details on the pretreatment of different tissue samples before being analysed using these techniques are described in our previous studies evaluating multiple different animal tissue samples.^10^^,^^25^

For SCAPIS participants, serum levels of T and DHT were analysed using liquid chromatography tandem mass spectrometry (LC-MS/MS; lower limit of quantification [LLOQ], T, 5 pg/mL, DHT, 13 pg/mL).^26^ The intraassay coefficient of variation (CV) was 3.0% for DHT at low quality control (QC, 45 pg/mL) and 3.6% at high QC (526 pg/mL) and 1.1% for T at low QC (228 pg/mL) and 1.9% at high QC (4993 pg/mL), and the interassay CV was 4.7% for DHT at low QC (78 pg/mL) and 4.1% at high QC (526 pg/mL) and 2.2% for T at low QC (228 pg/mL) and 2.4% at high QC (4993 pg/mL). Androgen levels below LLOQ (n = 0 for testosterone and n = 75 for DHT) were set to the LLOQ in the statistical analyses.

For mice in experimental study 2 and 3, DHT and T levels were analysed in serum, tissues, and intestinal contents using sensitive gas chromatography tandem mass spectrometry (GC-MS/MS).^10^^,^^25^ In germ-free and conventionally-raised mice (experimental study 2), the functional LLOQ was 8 pg/mL for DHT and 20 pg/mL for T in tissues and 20 pg/mL for DHT and 40 pg/mL for T in intestinal contents, and in serum from vena cava and vena porta (experimental study 3), the functional LLOQ was 0.64 pg/mL for DHT and 2.4 pg/mL for T. Undetectable levels of androgens were set to LLOQ.

### Questionnaires

Participants responded to questionnaires regarding their lifestyle and health. Participants were classified as having PCOS if they responded yes to whether they were ever diagnosed with PCOS. For menopausal state, women were classified as premenopausal if they responded that they had their period last year, postmenopausal if they stated that they did not have their period last year due to menopause, or unclear menopausal state (and thus excluded) if they did not responded to why they did not have their period last year or whether it was due to gynaecological surgery, medication, pregnancy, or exercise. Use of endocrine therapy (ATC codes L02) and testosterone-5α-reductase inhibitors (ATC codes G04CB) were identified manually from a free text question regarding use of prescription medication. Participants were classified as born in Sweden if they responded yes to being born in Sweden and as born abroad if they responded no. Dietary intake values (energy intake in kcal/day and intake of fat, fibres, protein, and carbohydrates in grams/day) were calculated from a food frequency questionnaire (MiniMealQ).^27^

### Register data

Use of sex hormones and modulators of the genital system (ATC code G03, including hormone replacement therapy) within 12 months before baseline and antibiotics (ATC codes J01) within 3 months before baseline was defined as dispensed prescriptions retrieved from the Swedish Prescribed Drug Register. Gender was determined using register data from the population register.

### Statistics

#### Software

Statistical analyses were performed using R v4.3.1 (https://cran.r-project.org/), while graphs were created using GraphPad Prism v10.3.1.

#### Handling of missing data

We did not impute missing values as none of the included participants missed information on variables included in the main reported statistical analyses.

#### Variable transformations

Continuous outcomes and exposure variables such as DHT, T, DHT/T ratio, and relative abundance of species and microbial genes (KEGG orthologs [KO] K12343 and K01195) were inverse rank transformed, unless otherwise specified, to ensure normally distributed data.

#### Statistical analyses

For analyses in the SCAPIS cohort, we assessed associations between circulating androgens and GM metrices (species and genes) using linear regressions adjusted for age, extraction plate, and antibiotic use. For combined analyses of pre- and postmenopausal women, we further adjusted for menopausal status. We determined differences between groups (women/men, premenopausal women/postmenopausal women, or premenopausal women/postmenopausal women/men) using ANCOVA adjusted for age and study site (not involving metagenome sequencing data) or age, extraction plate, and antibiotic use (involving metagenome sequencing data). When comparing three groups, we used the Bonferroni post-hoc test to determine differences between groups. We used Pearson's correlation to determine the correlations between GM species.

For analyses in mice, we determined differences between groups (conventionally-raised vs. germ-free mice) using Student's t-test and differences between blood sampling site (vena cava vs. vena porta) using paired samples t-test.

All tests were 2-sided. In general, we considered P < 0.05 to be statistically significant. However, when testing associations between species and microbial genes encoding for 5α-reductase type 1, we tested 470 species with a prevalence higher than 30% in women and men separately. To account for this multiple testing, we considered associations to be statistically significant if P values were below the conservative Bonferroni correction threshold (P < 1.063 × 10^−4^ [0.05/470]).

### Role of funders

The funding sources had no role in the study design, data collection, data analysis, data interpretation, writing of the paper, or in the decision to submit the paper for publication.

## Results

### The relative abundance of microbial genes for 5α-reductase type 1 is positively associated with the circulating DHT/T ratio in women

**Fig. 2:**
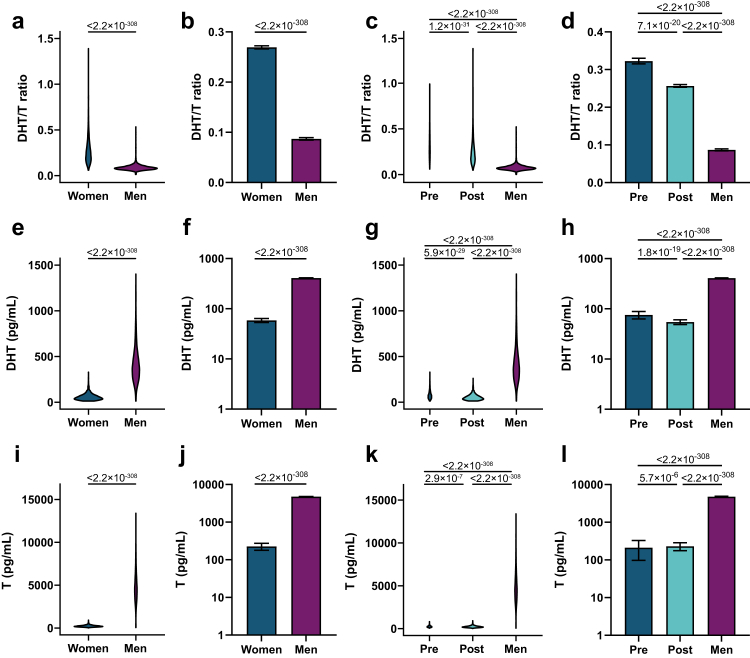
**The circulating DHT/T ratio is higher in women than in men.** (a–d) Circulating DHT/T ratio in (a–b) women and men and in (c–d) premenopausal women, postmenopausal women, and men on a linear and logged scale. (e–h) Circulating DHT levels in (e–f) women and men and in (g–h) premenopausal women, postmenopausal women, and men on a linear and logged scale. (i–l) Circulating T levels in (i–j) women and men and in (k–l) premenopausal women, postmenopausal women, and men. (a–l) ANCOVA on inverse rank transformed data adjusted for age and site, with Bonferroni as the post hoc test when comparing 3 groups. Data are presented as (a, c, e, g, i, k) violin plots and (b, d, f, h, j, l) untransformed estimated marginal means ±95% confidence intervals (CI). n = 2897 women, 561 premenopausal women, 2336 postmenopausal women, 4338 men.

To determine the impact of the GM on the circulating DHT/T ratio, we used a large subsample of the SCAPIS cohort (7235 individuals; 2897 women, 57.6 ± 4.3 years of age mean ± standard deviation (SD); 4338 men, 57.6 ± 4.4 years of age; Fig. 1, Table 1) with metagenome sequencing data and circulating levels of T and DHT. Among the women, 561 were premenopausal (52.7 ± 2.0 years of age) and 2336 were postmenopausal (58.8 ± 3.8 years of age; Fig. 1, Table 1). The circulating DHT/T ratio was substantially higher in women than in men (+194%, Fig. 2a and b), with slightly higher ratio in pre-compared with postmenopausal women (Fig. 2c and d). As expected, the circulating androgen levels were higher in men than in women (Fig. 2e–l).

**Table 1:** Study characteristics.

Variables	All	Men	Women	Premenopausal women	Postmenopausal women
	(n = 7235)	(n = 4338)	(n = 2897)	(n = 561)	(n = 2336)
Women, n (%)	2897 (40.0)	0 (0)	2897 (100)	561 (100)	2336 (100)
Age (years), mean (SD)	57.6 (4.3)	57.6 (4.4)	57.6 (4.3)	52.7 (2)	58.8 (3.8)
Antibiotic treatment, n (%)	393 (5.4)	233 (5.4)	160 (5.5)	27 (4.8)	133 (5.7)
Height (cm), median (IQR)	174 (167–181)	179 (175–184)	166 (161–170)	167 (162–172)	165 (161–169)
Weight (kg), median (IQR)	81.8 (71.2–92.2)	87.0 (79.5–97.0)	71.0 (63.3–80.0)	70.4 (64.0–81.0)	71.0 (63.0–80.0)
BMI (kg/m^2^), median (IQR)	26.7 (24.3–29.7)	27.1 (24.9–29.8)	25.9 (23.3–29.3)	25.3 (23.1–29.0)	26 (23.4–29.3)
DHT (pg/ml), median (IQR)	243.7 (60.3–417.0)	380.4 (279.8–501.6)	50.9 (33.3–74.5)	70.2 (47.7–99.0)	47.1 (31.4–68.7)
T (pg/ml), median (IQR)	3239.9 (238.6–4891.9)	4584.3 (3607.4–5721.7)	206.8 (155.2–274.3)	202.7 (151.3–269.2)	232 (173.7–288.9)
DHT/T ratio, median (IQR)	0.10 (0.08–1.39)	0.08 (0.07–0.53)	0.24 (0.17–1.39)	0.23 (0.17–1.39)	0.30 (0.22–1.00)

SD, standard deviation, IQR, interquartile range; BMI, body mass index; DHT, dihydrotestosterone.

As it has been reported that the gut microbiota regulates androgen metabolism in mice, we, therefore, hypothesised that the higher DHT/T ratio in circulation in women is caused by elevated GM 5α-reductase activity or altered β-glucuronidase-induced androgen reabsorption from the gut.

**Fig. 3:**
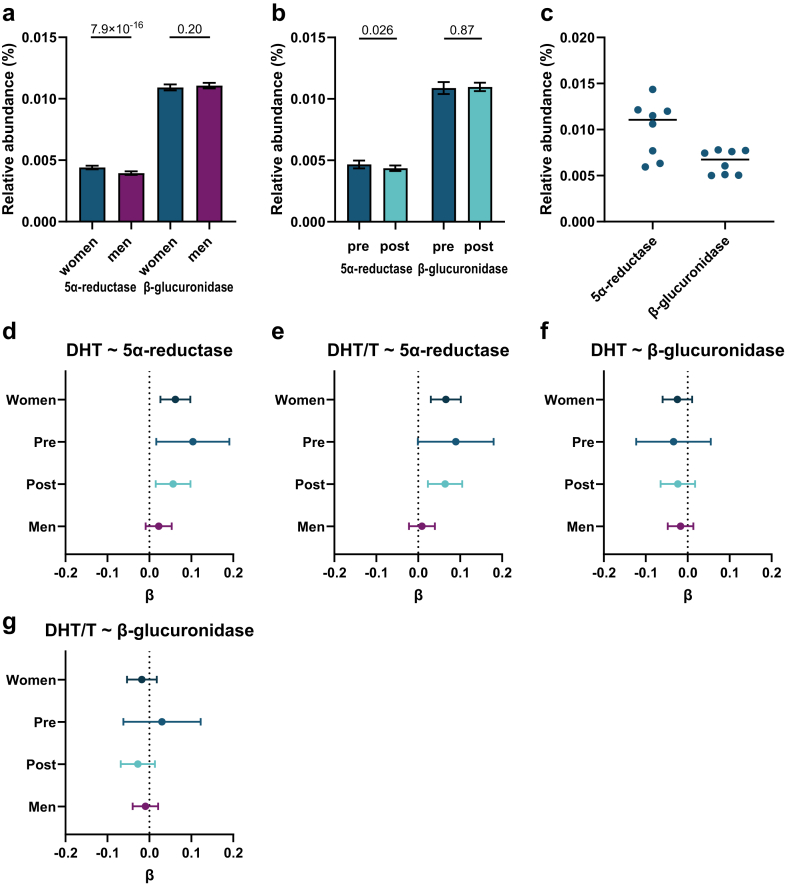
**The relative abundance of microbial genes for 5α-reductase type 1 is positively associated with the circulating DHT/T ratio in women.** (a–b) Relative abundance of microbial genes for 5α-reductase type 1 and β-glucuronidase in (a) women and men, (b) premenopausal women and postmenopausal women, and in (c) female mice. (d–g) Associations between relative abundance of microbial genes for 5α-reductase type 1 and circulating (d) DHT levels and (e) DHT/T ratio and relative abundance of microbial genes for β-glucuronidase and circulating (f) DHT levels and (g) DHT/T ratios. Data were analysed using (a–b) ANCOVA on inverse rank transformed data adjusted for (a–b) age, extraction plate, and antibiotic use, and (d–g) linear regressions of inverse rank transformed data (outcome and exposure) adjusted for age, extraction plate, antibiotic use (and menopausal state for women). Data are presented as (a–b) untransformed estimated marginal means ± 95% confidence intervals (CI), (c) a scatter plot of raw data with the bar showing the median, and (d–g) β and 95% CI with β expressed as standard deviation (SD) change in circulating sex steroid levels per SD change in relative abundance of microbial genes. n = 2897 women, 561 premenopausal women, 2336 postmenopausal women, 4338 men, and 8 mice.

Metagenome sequencing, followed by functional analyses using KEGG orthologs (KO), showed that feces from both humans and mice contained microbial genes encoding for both 5α-reductase type 1 (K12343, present in 99.8% of the human participants and in all investigated mice) and β-glucuronidase (K01195, present in all human participants and in all investigated mice; Fig. 3a–c). The relative abundance of microbial genes for 5α-reductase type 1*,* but not β-glucuronidase*,* was positively associated with circulating DHT and DHT/T ratio in women but not in men (DHT in women: β 0.062, 95% confidence interval (CI) 0.026–0.098, P = 0.00066, DHT/T in women: β 0.066, 95% CI 0.03–0.10, P = 0.00032; Fig. 2d–g). Similarly, the relative abundance of microbial genes for 5α-reductase type 1 activity was positively associated with the circulating DHT/T ratio in both pre- and postmenopausal women (Fig. 2d and e). The relative abundance of microbial genes for 5α-reductase type 1 was higher in women compared with men (Fig. 3a) and slightly higher in pre-compared with postmenopausal women (Fig. 3d).

These descriptive findings demonstrate that the relative abundance of microbial genes for 5α-reductase type 1 is positively associated with the relatively high circulating DHT/T ratio in women.

### *Odoribacter splanchnicus* and *Parabacteroides distasonis* are positively associated with the GM 5α-reductase type 1 and the circulating DHT/T ratio in women

**Fig. 4:**
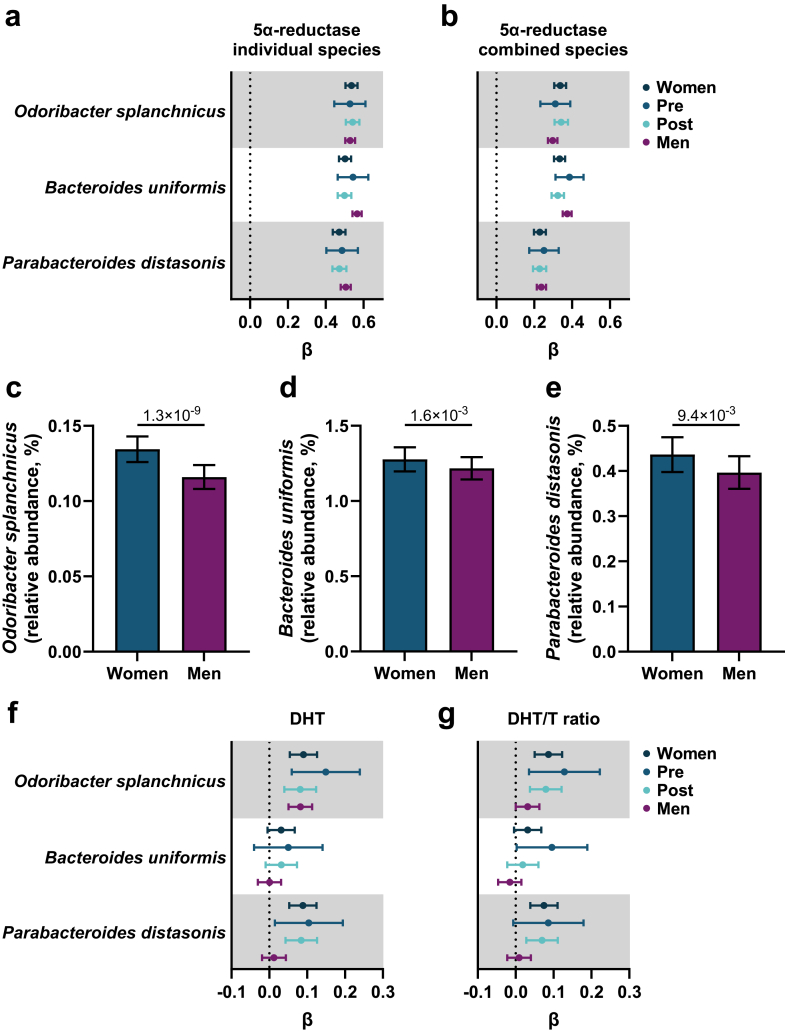
***Odoribacter splanchnicus* and *Parabacteroides distasonis* are positively associated with the GM 5α-reductase type 1 and the circulating DHT/T ratio in women.** (a–b) Associations between *Odoribacter splanchnicus*, *Bacteroides uniformis*, and *Parabacteroides distasonis* and relative abundance of microbial genes for 5α-reductase type 1 in models including (a) individual species and (b) all three species. (c–e) Relative abundance of (c) *Odoribacter splanchnicus*, (d) *Bacteroides uniformis*, and (e) *Parabacteroides distasonis* in women and men. (f–g) Associations between *Odoribacter splanchnicus*, *Bacteroides uniformis*, and *Parabacteroides distasonis* and circulating (f) DHT and (g) DHT/T ratio. Data are analysed using (a–b, f–g) linear regression models of inverse rank transformed data (outcome and exposure) adjusted for age, extraction plate, antibiotic use (and menopausal state for women) and (c–e) ANCOVA on inverse rank transformed data adjusted for age, extraction plate, and antibiotic use. Showing similar results as in c–e, sensitivity analyses further adjusting for diet (energy intake in kcal/day and intake of fat, fibres, protein, and carbohydrates in grams/day) and country of birth (born in Sweden; yes/no) are shown in Table S2). Data are presented as (a–b, f–g) β and 95% CI, with β expressed as standard deviation (SD) change in relative abundance of the outcome per SD change in the exposure, and (c–e) untransformed estimated marginal means ± 95% confidence intervals. n = 2897 women, 561 premenopausal women, 2336 postmenopausal women, and 4338 men.

To identify the major bacterial species contributing to the microbial genes for 5α-reductase type 1, we next determined the association between GM species (species with a presence >30%, n = 470) and microbial genes for 5α-reductase type 1 activity (Fig. 4; Table S3). Possible sources of microbial genes for 5α-reductase type 1 are *O. splanchnicus*, *Bacteroides uniformis*, and *P. distasonis* as these species were most strongly associated with the relative abundance of microbial genes for 5α-reductase type 1 (Fig. 4a; Table S3). These three species correlated modestly with each other (Table S4). To determine their independent associations with GM 5α-reductase type 1, we included all three species in the same linear regression model. Although the effect sizes were slightly attenuated in the combined model, all three species remained independently associated with the relative abundance of microbial genes for 5α-reductase type 1 (Fig. 4b). The relative abundances of these three species were higher in women than in men (Fig. 4c–e), while no difference between pre- and postmenopausal women was observed (Fig. S2). Higher relative abundances of *O. splanchnicus* and *P. distasonis* were associated with higher circulating DHT and DHT/T ratio in women (Fig. 4f and g). In a combined model, including both *O. splanchnicus* and *P. distasonis* as exposures, both species were independently associated with the circulating DHT/T ratio in women (*O. splanchnicus* β 0.67, 95% CI 0.0027–0.11, P = 0.0010; *P. distasonis* β 0.047, 95% CI 0.007–0.087, P = 0.022). Thus, the relative abundances of *O. splanchnicus* and *P. distasonis* are positively associated with both the relative abundance of microbial genes for 5α-reductase type 1 and the circulating DHT/T ratio in women. These findings suggest that GM 5α-reductase type 1 activity, derived from *O. splanchnicus* and *P. distasonis,* increases the circulating DHT/T ratio in women, but functional studies are required.

In the present association study in the SCAPIS cohort, we adjusted the analyses for recent antibiotic use, but similar strengths of the main associations were also observed in sensitivity analyses excluding subjects on recent antibiotic treatment (Table S5).

### The GM increases the DHT/T ratio in intestinal content and in the circulation in female mice

**Fig. 5:**
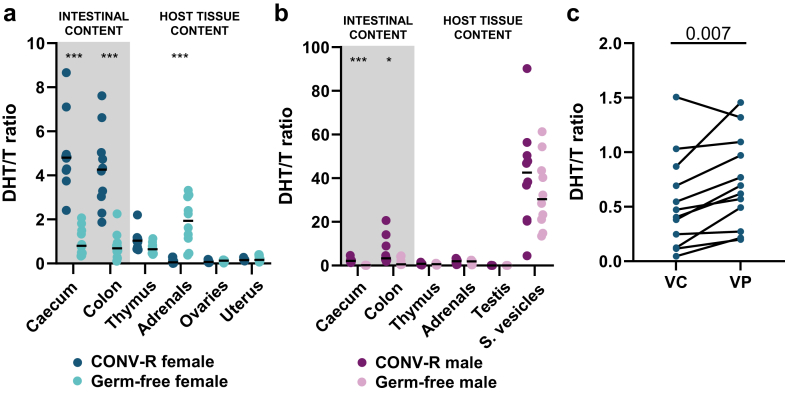
**The GM increases the DHT/T ratio in intestinal content and in the circulation of female mice.** (a–b) DHT/T ratio in intestinal content and host tissues of (a) female and (b) male conventionally-raised mice (CONV-R) and germ-free (GF) mice. (c) The DHT/T ratio in serum from inferior vena cava (VC) and vena porta (VP) in female mice. Data were analysed using (a–b) Student's t-test of inverse rank data and (c) paired samples t-test of untransformed data. Data are presented as scatter plots of untransformed data with (a–b) the bar showing the median and (c) the lines indicating samples from individual mice. Absolute levels of DHT and T are for the VC and VP experiment shown in Fig. S2. Absolute levels of DHT and T are for the CONV-R and GF mice given in a previous publication,^10^ demonstrating that the lower DHT/T ratio in GF mice compared with CONV-R mice mainly was driven by lower DHT in intestinal content of GF mice (colonic content: 85% lower DHT and 8% higher T in GF compared with CONV-R mice^10^). (a–b) n = 10 per group and (c) n = 12.

To determine the role of the GM 5α-reductase activity for the DHT/T ratio in intestinal content and in the circulation, we next performed mechanistic studies in mice. We first determined the role of GM for the DHT/T ratio in intestinal content. In conventionally-raised female mice, the DHT/T ratio in the intestinal content was substantially higher than in any investigated host tissue, while male mice had the highest DHT/T ratio in seminal vesicles, a male reproductive organ with a known high endogenous 5α-reductase type 2 activity (Fig. 5a and b). In conventionally-raised female mice, a DHT/T ratio above 1 (=more DHT than T) was only observed in intestinal contents, while male mice displayed a DHT/T ratio far above 1 also in seminal vesicles, a male reproductive tissue. The DHT/T ratio in intestinal content was substantially reduced in germ-free mice compared with conventionally-raised mice (Fig. 5a and b), demonstrating that GM is a major contributor to the functional 5α-reductase activity in intestinal content. Unexpectedly, we observed a compensatory increased DHT/T ratio in the adrenals of female germ-free mice (Fig. 5a).

Finally, to determine the importance of the GM for the circulating DHT/T ratio in female mice, we sampled the blood from the *inferior vena cava*, carrying blood from the hindlimbs and the abdomen to the heart (reflecting systemic circulation), and the *vena porta*, carrying blood from the intestines to the liver. We found that the DHT/T ratio in serum from the portal vein was 86.9 ± 34.3% (mean ± SD) higher than in serum from the systemic circulation (P = 0.007, Fig. 5c; Fig. S2).

## Discussion

We, herein, hypothesised that the relatively high circulating DHT/T ratio in women is caused by elevated GM 5α-reductase activity or altered β-glucuronidase-induced androgen reabsorption from the gut. Using a large subsample of the SCAPIS cohort with GM determined by metagenome sequencing and circulating T and DHT determined by liquid chromatography-tandem mass spectrometry, we demonstrated that the relative abundance of microbial genes for 5α-reductase type 1, but not β-glucuronidase, was positively associated with the circulating DHT/T ratio in women. We also identified *O. splanchnicus* and *P. distasonis* as species positively associated with both the relative abundance of microbial genes for 5α-reductase type 1 and the circulating DHT/T ratio in women. In mechanistic studies, we observed very high DHT/T ratio in intestinal content of female conventionally-raised but not germ-free mice. The DHT/T ratio was 86.9% higher in serum from the portal vein than in inferior vena cava in female mice. These findings demonstrate that the circulating DHT/T ratio is increased by GM 5α-reductase activity in females.

First, we confirmed the finding from a previous small-scale study that premenopausal women have higher circulating DHT/T ratio compared with men,^14^ with similar effect sizes of an approximately three times higher ratio in premenopausal women compared with men. In addition, we extended this previous study by showing that the DHT/T ratio was also substantially higher in postmenopausal women compared with men.

In men, a large meta-analysis recently concluded that T levels decrease with age, while DHT do not,^28^ suggesting that the DHT/T ratio would increase with age. Indeed, an increase with age was found in a study of men with mixed background, but there was no association between age and DHT/T ratio when considering only white men.^29^ Furthermore, two studies in Czech men and American men (black, white, and Hispanic men) did not find changes in DHT/T ratio with age in adulthood.^30^^,^^31^ In women, T seem to increase with age, but slower after 50 years of age than before.^32^ Another study found no difference with age for T but a decline with age for DHT.^33^

Next, we explored the GM for its possible contribution to the relatively high DHT/T ratio in women. Previous experimental studies have suggested that the GM may regulate sex steroids levels by affecting their production, degradation, and reactivation,^10^, ^11^, ^12^, ^13^ proposedly to harvest energy and carbon or create a beneficial microenvironment for themselves. For example, *Thauera* sp. strain GDN1 has been reported to degrade T by ring cleavage, and to be able to use T as its sole carbon source.^12^ Furthermore, *Ruminococcus gnavus* has been described to enhance T synthesis from androgen precursors, an ability that may provide survival benefits that allows the enrichment of *R. gnavus* during androgen deprivation.^11^ In addition, it is well established that the GM can reactivate oestrogens excreted in intestinal content by producing β-glucuronidase that removes their glucuronide moiety, allowing them to be reabsorbed into the circulation.^13^ In a previous study, we demonstrated that the GM also deglucuronidates androgens.^10^ We, therefore, hypothesised that changes in the GM β-glucuronidase activity could affect the androgen reabsorption from the gut and thereby contribute to the high circulating DHT/T ratio in women. Although we observed microbial genes for β-glucuronidase in the GM from all investigated human participants, they were not associated with the circulating DHT/T ratio in humans, suggesting that β-glucuronidase activity is not important for the regulation of the DHT/T ratio in women. Our, alternative hypothesis was that the relatively high circulating DHT/T ratio in women might be influenced by GM 5α-reductase activity. Microbial genes for 5α-reductase type 1 were present in 99.8% of the human participants and in all investigated mice, supporting the notion that GM 5α-reductase activity might be involved in the regulation of the circulating DHT/T ratio. Importantly, we made the observation that the relative abundance of microbial genes for 5α-reductase type 1 was positively associated with the circulating DHT/T ratio in women, and similar strengths of associations were observed in pre- and postmenopausal women. In contrast, we did not find any association between the relative abundance of microbial genes for 5α-reductase type 1 and the circulating DHT/T ratio in men. This discrepancy between men and women may be because men rely less on GM 5α-reductase activity for the circulating DHT/T ratio as males have high levels of testicular-derived DHT and T and also express high levels of 5α-reductase type 2 in reproductive tissues, thereby potentially affecting the circulating DHT/T ratio. In addition, we observed that the relative abundance of microbial genes for GM 5α-reductase type 1 was lower in men compared with women, suggesting that it is regulated in a sex-specific manner. We speculate that the GM functional potential for 5α-reductase type 1 might be regulated in a negative feed-back loop, to be identified, in analogy to the well-known central negative feed-back loops of androgen synthesis.^34^

Furthermore, we aimed to identify the most plausible GM species with a high functional potential for 5α-reductase type 1. We observed that the relative abundances of both *O. splanchnicus* and *P. distasonis* in the GM were independently positively associated with both the relative abundance of microbial genes for 5α-reductase type 1 and the circulating DHT/T ratio in women but not men. A role of these two species for the conversion of T to DHT in females is supported by that the relative abundances of these two GM species were higher in women compared with men. 5α-reductase activity was recently shown *in vitro* and *in vivo* for *Bacteroides uniformis* and several species in the family of *Odoribacteraceae,* to which *O. splanchnicus* belongs.^35^ These findings suggest that GM 5α-reductase type 1 activity, derived from *O. splanchnicus* and *P. distasonis* or highly correlated species, increases the circulating DHT/T ratio in women. However, as we focused on the three species most statistically significantly associated with the relative abundance of microbial genes for 5α-reductase type 1, we cannot exclude that other species may also be of importance. Nevertheless, the associations for the other species are given in Table S3.

However, it should be emphasised that our above-described human association studies, using the large SCAPIS cohort, is descriptive and thus cannot determine causality. To determine the role of the GM 5α-reductase activity for the DHT/T ratio in intestinal content and in the circulation, we performed mechanistic studies in mice. These studies demonstrated that the DHT/T ratio was substantially higher in intestinal contents than in any host tissues of female mice. Furthermore, the DHT/T ratio in the intestinal content was substantially lower in germ-free mice, demonstrating that GM is a major contributor to the functional 5α-reductase activity in intestinal content. Finally, we determined whether the high DHT/T ratio in the intestinal content also influenced the circulating DHT/T ratio in female mice. Importantly, we observed that the DHT/T ratio in serum from the portal vein, draining the intestines, was higher than in serum from the systemic circulation. Together, these mechanistic findings demonstrate that the GM contributes to the high DHT/T ratio in intestinal content with an impact on circulating DHT/T ratio in females.

Based on these human association studies and mechanistic studies, we speculate that the GM may act as an endocrine organ, influencing the androgenic status in females and that the GM 5α-reductase activity might be a potential target for treatments of androgen-related diseases in females. Further studies should determine if men with prostate cancer on androgen deprivation therapy, having no testicular-derived DHT and T, will develop a feminised GM with a high functional potential for 5α-reductase type 1, possibly contributing to the development of castration-resistant prostate cancer.

The current study has several strengths, including the large sample size of the SCAPIS cohort, the translational approach, sensitive and specific mass spectrometry-based methods (LC-MS/MS for human samples and GC–MS/MS for mouse samples) used for androgen quantification, and the metagenome sequencing used for detailed taxonomic and functional characterisation of the GM. However, the current study also has limitations. One limitation is that all participants lived in Sweden and were of mostly Swedish ancestry. Sex steroid levels differ between populations with different ethnic backgrounds and geographical locations.^36^, ^37^, ^38^, ^39^ Therefore, studies in other ethnicities and geographical locations are necessary to determine whether our findings are generalisable. It is well known that antibiotics influence the GM^40^; and antibiotics may also influence sex steroid metabolism.^41^ In the present study, we adjusted the analyses for recent antibiotic use, but similar strengths of the main associations were also observed in sensitivity analyses excluding subjects on recent antibiotic treatment. As questionnaire data was used to determine menopausal status and use of endocrine therapy (corresponding to ATC code L02) and testosterone-5α-reductase inhibitors (corresponding to ATC codes G04CB), we cannot exclude self-reporting bias.

Since the questionnaire did not allow participants to state the regularity of menstruations together with the fact that participants of the SCAPIS cohort were 50–65 years of age, it is possible that some of the premenopausal women (having stated that they had menstruations last year) are in fact perimenopausal. Therefore, this group may not be representative of premenopausal women in general. Furthermore, since the number of premenopausal women was substantially lower than the number of postmenopausal women, the statistical analyses for premenopausal women were less powered than the analyses for post-menopausal women. However, our main analyses compared men and women.

As androgen levels change over the menstrual cycle, it is a limitation with the present study that we for premenopausal women did not have information regarding which phase of the menstrual cycle that the blood sample was taken.

Another limitation is that our human data is observational and, thus, cannot determine causality, even though our experimental work in mice provide mechanistic suggestions. A limitation with the experimental work is that we did not have serum for DHT and T measurements in an experiment with both female and male mice and could therefore not directly compare the DHT/T ratio between sexes in mice. Also, we did not sample the females at a specific cycle stage, and as sex steroids are altered during the cycle and have been shown to interact with the GM,^42^ cycle dependent sex steroid levels could have influenced our findings. Furthermore, we used mice that were between 8 weeks and 6 months, ages that are corresponding to young adult to adult age in humans but not to the older age of the human participants.

The current study focused on gut microbial 5α-reductase and β-glucuronidase as determinators of the circulating DHT/T ratio. However, androgen metabolism is complex, and other mechanisms such as the conversion of androstenedione or androstenediol to T, T to oestradiol or 11-oxygenated androgens, androstanedione to DHT, or increased production of DHT via the backdoor pathway could also influence the DHT/T ratio.^43^^,^^44^ Furthermore, the DHT/T ratio may also be affected by ageing and metabolism in tissues such as muscle and adipose tissue.^45^ To expand the present work, future studies should integrate analyses of these additional mechanisms and more thoroughly examine the interaction between the gut microbiota and host tissues for determining the DHT/T ratio and other aspects of sex steroid metabolism. Similar to the reabsorption of steroids following deglucuronidation by GM glucuronidase, sulphated steroids may also be reabsorbed from the gut following desulfation,^46^ but this mechanism is yet to be explored.

In conclusion, the circulating DHT/T ratio is increased by GM 5α-reductase activity in females. We propose that the GM acts as an endocrine organ influencing the androgenic status in females.

## Contributors

C.O. and L.G. designed the study. C.O., L.Li., K.H., L.La., H.C., K.S., G.B., G.E., J.Ä., M.O.-M., T.F., L.G. collected and/or analysed the data. C.O. and L.G. had access to the data and verified the underlying data, performed the literature search, interpreted the data, and wrote the first draft of the manuscript. All the authors contributed to subsequent drafts of the manuscript, made the decision to submit the manuscript for publication, and read and approved the final version of the manuscript.

## Data sharing statement

The human data underlying this manuscript cannot be shared publicly for legal regulations related to the privacy of individuals that participated in the study. Access to SCAPIS data requires ethical approval from the Swedish Ethical Review Board and approval from the SCAPIS Data access board (https://www.scapis.org/data-access).

The animal data will be shared upon reasonable request to the corresponding author.

## Declaration of interests

C.O. and K.S. are listed as inventors on a patent application regarding the impact of probiotics on bone metabolism and have received research funding for probiotic-related research from Probi AB and Solarea Bio. J.Ä. has served on advisory boards for Astella, AstraZeneca, and Boehringer Ingelheim, and has received lecturing fees from AstraZeneca, Boehringer Ingelheim, and Novartis, unrelated to the present work. None of the other authors declare any conflicts of interest.
