# Supplementary material for: The circulating dihydrotestosterone/testosterone ratio is increased by gut microbial 5α-reductase activity in females
Source: eBioMedicine. 2025 Oct 21;121:105978. doi: 10.1016/j.ebiom.2025.105978 (PMC12589954; doi:10.1016/j.ebiom.2025.105978)
Supplement: Supplementary Figures and Tables [file mmc1.pdf]

SUPPLEMENTAL MATERIALS

The circulating dihydrotestosterone/testosterone ratio is increased by gut microbial 5α-reductase activity in females

CONTENTS

Figure S1	Page 2
Figure S2	Page 3
Table S1	Page 4
Table S2	Page 5
Table S3	Page 6
Table S4	Page 14
Table S5	Page 15

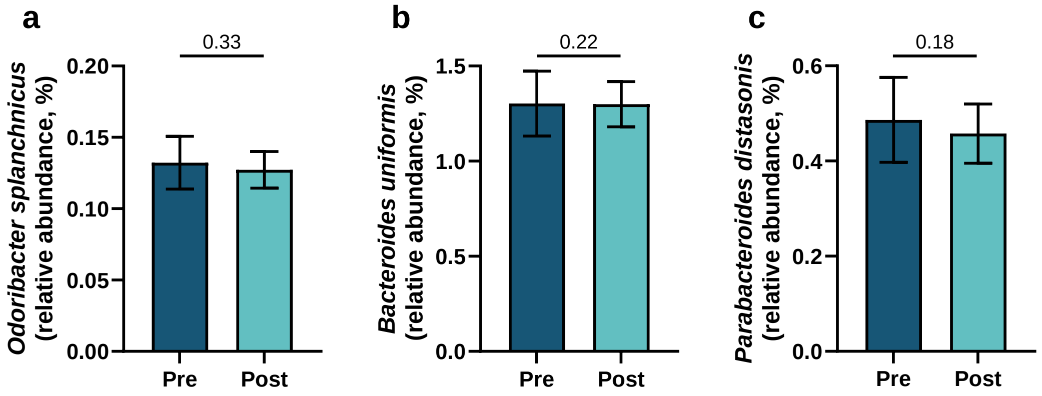

Figure S1. The relative abundances of *Odoribacter splanchnicus, Bacteroides uniformis,* and *Parabacteroides distasonis* do not differ between pre- and postmenopausal women. Relative abundance of (a) *Odoribacter splanchnicus*, (b) *Bacteroides uniformis*, and (c) *Parabacteroides distasonis* in pre- and postmenopausal women. ANCOVA on inverse rank transformed data adjusted for age, extraction plate, and antibiotic use. Data are presented as untransformed estimated marginal means ± 95% CI. n=561 premenopausal women and 2,336 postmenopausal women.

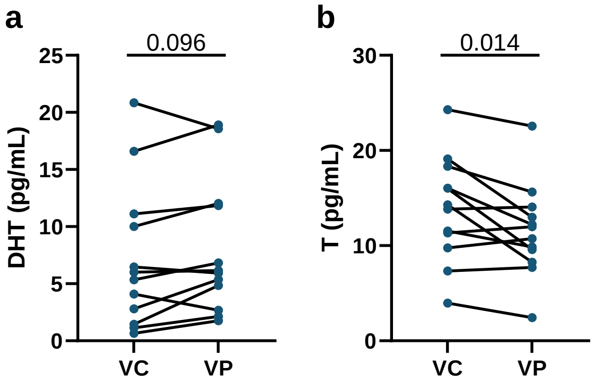

Figure S2. DHT and T levels in serum from vena cava and vena porta of female mice. The (a) DHT and (b) T levels in serum from inferior vena cava (VC) and vena porta (VP) in female mice. Paired samples t-test of untransformed data. Data are presented as scatter plots of untransformed data, with the lines indicating samples from individual mice. n=12

Table S1. Detailed sex hormone-related exclusions

Reason for exclusion	Method for exclusion	Number
Polycystic ovary syndrome	questionnaire data	39
Unclear menopausal state	questionnaire data	815
Use of medications that interfere with sex steroid levels		
Sex hormones and modulators of the genital system, ATC code G03	registered dispensed prescriptions	986
Endocrine therapy, ATC code L02	questionnaire data	33
Testosterone-5-alpha reductase inhibitors, ATC code G04CB	questionnaire data	40
high sex steroid levels for their sex and menopausal state		
postmenopausal women with circulating oestradiol >20 pg/ml	hormone measurement	89
men with circulating oestradiol > 63.7 pg/ml (mean + 5 SD)	hormone measurement	8
men with circulating progesterone > 350.3 pg/ml (mean + 5SD)	hormone measurement	8
premenopausal women with circulating testosterone >2902 pg/ml (mean + 5SD)	hormone measurement	4
postmenopausal women with circulating testosterone >1281 pg/ml (mean+ 5SD)	hormone measurement	10

SD, standard deviation

Table S2. Relative abundances of *Odoribacter splanchnicus* , *Bacteroides uniformis* , and *Parabacteroides distasonis* in women and men, with or without adjustment for country of birth and diet

	**Estimated marginal mean (95% CI)**		**P**
	**Women**	**Men**	
**No adjustment for country of birth and diet**			
Odoribacter splanchnicus	0.13 (0.12-0.14)	0.11 (0.10-0.12)	2.4E-10
Bacteroides uniformis	1.27 (1.18-1.35)	1.20 (1.13-1.28)	2.5E-03
Parabacteroides distasonis	0.44 (0.40-0.48)	0.40 (0.36-0.43)	7.8E-03
**Adjustment for country of birth and diet**			
Odoribacter splanchnicus	0.12 (0.11-0.13)	0.11 (0.10-0.12)	3.1E-07
Bacteroides uniformis	1.18 (1.09-1.27)	1.14 (1.06-1.23)	7.4E-03
Parabacteroides distasonis	0.42 (0.38-0.46)	0.37 (0.33-0.41)	2.2E-04

ANCOVA on inverse rank transformed data adjusted for age, extraction plate, and antibiotic use, with or without further adjustment for diet (energy intake in kcal/day and intake of fat, fibres, protein, and carbohydrates in grams/day) and country of birth (born in Sweden; yes/no). Data (relative abundances of species) are presented as untransformed estimated marginal means ± 95% confidence intervals. n=6965

Table S3. Associations between gut microbial species and relative abundance of microbial genes for 5α-reductase type 1 in women, premenopausal wome, postmenopausal women, and men

Linear regression models of inverse rank transformed relative abundance of gut microbial species as outcomes and inverse rank transformed relative abundance of microbial genes for 5α-reductase type 1 as exposure, adjusted for age, extraction plate, antibiotic use (and menopausal state for women). Data are beta and standard error of the mean (SE). Betas are expressed as standard deviation (SD) change in relative abundance of 5a-reductase type 1 per SD change in relative abundance of gut microbial species. All the species in this table is from the the bacteria superkingdom. The table is sorted on P values for women. Prev, prevalence

						Women					Premenopausal women					Postmenopausal women					Men				
**Species**	**Genus**	**Family**	**Order**	**Class**	**Phylum**	**β**	**SE**	**P**	**n**	**Prev**	**β**	**SE**	**P**	**n**	**Prev**	**β**	**SE**	**P**	**n**	**Prev**	**β**	**SE**	**P**	**n**	**Prev**
Odoribacter splanchnicus	Odoribacter	Marinifilaceae	Bacteroidales	Bacteroidia	Bacteroidota	0.536	0.016	9.4E-200	2897	92.6	0.528	0.042	4.1E-31	561	93.4	0.542	0.018	2.6E-164	2336	92.4	0.529	0.013	1.4E-296	4338	91.4
Bacteroides uniformis	Bacteroides	Bacteroidaceae	Bacteroidales	Bacteroidia	Bacteroidota	0.502	0.016	7.1E-177	2897	95.9	0.545	0.041	1.1E-33	561	97.3	0.499	0.018	5.9E-140	2336	95.5	0.566	0.013	<2.2E-308	4338	97.1
Parabacteroides distasonis	Parabacteroides	Tannerellaceae	Bacteroidales	Bacteroidia	Bacteroidota	0.471	0.017	1.3E-149	2897	92.8	0.487	0.043	1.1E-26	561	92.9	0.473	0.019	1.1E-120	2336	92.8	0.506	0.013	1.6E-268	4338	93.5
Alistipes putredinis	Alistipes	Rikenellaceae	Bacteroidales	Bacteroidia	Bacteroidota	0.462	0.017	1.0E-144	2897	90.3	0.474	0.042	4.6E-26	561	90.4	0.457	0.019	7.7E-113	2336	90.3	0.485	0.014	2.3E-243	4338	89.0
Barnesiella intestinihominis	Barnesiella	Barnesiellaceae	Bacteroidales	Bacteroidia	Bacteroidota	0.387	0.018	5.8E-94	2897	82.8	0.347	0.046	2.1E-13	561	83.6	0.400	0.020	1.9E-80	2336	82.6	0.403	0.014	4.9E-157	4338	82.2
Alistipes onderdonkii	Alistipes	Rikenellaceae	Bacteroidales	Bacteroidia	Bacteroidota	0.377	0.018	1.1E-90	2897	86.3	0.401	0.045	8.6E-18	561	87.9	0.382	0.020	1.1E-74	2336	85.9	0.425	0.014	1.9E-179	4338	86.5
Lawsonibacter sp900066645	Lawsonibacter	Oscillospiraceae	Oscillospirales	Clostridia	Bacillota A	0.375	0.018	1.6E-90	2897	92.2	0.338	0.046	1.5E-12	561	92.3	0.379	0.020	1.7E-74	2336	92.2	0.408	0.014	3.1E-165	4338	90.7
Phocaeicola vulgatus	Phocaeicola	Bacteroidaceae	Bacteroidales	Bacteroidia	Bacteroidota	0.368	0.018	1.4E-84	2897	84.1	0.411	0.044	9.9E-19	561	86.1	0.364	0.021	6.1E-66	2336	83.6	0.501	0.014	8.5E-260	4338	89.1
Phocaeicola dorei	Phocaeicola	Bacteroidaceae	Bacteroidales	Bacteroidia	Bacteroidota	0.367	0.019	4.9E-78	2897	72.4	0.394	0.047	9.2E-16	561	71.1	0.358	0.021	6.9E-60	2336	72.6	0.329	0.015	8.5E-97	4338	75.0
Bacteroides caccae	Bacteroides	Bacteroidaceae	Bacteroidales	Bacteroidia	Bacteroidota	0.356	0.019	4.8E-75	2897	76.8	0.358	0.048	6.9E-13	561	77.4	0.366	0.021	1.3E-63	2336	76.7	0.385	0.015	1.5E-140	4338	80.2
Alistipes communis	Alistipes	Rikenellaceae	Bacteroidales	Bacteroidia	Bacteroidota	0.332	0.019	4.3E-68	2897	80.9	0.361	0.048	1.9E-13	561	79.7	0.332	0.021	2.9E-54	2336	81.3	0.309	0.015	1.0E-88	4338	80.8
Bacteroides ovatus	Bacteroides	Bacteroidaceae	Bacteroidales	Bacteroidia	Bacteroidota	0.325	0.019	4.1E-65	2897	84.3	0.280	0.048	9.7E-09	561	82.4	0.332	0.021	1.1E-54	2336	84.8	0.405	0.014	1.1E-158	4338	86.7
Dysosmobacter welbionis	Dysosmobacter	Oscillospiraceae	Oscillospirales	Clostridia	Bacillota A	0.304	0.018	1.5E-61	2897	98.4	0.288	0.044	1.9E-10	561	99.3	0.308	0.020	8.0E-51	2336	98.2	0.325	0.014	4.5E-105	4338	98.0
Alistipes shahii	Alistipes	Rikenellaceae	Bacteroidales	Bacteroidia	Bacteroidota	0.313	0.019	1.6E-59	2897	79.8	0.286	0.048	3.8E-09	561	81.8	0.320	0.021	5.7E-50	2336	79.3	0.348	0.015	5.2E-114	4338	80.5
Bacteroides thetaiotaomicron	Bacteroides	Bacteroidaceae	Bacteroidales	Bacteroidia	Bacteroidota	0.302	0.019	6.6E-54	2897	80.6	0.302	0.048	6.0E-10	561	83.6	0.300	0.022	1.8E-42	2336	79.9	0.353	0.015	7.3E-113	4338	80.9
Parabacteroides merdae	Parabacteroides	Tannerellaceae	Bacteroidales	Bacteroidia	Bacteroidota	0.301	0.019	6.1E-53	2897	74.8	0.238	0.052	6.9E-06	561	73.1	0.312	0.021	2.9E-46	2336	75.3	0.344	0.015	2.1E-109	4338	79.4
Borkfalkia ceftriaxoniphila	Borkfalkia	Borkfalkiaceae	Christensenellales	Clostridia	Bacillota A	0.319	0.021	1.5E-50	2897	52.5	0.321	0.052	1.2E-09	561	55.4	0.321	0.023	4.2E-41	2336	51.8	0.321	0.017	2.0E-73	4338	44.5
Alistipes finegoldii	Alistipes	Rikenellaceae	Bacteroidales	Bacteroidia	Bacteroidota	0.284	0.019	1.8E-48	2897	80.5	0.285	0.048	5.2E-09	561	79.7	0.287	0.021	5.1E-40	2336	80.7	0.308	0.015	2.4E-86	4338	78.5
Lawsonibacter asaccharolyticus	Lawsonibacter	Oscillospiraceae	Oscillospirales	Clostridia	Bacillota A	0.262	0.018	1.1E-45	2897	97.8	0.272	0.046	5.1E-09	561	98.0	0.258	0.020	1.3E-35	2336	97.8	0.309	0.014	3.7E-96	4338	97.7
Intestinimonas butyriciproducens	Intestinimonas	Oscillospiraceae	Oscillospirales	Clostridia	Bacillota A	0.256	0.018	2.3E-42	2897	91.0	0.255	0.047	8.3E-08	561	91.4	0.257	0.021	2.6E-34	2336	90.8	0.289	0.015	2.3E-80	4338	86.8
Dysosmobacter faecalis	Dysosmobacter	Oscillospiraceae	Oscillospirales	Clostridia	Bacillota A	0.253	0.018	6.5E-42	2897	96.7	0.202	0.047	2.0E-05	561	97.5	0.261	0.021	7.6E-36	2336	96.5	0.301	0.015	5.2E-90	4338	95.3
Bacteroides cellulosilyticus	Bacteroides	Bacteroidaceae	Bacteroidales	Bacteroidia	Bacteroidota	0.278	0.020	1.6E-41	2897	61.9	0.245	0.051	2.0E-06	561	60.1	0.288	0.023	8.1E-36	2336	62.4	0.301	0.016	5.5E-76	4338	64.8
Flavonifractor plautii	Flavonifractor	Oscillospiraceae	Oscillospirales	Clostridia	Bacillota A	0.246	0.019	1.3E-38	2897	92.6	0.212	0.048	1.2E-05	561	93.8	0.249	0.021	3.7E-32	2336	92.3	0.279	0.015	7.9E-76	4338	94.8
CAKTXU01 sp934615635	CAKTXU01	Acutalibacteraceae	Oscillospirales	Clostridia	Bacillota A	0.252	0.020	1.6E-36	2897	71.2	0.246	0.051	1.7E-06	561	70.6	0.255	0.022	3.4E-30	2336	71.3	0.259	0.016	1.2E-57	4338	68.6
UMGS856 sp900546265	UMGS856	Acutalibacteraceae	Oscillospirales	Clostridia	Bacillota A	0.235	0.019	1.5E-35	2897	90.4	0.209	0.047	1.1E-05	561	90.4	0.250	0.021	2.7E-32	2336	90.4	0.240	0.015	1.7E-55	4338	87.5
Bilophila wadsworthia	Bilophila	Desulfovibrionaceae	Desulfovibrionales	Desulfovibrionia	Desulfobacterota	0.236	0.019	2.9E-35	2897	88.2	0.194	0.048	5.6E-05	561	89.8	0.245	0.021	2.0E-30	2336	87.8	0.237	0.015	9.7E-54	4338	87.7
Dysosmobacter sp944387035	Dysosmobacter	Oscillospiraceae	Oscillospirales	Clostridia	Bacillota A	0.240	0.019	1.4E-34	2897	80.7	0.201	0.048	4.1E-05	561	80.7	0.250	0.022	3.7E-30	2336	80.7	0.273	0.015	1.7E-67	4338	78.6
Choladousia sp902363135	Choladousia	Lachnospiraceae	Lachnospirales	Clostridia	Bacillota A	-0.227	0.019	2.4E-32	2897	87.8	-0.248	0.049	4.6E-07	561	89.3	-0.229	0.021	1.8E-26	2336	87.5	-0.248	0.015	2.1E-58	4338	87.7
Dorea A longicatena	Dorea A	Lachnospiraceae	Lachnospirales	Clostridia	Bacillota A	-0.221	0.019	5.5E-31	2897	86.7	-0.227	0.048	2.7E-06	561	87.5	-0.226	0.021	3.6E-26	2336	86.5	-0.258	0.015	5.9E-64	4338	88.9
Bacteroides fragilis	Bacteroides	Bacteroidaceae	Bacteroidales	Bacteroidia	Bacteroidota	0.251	0.021	8.0E-31	2897	48.4	0.251	0.055	6.2E-06	561	48.1	0.255	0.024	9.4E-26	2336	48.5	0.246	0.018	2.3E-43	4338	45.2
Pseudoflavonifractor capillosus	Pseudoflavonifractor	Oscillospiraceae	Oscillospirales	Clostridia	Bacillota A	0.246	0.022	9.1E-29	2897	45.8	0.261	0.056	4.2E-06	561	44.6	0.238	0.025	7.4E-22	2336	46.1	0.225	0.018	2.3E-36	4338	45.4
Angelakisella massiliensis	Angelakisella	Ruminococcaceae	Oscillospirales	Clostridia	Bacillota A	0.262	0.024	4.8E-28	2897	32.3	0.235	0.059	9.0E-05	561	31.0	0.269	0.026	7.6E-24	2336	32.6	0.277	0.019	4.8E-46	4338	30.0
Rothia dentocariosa	Rothia	Micrococcaceae	Actinomycetales	Actinomycetia	Actinomycetota	-0.228	0.021	4.3E-27	2897	58.1	-0.250	0.054	4.6E-06	561	54.2	-0.227	0.023	3.5E-22	2336	59.1	-0.185	0.017	8.8E-27	4338	55.2
Alistipes A ihumii	Alistipes A	Rikenellaceae	Bacteroidales	Bacteroidia	Bacteroidota	0.216	0.020	8.3E-27	2897	68.5	0.219	0.051	2.2E-05	561	69.3	0.218	0.022	6.0E-22	2336	68.3	0.203	0.016	6.3E-35	4338	65.7
Parasutterella excrementihominis	Parasutterella	Burkholderiaceae A	Burkholderiales	Gammaproteobacteria	Pseudomonadota	0.221	0.020	8.8E-27	2897	62.8	0.263	0.051	3.9E-07	561	64.3	0.220	0.023	9.7E-22	2336	62.5	0.288	0.016	4.9E-66	4338	58.9
Enterocloster lavalensis	Enterocloster	Lachnospiraceae	Lachnospirales	Clostridia	Bacillota A	0.216	0.020	1.7E-26	2897	67.9	0.178	0.053	8.5E-04	561	65.1	0.232	0.022	1.4E-24	2336	68.6	0.300	0.016	5.2E-75	4338	65.2
Actinomyces sp000220835	Actinomyces	Actinomycetaceae	Actinomycetales	Actinomycetia	Actinomycetota	-0.247	0.023	1.7E-26	2897	37.7	-0.294	0.057	3.8E-07	561	40.5	-0.244	0.026	5.4E-21	2336	37.0	-0.218	0.018	4.3E-32	4338	38.6
Romboutsia timonensis	Romboutsia	Peptostreptococcaceae	Peptostreptococcales	Clostridia	Bacillota A	-0.201	0.019	3.1E-26	2897	92.4	-0.191	0.047	5.3E-05	561	93.2	-0.201	0.021	2.8E-21	2336	92.2	-0.192	0.015	1.1E-35	4338	92.2
Lentihominibacter sp905193765	Lentihominibacter	Anaerovoracaceae	Peptostreptococcales	Clostridia	Bacillota A	-0.230	0.022	5.2E-26	2897	53.6	-0.240	0.054	1.3E-05	561	53.3	-0.236	0.024	5.4E-22	2336	53.7	-0.199	0.017	5.8E-31	4338	57.3
Bariatricus comes	Bariatricus	Lachnospiraceae	Lachnospirales	Clostridia	Bacillota A	-0.202	0.019	7.8E-26	2897	86.9	-0.172	0.049	4.5E-04	561	87.5	-0.216	0.021	1.3E-23	2336	86.7	-0.219	0.015	4.3E-46	4338	89.3
Coprobacter fastidiosus	Coprobacter	Coprobacteraceae	Bacteroidales	Bacteroidia	Bacteroidota	0.218	0.021	9.2E-26	2897	59.8	0.203	0.053	1.6E-04	561	58.5	0.227	0.023	1.3E-22	2336	60.1	0.294	0.016	3.2E-71	4338	61.8
Avimonas A narfia	Avimonas A	Acutalibacteraceae	Oscillospirales	Clostridia	Bacillota A	0.206	0.020	9.2E-25	2897	71.6	0.172	0.051	8.9E-04	561	71.7	0.215	0.022	8.3E-22	2336	71.6	0.240	0.016	5.9E-49	4338	67.5
Anaerotruncus colihominis	Anaerotruncus	Ruminococcaceae	Oscillospirales	Clostridia	Bacillota A	0.203	0.020	2.7E-24	2897	74.7	0.166	0.050	9.8E-04	561	77.2	0.209	0.022	8.7E-21	2336	74.1	0.202	0.016	5.9E-37	4338	76.2
Mogibacterium diversum	Mogibacterium	Anaerovoracaceae	Peptostreptococcales	Clostridia	Bacillota A	-0.201	0.020	1.0E-23	2897	73.2	-0.308	0.053	1.4E-08	561	75.0	-0.187	0.022	7.4E-17	2336	72.8	-0.202	0.016	6.0E-36	4338	73.1
Scatosoma sp900555925	Scatosoma	Borkfalkiaceae	Christensenellales	Clostridia	Bacillota A	0.210	0.021	1.6E-23	2897	59.9	0.213	0.051	4.1E-05	561	62.6	0.216	0.023	3.4E-20	2336	59.2	0.227	0.017	1.8E-40	4338	56.7
Pauljensenia sp000278725	Pauljensenia	Actinomycetaceae	Actinomycetales	Actinomycetia	Actinomycetota	-0.199	0.020	2.2E-23	2897	74.2	-0.263	0.050	2.0E-07	561	75.6	-0.187	0.022	4.6E-17	2336	73.9	-0.185	0.016	3.7E-31	4338	77.8
Alistipes A indistinctus	Alistipes A	Rikenellaceae	Bacteroidales	Bacteroidia	Bacteroidota	0.199	0.020	2.6E-23	2897	73.2	0.221	0.051	2.1E-05	561	74.0	0.201	0.022	3.2E-19	2336	73.1	0.201	0.016	1.6E-35	4338	71.0
Blautia A luti	Blautia A	Lachnospiraceae	Lachnospirales	Clostridia	Bacillota A	-0.206	0.021	3.3E-23	2897	64.9	-0.204	0.053	1.4E-04	561	66.5	-0.212	0.023	5.5E-20	2336	64.5	-0.192	0.016	3.5E-32	4338	69.7
Scatosoma sp003150575	Scatosoma	Borkfalkiaceae	Christensenellales	Clostridia	Bacillota A	0.218	0.022	3.6E-23	2897	48.9	0.342	0.055	9.6E-10	561	49.7	0.205	0.024	7.2E-17	2336	48.7	0.236	0.018	7.2E-38	4338	40.6
NSJ-51 sp900549795	NSJ-51	Oscillospiraceae	Oscillospirales	Clostridia	Bacillota A	0.195	0.020	5.8E-23	2897	78.7	0.211	0.049	1.7E-05	561	80.7	0.193	0.022	2.9E-18	2336	78.2	0.220	0.016	2.9E-42	4338	72.4
Bacteroides xylanisolvens	Bacteroides	Bacteroidaceae	Bacteroidales	Bacteroidia	Bacteroidota	0.199	0.020	6.3E-23	2897	69.1	0.195	0.051	1.4E-04	561	68.6	0.201	0.022	6.9E-19	2336	69.2	0.255	0.016	5.5E-57	4338	72.2
Actinomyces dentalis	Actinomyces	Actinomycetaceae	Actinomycetales	Actinomycetia	Actinomycetota	-0.233	0.024	1.5E-22	2897	33.9	-0.259	0.059	1.5E-05	561	35.3	-0.230	0.027	9.3E-18	2336	33.6	-0.220	0.018	4.8E-33	4338	40.0
Tidjanibacter inops	Tidjanibacter	Rikenellaceae	Bacteroidales	Bacteroidia	Bacteroidota	0.205	0.021	3.6E-22	2897	55.6	0.212	0.053	7.4E-05	561	55.1	0.204	0.023	5.3E-18	2336	55.7	0.222	0.017	9.1E-37	4338	48.5

						Women					Premenopausal women					Postmenopausal women					Men				
**Species**	**Genus**	**Family**	**Order**	**Class**	**Phylum**	**β**	**SE**	**P**	**n**	**Prev**	**β**	**SE**	**P**	**n**	**Prev**	**β**	**SE**	**P**	**n**	**Prev**	**β**	**SE**	**P**	**n**	**Prev**
Hungatella effluvii	Hungatella	Lachnospiraceae	Lachnospirales	Clostridia	Bacillota A	0.214	0.022	4.1E-22	2897	46.6	0.096	0.057	9.4E-02	561	48.1	0.238	0.024	4.8E-22	2336	46.2	0.170	0.018	1.1E-21	4338	46.8
Slackia A isoflavoniconvertens	Slackia A	Eggerthellaceae	Coriobacteriales	Coriobacteriia	Actinomycetota	-0.217	0.023	1.4E-21	2897	40.2	-0.209	0.057	2.6E-04	561	39.0	-0.221	0.025	2.8E-18	2336	40.5	-0.207	0.017	3.6E-32	4338	49.0
Corynebacterium durum	Corynebacterium	Mycobacteriaceae	Mycobacteriales	Actinomycetia	Actinomycetota	-0.223	0.023	2.3E-21	2897	35.5	-0.258	0.056	6.0E-06	561	37.3	-0.218	0.026	1.6E-16	2336	35.0	-0.220	0.019	7.7E-32	4338	36.9
Enterocloster citroniae	Enterocloster	Lachnospiraceae	Lachnospirales	Clostridia	Bacillota A	0.187	0.020	3.3E-21	2897	78.0	0.147	0.050	3.3E-03	561	77.5	0.193	0.022	2.7E-18	2336	78.2	0.212	0.016	3.8E-41	4338	79.2
Mailhella merdigallinarum	Mailhella	Desulfovibrionaceae	Desulfovibrionales	Desulfovibrionia	Desulfobacterota	0.202	0.022	1.4E-20	2897	50.0	0.205	0.055	1.9E-04	561	51.0	0.205	0.024	4.3E-17	2336	49.7	0.212	0.018	3.1E-32	4338	43.5
Enterocloster bolteae	Enterocloster	Lachnospiraceae	Lachnospirales	Clostridia	Bacillota A	0.186	0.020	1.5E-20	2897	74.8	0.123	0.051	1.6E-02	561	75.9	0.197	0.022	1.6E-18	2336	74.6	0.206	0.016	1.1E-37	4338	75.5
Limivivens sp900066135	Limivivens	Lachnospiraceae	Lachnospirales	Clostridia	Bacillota A	-0.190	0.020	2.6E-20	2897	65.8	-0.226	0.051	1.1E-05	561	67.7	-0.185	0.023	7.8E-16	2336	65.4	-0.220	0.016	1.3E-41	4338	69.9
Dorea formicigenerans	Dorea	Lachnospiraceae	Lachnospirales	Clostridia	Bacillota A	-0.174	0.019	2.7E-20	2897	97.1	-0.237	0.047	7.6E-07	561	97.5	-0.172	0.021	2.8E-16	2336	97.0	-0.181	0.015	3.6E-33	4338	97.6
Enterocloster aldenensis	Enterocloster	Lachnospiraceae	Lachnospirales	Clostridia	Bacillota A	0.196	0.021	1.1E-19	2897	53.5	0.097	0.056	8.4E-02	561	52.4	0.217	0.024	2.0E-19	2336	53.7	0.199	0.017	4.4E-31	4338	56.7
HGM11616 sp900761075	HGM11616	Christensenellaceae	Christensenellales	Clostridia	Bacillota A	0.178	0.019	1.2E-19	2897	79.7	0.193	0.049	9.6E-05	561	81.8	0.172	0.022	6.8E-15	2336	79.2	0.237	0.016	2.1E-50	4338	76.6
Diplocloster agilis	Diplocloster	Lachnospiraceae	Lachnospirales	Clostridia	Bacillota A	0.182	0.020	1.6E-19	2897	74.1	0.159	0.050	1.7E-03	561	73.1	0.185	0.022	1.7E-16	2336	74.3	0.161	0.016	9.9E-23	4338	68.3
Oribacterium sinus	Oribacterium	Lachnospiraceae	Lachnospirales	Clostridia	Bacillota A	-0.205	0.023	7.6E-19	2897	36.8	-0.229	0.058	9.1E-05	561	36.9	-0.206	0.026	1.9E-15	2336	36.7	-0.172	0.019	5.8E-20	4338	36.1
CAG-177 sp003514385	CAG-177	Acutalibacteraceae	Oscillospirales	Clostridia	Bacillota A	-0.199	0.022	1.5E-18	2897	42.7	-0.156	0.058	7.2E-03	561	43.7	-0.210	0.025	9.4E-17	2336	42.5	-0.146	0.018	5.0E-16	4338	45.1
Dielma fastidiosa	Dielma	Erysipelotrichaceae	Erysipelotrichales	Bacilli	Bacillota	0.185	0.021	4.9E-18	2897	53.6	0.173	0.055	1.7E-03	561	53.8	0.195	0.024	3.9E-16	2336	53.6	0.162	0.017	3.2E-21	4338	53.4
Peptidiphaga sp000466165	Peptidiphaga	Actinomycetaceae	Actinomycetales	Actinomycetia	Actinomycetota	-0.207	0.024	5.1E-18	2897	33.4	-0.250	0.058	1.9E-05	561	37.6	-0.199	0.027	1.7E-13	2336	32.4	-0.170	0.019	2.2E-19	4338	35.9
Intestinimonas massiliensis	Intestinimonas	Oscillospiraceae	Oscillospirales	Clostridia	Bacillota A	0.171	0.020	7.0E-18	2897	75.4	0.160	0.051	1.6E-03	561	75.9	0.178	0.022	1.0E-15	2336	75.2	0.188	0.016	4.5E-30	4338	66.4
Mediterraneibacter faecis	Mediterraneibacter	Lachnospiraceae	Lachnospirales	Clostridia	Bacillota A	-0.165	0.019	7.8E-18	2897	87.8	-0.098	0.048	4.3E-02	561	89.7	-0.183	0.021	1.7E-17	2336	87.3	-0.160	0.015	1.6E-25	4338	90.4
Limivivens sp900543575	Limivivens	Lachnospiraceae	Lachnospirales	Clostridia	Bacillota A	-0.163	0.019	1.8E-17	2897	89.3	-0.189	0.048	1.1E-04	561	90.4	-0.166	0.021	9.9E-15	2336	89.0	-0.143	0.015	1.8E-20	4338	89.6
Enterenecus sp900549885	Enterenecus	Oscillospiraceae	Oscillospirales	Clostridia	Bacillota A	0.166	0.019	1.8E-17	2897	81.0	0.134	0.050	7.3E-03	561	81.8	0.171	0.022	5.3E-15	2336	80.9	0.203	0.016	2.4E-37	4338	75.5
Instestinimonas A gabonensis	Instestinimonas A	Oscillospiraceae	Oscillospirales	Clostridia	Bacillota A	0.161	0.019	3.7E-17	2897	88.0	0.162	0.048	8.8E-04	561	89.7	0.161	0.021	7.8E-14	2336	87.6	0.192	0.015	6.5E-35	4338	85.7
Pauljensenia sp000758755	Pauljensenia	Actinomycetaceae	Actinomycetales	Actinomycetia	Actinomycetota	-0.197	0.023	3.7E-17	2897	35.7	-0.283	0.057	1.2E-06	561	38.5	-0.175	0.026	2.8E-11	2336	35.0	-0.192	0.019	1.5E-24	4338	37.5
Coprococcus A catus A	Coprococcus A	Lachnospiraceae	Lachnospirales	Clostridia	Bacillota A	-0.162	0.019	5.7E-17	2897	87.2	-0.180	0.048	2.2E-04	561	88.2	-0.160	0.022	1.7E-13	2336	87.0	-0.123	0.016	3.7E-15	4338	88.5
UMGS1623 sp934647945	UMGS1623	Acutalibacteraceae	Oscillospirales	Clostridia	Bacillota A	0.170	0.020	9.2E-17	2897	66.7	0.110	0.054	4.3E-02	561	63.8	0.186	0.023	2.3E-16	2336	67.4	0.194	0.017	8.8E-31	4338	59.7
Scatosoma sp900552625	Scatosoma	Borkfalkiaceae	Christensenellales	Clostridia	Bacillota A	0.179	0.022	1.6E-16	2897	51.6	0.176	0.053	9.7E-04	561	55.3	0.182	0.024	6.9E-14	2336	50.8	0.182	0.018	1.3E-23	4338	43.4
Avimicrobium caecorum	Avimicrobium	Ruminococcaceae	Oscillospirales	Clostridia	Bacillota A	0.163	0.020	1.7E-16	2897	76.7	0.183	0.049	2.4E-04	561	77.5	0.160	0.022	7.6E-13	2336	76.5	0.192	0.016	1.3E-33	4338	76.0
Anaerobutyricum hallii	Anaerobutyricum	Lachnospiraceae	Lachnospirales	Clostridia	Bacillota A	-0.156	0.019	2.5E-16	2897	94.3	-0.201	0.049	4.8E-05	561	93.8	-0.149	0.021	2.9E-12	2336	94.4	-0.145	0.015	2.0E-21	4338	95.4
Blautia A sp900066505	Blautia A	Lachnospiraceae	Lachnospirales	Clostridia	Bacillota A	-0.159	0.019	3.2E-16	2897	85.8	-0.114	0.049	1.9E-02	561	87.0	-0.165	0.022	3.2E-14	2336	85.6	-0.149	0.015	1.1E-21	4338	88.8
Anaerotruncus rubiinfantis	Anaerotruncus	Ruminococcaceae	Oscillospirales	Clostridia	Bacillota A	0.160	0.020	3.3E-16	2897	80.7	0.201	0.049	5.4E-05	561	81.5	0.152	0.022	4.6E-12	2336	80.5	0.139	0.016	4.1E-18	4338	76.5
Limivivens sp905214955	Limivivens	Lachnospiraceae	Lachnospirales	Clostridia	Bacillota A	-0.162	0.020	5.2E-16	2897	77.2	-0.118	0.050	1.9E-02	561	78.4	-0.176	0.022	4.8E-15	2336	76.9	-0.151	0.016	4.3E-21	4338	77.2
HGM14224 sp900761905	HGM14224	HGM14224	DTUO25	SHA-98	Bacillota G	0.175	0.021	5.7E-16	2897	52.8	0.199	0.055	3.5E-04	561	51.9	0.180	0.024	1.0E-13	2336	53.0	0.185	0.018	1.6E-24	4338	43.9
Butyricimonas paravirosa	Butyricimonas	Marinifilaceae	Bacteroidales	Bacteroidia	Bacteroidota	0.193	0.024	7.7E-16	2897	32.0	0.176	0.060	3.5E-03	561	35.7	0.197	0.027	2.5E-13	2336	31.2	0.232	0.019	8.8E-33	4338	30.5
Clostridium Q symbiosum	Clostridium Q	Lachnospiraceae	Lachnospirales	Clostridia	Bacillota A	0.179	0.022	9.4E-16	2897	45.4	0.067	0.058	2.5E-01	561	44.0	0.205	0.025	1.3E-16	2336	45.7	0.216	0.018	2.1E-33	4338	46.2
CAG-170 sp000436735	CAG-170	Oscillospiraceae	Oscillospirales	Clostridia	Bacillota A	-0.181	0.023	1.5E-15	2897	40.8	-0.166	0.058	4.4E-03	561	40.5	-0.188	0.025	1.3E-13	2336	40.9	-0.150	0.019	7.8E-16	4338	37.6
Oliverpabstia intestinalis	Oliverpabstia	Lachnospiraceae	Lachnospirales	Clostridia	Bacillota A	-0.158	0.020	1.6E-15	2897	78.2	-0.182	0.050	3.1E-04	561	79.3	-0.160	0.022	5.1E-13	2336	77.9	-0.145	0.016	2.8E-20	4338	81.4
Butyricimonas virosa	Butyricimonas	Marinifilaceae	Bacteroidales	Bacteroidia	Bacteroidota	0.170	0.022	3.9E-15	2897	51.2	0.118	0.057	3.9E-02	561	46.2	0.193	0.024	8.1E-16	2336	52.4	0.174	0.017	3.1E-24	4338	55.0
Gemella sanguinis	Gemella	Gemellaceae	Staphylococcales	Bacilli	Bacillota	-0.172	0.022	4.0E-15	2897	51.7	-0.204	0.056	2.8E-04	561	49.4	-0.168	0.024	5.3E-12	2336	52.2	-0.140	0.018	2.4E-15	4338	50.0
Hungatella hathewayi	Hungatella	Lachnospiraceae	Lachnospirales	Clostridia	Bacillota A	0.165	0.021	4.2E-15	2897	60.2	0.153	0.053	4.0E-03	561	59.2	0.167	0.023	1.2E-12	2336	60.4	0.214	0.017	1.2E-36	4338	57.4
Sutterella wadsworthensis	Sutterella	Burkholderiaceae A	Burkholderiales	Gammaproteobacteria	Pseudomonadota	0.165	0.021	6.9E-15	2897	57.9	0.136	0.054	1.2E-02	561	59.0	0.165	0.024	4.0E-12	2336	57.6	0.270	0.016	1.6E-60	4338	64.9
Olsenella F sp001189515	Olsenella F	Atopobiaceae	Coriobacteriales	Coriobacteriia	Actinomycetota	-0.187	0.024	1.0E-14	2897	31.0	-0.190	0.061	1.9E-03	561	33.5	-0.197	0.027	4.8E-13	2336	30.4	-0.211	0.019	3.1E-29	4338	36.2
Bacteroides nordii	Bacteroides	Bacteroidaceae	Bacteroidales	Bacteroidia	Bacteroidota	0.168	0.022	2.5E-14	2897	46.9	0.167	0.056	2.7E-03	561	47.2	0.168	0.024	8.5E-12	2336	46.8	0.210	0.018	6.2E-32	4338	44.2
Mediterraneibacter A butyricigenes	Mediterraneibacter A	Lachnospiraceae	Lachnospirales	Clostridia	Bacillota A	-0.147	0.019	2.8E-14	2897	87.0	-0.226	0.048	3.3E-06	561	88.6	-0.128	0.022	3.3E-09	2336	86.6	-0.137	0.015	1.5E-18	4338	87.2
Blautia A sp900066335	Blautia A	Lachnospiraceae	Lachnospirales	Clostridia	Bacillota A	-0.153	0.020	8.4E-14	2897	66.9	-0.233	0.051	6.0E-06	561	67.9	-0.144	0.023	4.4E-10	2336	66.7	-0.102	0.016	4.1E-10	4338	68.7
Blautia A sp900120195	Blautia A	Lachnospiraceae	Lachnospirales	Clostridia	Bacillota A	-0.156	0.021	9.3E-14	2897	61.4	-0.165	0.052	1.5E-03	561	62.0	-0.154	0.023	4.2E-11	2336	61.3	-0.127	0.017	3.2E-14	4338	62.8
Phascolarctobacterium faecium	Phascolarctobacterium	Acidaminococcaceae	Acidaminococcales	Negativicutes	Bacillota C	0.166	0.022	9.8E-14	2897	44.1	0.176	0.056	1.6E-03	561	49.4	0.159	0.025	2.5E-10	2336	42.9	0.171	0.018	8.5E-21	4338	40.4
Bifidobacterium adolescentis	Bifidobacterium	Bifidobacteriaceae	Actinomycetales	Actinomycetia	Actinomycetota	-0.147	0.020	1.2E-13	2897	77.8	-0.156	0.050	1.8E-03	561	78.8	-0.143	0.022	1.1E-10	2336	77.6	-0.119	0.016	5.1E-14	4338	82.4
Ellagibacter isourolithinifaciens	Ellagibacter	Eggerthellaceae	Coriobacteriales	Coriobacteriia	Actinomycetota	-0.178	0.024	1.7E-13	2897	31.8	-0.124	0.062	4.7E-02	561	28.7	-0.183	0.027	1.1E-11	2336	32.6	-0.168	0.018	3.3E-20	4338	41.8
Blautia A wexlerae B	Blautia A	Lachnospiraceae	Lachnospirales	Clostridia	Bacillota A	-0.170	0.023	1.8E-13	2897	38.4	-0.165	0.058	4.6E-03	561	36.2	-0.171	0.025	2.3E-11	2336	39.0	-0.106	0.018	3.2E-09	4338	47.7
Fimadaptatus sp900553645	Fimadaptatus	CAG-74	Christensenellales	Clostridia	Bacillota A	-0.164	0.023	5.8E-13	2897	44.7	-0.184	0.059	2.0E-03	561	47.1	-0.155	0.025	1.2E-09	2336	44.1	-0.183	0.018	2.0E-23	4338	43.1
Copromonas sp900066785	Copromonas	Lachnospiraceae	Lachnospirales	Clostridia	Bacillota A	0.142	0.020	5.9E-13	2897	79.5	0.179	0.050	3.6E-04	561	80.9	0.132	0.022	2.1E-09	2336	79.1	0.126	0.016	2.3E-15	4338	79.0
Victivallis lenta	Victivallis	Victivallaceae	Victivallales	Lentisphaeria	Verrucomicrobiota	0.165	0.023	6.1E-13	2897	40.2	0.142	0.056	1.2E-02	561	40.5	0.173	0.026	1.9E-11	2336	40.1	0.116	0.019	1.1E-09	4338	35.2
Eisenbergiella tayi	Eisenbergiella	Lachnospiraceae	Lachnospirales	Clostridia	Bacillota A	0.140	0.019	8.9E-13	2897	81.3	0.136	0.052	9.1E-03	561	79.5	0.146	0.022	2.1E-11	2336	81.8	0.136	0.016	1.8E-17	4338	77.8
Streptococcus gordonii	Streptococcus	Streptococcaceae	Lactobacillales	Bacilli	Bacillota	-0.158	0.022	1.0E-12	2897	45.7	-0.135	0.058	2.0E-02	561	44.9	-0.167	0.025	1.4E-11	2336	45.9	-0.138	0.018	2.8E-14	4338	43.3
Anaerotardibacter sp000436075	Anaerotardibacter	Eggerthellaceae	Coriobacteriales	Coriobacteriia	Actinomycetota	-0.154	0.022	1.1E-12	2897	53.0	-0.159	0.057	5.6E-03	561	50.6	-0.152	0.024	3.6E-10	2336	53.6	-0.127	0.017	1.4E-13	4338	60.4
Lentihominibacter faecis	Lentihominibacter	Anaerovoracaceae	Peptostreptococcales	Clostridia	Bacillota A	-0.134	0.019	3.5E-12	2897	93.0	-0.228	0.049	4.8E-06	561	93.2	-0.119	0.021	3.0E-08	2336	92.9	-0.154	0.015	3.4E-23	4338	92.5
Blautia A sp900066355	Blautia A	Lachnospiraceae	Lachnospirales	Clostridia	Bacillota A	-0.134	0.019	4.9E-12	2897	85.1	-0.181	0.049	2.2E-04	561	86.5	-0.121	0.022	2.5E-08	2336	84.8	-0.137	0.016	1.2E-18	4338	86.7
Actinomyces graevenitzii hMGS.05799	Actinomyces	Actinomycetaceae	Actinomycetales	Actinomycetia	Actinomycetota	-0.141	0.020	5.7E-12	2897	66.6	-0.237	0.052	7.1E-06	561	68.8	-0.125	0.023	5.1E-08	2336	66.1	-0.116	0.016	2.7E-12	4338	68.2
Blautia A sp000285855	Blautia A	Lachnospiraceae	Lachnospirales	Clostridia	Bacillota A	-0.138	0.020	6.5E-12	2897	77.4	-0.145	0.050	3.8E-03	561	77.5	-0.133	0.022	2.9E-09	2336	77.4	-0.163	0.016	1.2E-24	4338	80.2
Dysosmobacter sp900542115	Dysosmobacter	Oscillospiraceae	Oscillospirales	Clostridia	Bacillota A	0.135	0.020	8.0E-12	2897	80.1	0.113	0.052	3.0E-02	561	80.7	0.144	0.022	6.6E-11	2336	79.9	0.163	0.016	1.3E-24	4338	77.9

						Women					Premenopausal women					Postmenopausal women					Men				
**Species**	**Genus**	**Family**	**Order**	**Class**	**Phylum**	**β**	**SE**	**P**	**n**	**Prev**	**β**	**SE**	**P**	**n**	**Prev**	**β**	**SE**	**P**	**n**	**Prev**	**β**	**SE**	**P**	**n**	**Prev**
Intestinimonas pullistercoris	Intestinimonas	Oscillospiraceae	Oscillospirales	Clostridia	Bacillota A	0.150	0.022	8.8E-12	2897	47.7	0.168	0.056	2.9E-03	561	47.6	0.145	0.024	3.4E-09	2336	47.7	0.190	0.018	2.9E-25	4338	39.9
AF33-28 sp003477885	AF33-28	Lachnospiraceae	Lachnospirales	Clostridia	Bacillota A	0.134	0.020	1.1E-11	2897	76.9	0.137	0.050	6.2E-03	561	77.2	0.125	0.022	1.5E-08	2336	76.9	0.161	0.016	2.6E-23	4338	71.7
Bacteroides eggerthii	Bacteroides	Bacteroidaceae	Bacteroidales	Bacteroidia	Bacteroidota	0.160	0.024	1.4E-11	2897	34.4	0.227	0.060	1.6E-04	561	34.4	0.150	0.026	1.5E-08	2336	34.5	0.139	0.018	4.9E-14	4338	40.0
Choladousia sp902363665	Choladousia	Lachnospiraceae	Lachnospirales	Clostridia	Bacillota A	-0.134	0.020	1.9E-11	2897	78.6	-0.148	0.050	3.1E-03	561	79.5	-0.135	0.022	1.4E-09	2336	78.4	-0.132	0.016	1.7E-16	4338	78.3
Wujia chipingensis	Wujia	Lachnospiraceae	Lachnospirales	Clostridia	Bacillota A	-0.149	0.022	3.1E-11	2897	44.9	-0.117	0.056	3.9E-02	561	45.1	-0.157	0.025	3.5E-10	2336	44.9	-0.170	0.018	2.5E-21	4338	45.7
Streptococcus mutans	Streptococcus	Streptococcaceae	Lactobacillales	Bacilli	Bacillota	-0.151	0.023	3.3E-11	2897	42.1	-0.156	0.059	8.6E-03	561	39.4	-0.156	0.025	7.1E-10	2336	42.8	-0.111	0.018	1.4E-09	4338	42.8
Merdisoma faecalis	Merdisoma	Lachnospiraceae	Lachnospirales	Clostridia	Bacillota A	0.137	0.021	5.9E-11	2897	61.8	0.141	0.052	7.0E-03	561	59.9	0.133	0.023	1.3E-08	2336	62.3	0.134	0.018	2.1E-14	4338	49.8
Eubacterium I ramulus	Eubacterium I	Lachnospiraceae	Lachnospirales	Clostridia	Bacillota A	-0.127	0.019	8.3E-11	2897	81.9	-0.179	0.050	3.4E-04	561	81.3	-0.116	0.022	1.2E-07	2336	82.1	-0.088	0.016	2.0E-08	4338	82.8
CAG-238 sp900551415	CAG-238	Anaerovoracaceae	Peptostreptococcales	Clostridia	Bacillota A	-0.149	0.023	1.1E-10	2897	40.1	-0.107	0.061	8.2E-02	561	36.0	-0.149	0.026	5.4E-09	2336	41.1	-0.119	0.019	2.4E-10	4338	37.4
JAGTTR01 sp018223385	JAGTTR01	Lachnospiraceae	Lachnospirales	Clostridia	Bacillota A	-0.126	0.020	1.9E-10	2897	80.9	-0.091	0.051	7.5E-02	561	81.5	-0.139	0.022	3.9E-10	2336	80.7	-0.111	0.016	4.4E-12	4338	79.1
Senegalimassilia anaerobia	Senegalimassilia	Eggerthellaceae	Coriobacteriales	Coriobacteriia	Actinomycetota	-0.142	0.022	2.8E-10	2897	45.2	-0.099	0.058	9.2E-02	561	43.9	-0.151	0.025	1.8E-09	2336	45.5	-0.118	0.017	1.1E-11	4338	53.2
Faecousia sp003525905	Faecousia	Oscillospiraceae	Oscillospirales	Clostridia	Bacillota A	0.120	0.019	2.8E-10	2897	90.2	0.108	0.049	3.0E-02	561	90.9	0.126	0.021	4.3E-09	2336	90.1	0.154	0.015	1.3E-23	4338	88.0
Ventricola sp900548125	Ventricola	CAG-74	Christensenellales	Clostridia	Bacillota A	-0.140	0.022	2.9E-10	2897	45.3	-0.137	0.056	1.5E-02	561	45.8	-0.149	0.025	1.9E-09	2336	45.2	-0.165	0.018	8.6E-21	4338	48.5
Eubacterium B sulci	Eubacterium B	Anaerovoracaceae	Peptostreptococcales	Clostridia	Bacillota A	-0.139	0.022	3.4E-10	2897	49.3	-0.238	0.055	1.8E-05	561	51.0	-0.118	0.025	2.0E-06	2336	48.9	-0.124	0.018	4.8E-12	4338	47.3
Eubacterium I sp900546495	Eubacterium I	Lachnospiraceae	Lachnospirales	Clostridia	Bacillota A	-0.145	0.023	4.5E-10	2897	37.2	-0.172	0.058	3.2E-03	561	36.9	-0.141	0.026	6.2E-08	2336	37.2	-0.182	0.018	2.2E-23	4338	40.3
Streptococcus sp001556435	Streptococcus	Streptococcaceae	Lactobacillales	Bacilli	Bacillota	-0.123	0.020	5.2E-10	2897	75.4	-0.169	0.051	9.2E-04	561	75.8	-0.116	0.022	1.9E-07	2336	75.3	-0.114	0.016	2.8E-12	4338	71.4
Anaerostipes hadrus A	Anaerostipes	Lachnospiraceae	Lachnospirales	Clostridia	Bacillota A	-0.134	0.022	5.6E-10	2897	53.4	-0.112	0.055	4.3E-02	561	52.9	-0.142	0.024	3.8E-09	2336	53.6	-0.096	0.017	1.1E-08	4338	61.7
Faecalibacterium prausnitzii	Faecalibacterium	Ruminococcaceae	Oscillospirales	Clostridia	Bacillota A	-0.131	0.021	5.9E-10	2897	56.7	-0.182	0.055	9.9E-04	561	55.8	-0.124	0.024	1.5E-07	2336	57.0	-0.108	0.017	1.1E-10	4338	63.6
Marvinbryantia sp900066075	Marvinbryantia	Lachnospiraceae	Lachnospirales	Clostridia	Bacillota A	-0.137	0.022	6.5E-10	2897	49.6	-0.141	0.055	1.0E-02	561	52.4	-0.131	0.025	1.4E-07	2336	49.0	-0.139	0.017	1.2E-15	4338	53.2
Blautia A wexlerae	Blautia A	Lachnospiraceae	Lachnospirales	Clostridia	Bacillota A	-0.115	0.019	7.0E-10	2897	99.3	-0.131	0.046	4.6E-03	561	99.1	-0.121	0.021	7.1E-09	2336	99.4	-0.095	0.015	2.6E-10	4338	99.4
Streptococcus salivarius	Streptococcus	Streptococcaceae	Lactobacillales	Bacilli	Bacillota	-0.119	0.019	7.8E-10	2897	83.2	-0.191	0.049	1.3E-04	561	84.5	-0.114	0.022	1.6E-07	2336	82.9	-0.126	0.016	1.4E-15	4338	81.7
Fimenecus sp000432435	Fimenecus	Acutalibacteraceae	Oscillospirales	Clostridia	Bacillota A	-0.146	0.024	8.6E-10	2897	35.1	-0.127	0.062	4.3E-02	561	28.9	-0.145	0.026	3.7E-08	2336	36.6	-0.200	0.018	1.3E-27	4338	40.3
Clostridium sp000435835	Clostridium	Clostridiaceae	Clostridiales	Clostridia	Bacillota A	-0.127	0.021	8.7E-10	2897	63.8	-0.180	0.053	7.4E-04	561	65.8	-0.116	0.023	6.1E-07	2336	63.3	-0.115	0.017	6.9E-12	4338	62.3
Anaerotruncus massiliensis	Anaerotruncus	Ruminococcaceae	Oscillospirales	Clostridia	Bacillota A	0.132	0.022	1.1E-09	2897	52.0	0.141	0.054	8.6E-03	561	51.7	0.136	0.024	2.0E-08	2336	52.0	0.134	0.018	7.6E-14	4338	47.0
Pelethomonas sp944387775	Pelethomonas	Oscillospiraceae	Oscillospirales	Clostridia	Bacillota A	0.138	0.023	1.1E-09	2897	42.5	0.210	0.057	2.5E-04	561	41.9	0.128	0.025	5.4E-07	2336	42.7	0.184	0.019	2.2E-22	4338	34.9
Marvinbryantia sp900544685	Marvinbryantia	Lachnospiraceae	Lachnospirales	Clostridia	Bacillota A	-0.139	0.023	2.0E-09	2897	38.6	-0.156	0.060	9.7E-03	561	37.6	-0.133	0.026	2.5E-07	2336	38.8	-0.134	0.018	1.6E-13	4338	42.7
CAG-196 sp002102975	CAG-196	Gastranaerophilaceae	Gastranaerophilales	Vampirovibrionia	Cyanobacteriota	0.133	0.022	2.4E-09	2897	46.5	0.244	0.055	1.3E-05	561	47.1	0.113	0.025	6.6E-06	2336	46.3	0.222	0.018	1.4E-33	4338	40.2
1XD42-69 sp014287635	1XD42-69	Lachnospiraceae	Lachnospirales	Clostridia	Bacillota A	-0.137	0.023	2.5E-09	2897	39.4	-0.147	0.058	1.2E-02	561	43.5	-0.138	0.026	8.9E-08	2336	38.4	-0.152	0.018	2.0E-16	4338	39.7
Faecousia sp900546075	Faecousia	Oscillospiraceae	Oscillospirales	Clostridia	Bacillota A	-0.137	0.023	2.9E-09	2897	38.6	-0.128	0.059	3.2E-02	561	34.6	-0.131	0.026	3.2E-07	2336	39.5	-0.148	0.019	4.0E-15	4338	35.6
Enterocloster pacaense	Enterocloster	Lachnospiraceae	Lachnospirales	Clostridia	Bacillota A	0.142	0.024	2.9E-09	2897	33.3	0.167	0.059	5.1E-03	561	36.5	0.138	0.027	3.0E-07	2336	32.5	0.182	0.019	7.3E-21	4338	30.5
Faecivicinus sp900553265	Faecivicinus	CAG-74	Christensenellales	Clostridia	Bacillota A	-0.128	0.022	3.1E-09	2897	56.4	-0.134	0.055	1.5E-02	561	56.7	-0.122	0.024	5.0E-07	2336	56.3	-0.118	0.017	1.6E-11	4338	53.9
JAJEQR01 sp020687485	JAJEQR01	Lachnospiraceae	Lachnospirales	Clostridia	Bacillota A	-0.121	0.020	3.7E-09	2897	71.7	-0.060	0.051	2.4E-01	561	67.9	-0.136	0.023	2.8E-09	2336	72.6	-0.142	0.016	6.3E-18	4338	68.0
Agathobacter faecis	Agathobacter	Lachnospiraceae	Lachnospirales	Clostridia	Bacillota A	-0.114	0.019	4.7E-09	2897	83.7	-0.107	0.050	3.3E-02	561	80.7	-0.125	0.022	7.6E-09	2336	84.4	-0.048	0.016	2.6E-03	4338	81.8
Clostridium saudiense	Clostridium	Clostridiaceae	Clostridiales	Clostridia	Bacillota A	-0.126	0.022	5.0E-09	2897	52.7	-0.170	0.055	2.0E-03	561	53.5	-0.120	0.024	6.7E-07	2336	52.6	-0.107	0.017	3.6E-10	4338	56.5
Veillonella atypica	Veillonella	Veillonellaceae	Veillonellales	Negativicutes	Bacillota C	-0.131	0.022	5.2E-09	2897	44.4	-0.184	0.056	1.1E-03	561	46.9	-0.119	0.025	2.0E-06	2336	43.8	-0.101	0.018	3.2E-08	4338	43.6
Dysosmobacter sp001916835	Dysosmobacter	Oscillospiraceae	Oscillospirales	Clostridia	Bacillota A	0.114	0.020	5.3E-09	2897	81.1	0.137	0.049	5.4E-03	561	82.5	0.108	0.022	8.7E-07	2336	80.7	0.136	0.016	8.7E-18	4338	81.0
Fournierella excrementavium	Fournierella	Ruminococcaceae	Oscillospirales	Clostridia	Bacillota A	0.122	0.021	6.7E-09	2897	57.4	0.170	0.053	1.5E-03	561	56.0	0.109	0.023	3.2E-06	2336	57.8	0.086	0.018	1.0E-06	4338	51.1
Choladocola sp003480725	Choladocola	Lachnospiraceae	Lachnospirales	Clostridia	Bacillota A	0.111	0.019	8.0E-09	2897	91.6	0.142	0.050	4.6E-03	561	92.9	0.099	0.021	3.5E-06	2336	91.4	0.142	0.015	1.7E-20	4338	92.5
Eggerthella lenta	Eggerthella	Eggerthellaceae	Coriobacteriales	Coriobacteriia	Actinomycetota	0.114	0.020	1.0E-08	2897	78.4	0.074	0.051	1.5E-01	561	78.8	0.119	0.022	8.8E-08	2336	78.3	0.122	0.016	9.1E-14	4338	69.8
Ruminococcus C sp000433635	Ruminococcus C	Ruminococcaceae	Oscillospirales	Clostridia	Bacillota A	-0.137	0.024	1.4E-08	2897	31.3	-0.094	0.062	1.3E-01	561	33.7	-0.149	0.027	4.4E-08	2336	30.7	-0.113	0.019	2.0E-09	4338	37.4
Intestinibacter bartlettii	Intestinibacter	Peptostreptococcaceae	Peptostreptococcales	Clostridia	Bacillota A	-0.112	0.020	1.6E-08	2897	78.1	-0.168	0.050	8.4E-04	561	80.2	-0.104	0.022	2.8E-06	2336	77.6	-0.077	0.016	1.3E-06	4338	80.7
Acetatifactor sp900066565	Acetatifactor	Lachnospiraceae	Lachnospirales	Clostridia	Bacillota A	0.106	0.019	1.7E-08	2897	96.2	0.026	0.047	5.7E-01	561	96.4	0.114	0.021	6.7E-08	2336	96.2	0.143	0.015	2.0E-21	4338	96.8
Enterocloster asparagiformis	Enterocloster	Lachnospiraceae	Lachnospirales	Clostridia	Bacillota A	0.133	0.024	1.9E-08	2897	34.4	0.099	0.060	1.0E-01	561	33.2	0.135	0.026	3.8E-07	2336	34.7	0.140	0.019	9.6E-14	4338	36.3
HGM12998 sp900756495	HGM12998	Oscillospiraceae	Oscillospirales	Clostridia	Bacillota A	0.110	0.019	2.0E-08	2897	84.7	0.072	0.049	1.4E-01	561	83.8	0.118	0.022	7.0E-08	2336	84.9	0.160	0.016	1.0E-23	4338	79.3
Dorea A longicatena B	Dorea A	Lachnospiraceae	Lachnospirales	Clostridia	Bacillota A	-0.114	0.020	2.0E-08	2897	71.8	-0.178	0.050	4.4E-04	561	72.7	-0.102	0.023	6.8E-06	2336	71.6	-0.098	0.016	1.5E-09	4338	74.1
Granulicatella hMGS.06388	Granulicatella	Aerococcaceae	Lactobacillales	Bacilli	Bacillota	-0.125	0.022	2.0E-08	2897	46.7	-0.160	0.058	5.9E-03	561	46.0	-0.118	0.025	2.1E-06	2336	46.9	-0.110	0.018	8.4E-10	4338	47.3
Blautia A sp900551715	Blautia A	Lachnospiraceae	Lachnospirales	Clostridia	Bacillota A	-0.135	0.024	2.5E-08	2897	30.9	-0.123	0.064	5.5E-02	561	27.5	-0.138	0.027	3.0E-07	2336	31.7	-0.170	0.019	1.0E-18	4338	32.1
Alistipes A pullistercoris	Alistipes A	Rikenellaceae	Bacteroidales	Bacteroidia	Bacteroidota	0.120	0.022	3.8E-08	2897	51.0	0.145	0.055	8.6E-03	561	51.2	0.118	0.025	1.5E-06	2336	50.9	0.158	0.018	3.1E-19	4338	49.9
UBA644 sp900547165	UBA644	UBA644	Oscillospirales	Clostridia	Bacillota A	-0.125	0.023	4.8E-08	2897	40.2	-0.151	0.057	8.3E-03	561	40.5	-0.121	0.026	2.6E-06	2336	40.2	-0.093	0.019	6.1E-07	4338	38.4
Alistipes senegalensis	Alistipes	Rikenellaceae	Bacteroidales	Bacteroidia	Bacteroidota	0.119	0.022	4.8E-08	2897	53.8	0.205	0.054	1.8E-04	561	52.9	0.109	0.024	7.8E-06	2336	54.0	0.097	0.017	1.7E-08	4338	57.3
Gehongia tenuis	Gehongia	NSJ-53	Christensenellales	Clostridia	Bacillota A	-0.117	0.021	4.9E-08	2897	57.6	-0.153	0.055	5.7E-03	561	59.5	-0.108	0.024	6.3E-06	2336	57.2	-0.084	0.017	1.6E-06	4338	54.0
CAG-170 sp905214155	CAG-170	Oscillospiraceae	Oscillospirales	Clostridia	Bacillota A	-0.124	0.023	5.0E-08	2897	43.5	-0.038	0.058	5.1E-01	561	43.3	-0.145	0.025	1.4E-08	2336	43.5	-0.145	0.018	2.6E-15	4338	41.9
Turicibacter bilis	Turicibacter	Turicibacteraceae	Haloplasmatales	Bacilli	Bacillota	-0.112	0.021	5.2E-08	2897	65.5	-0.108	0.052	4.0E-02	561	68.6	-0.117	0.023	4.2E-07	2336	64.8	-0.106	0.017	1.7E-10	4338	65.6
Massilimicrobiota sp002160865	Massilimicrobiota	Coprobacillaceae	Erysipelotrichales	Bacilli	Bacillota	0.128	0.023	5.9E-08	2897	35.5	0.170	0.057	2.8E-03	561	37.6	0.120	0.026	5.7E-06	2336	35.0	0.134	0.019	9.1E-13	4338	37.3
Blautia A sp900763175	Blautia A	Lachnospiraceae	Lachnospirales	Clostridia	Bacillota A	-0.126	0.023	6.4E-08	2897	36.6	-0.141	0.061	2.1E-02	561	34.6	-0.123	0.026	2.6E-06	2336	37.1	-0.135	0.018	1.7E-13	4338	40.3
CAG-95 sp000438155	CAG-95	Lachnospiraceae	Lachnospirales	Clostridia	Bacillota A	-0.110	0.021	7.9E-08	2897	69.3	-0.145	0.051	5.0E-03	561	71.7	-0.101	0.023	1.2E-05	2336	68.8	-0.118	0.017	1.6E-12	4338	66.4
Agathobaculum butyriciproducens	Agathobaculum	Butyricicoccaceae	Oscillospirales	Clostridia	Bacillota A	-0.100	0.019	8.5E-08	2897	98.2	-0.165	0.048	5.5E-04	561	98.4	-0.094	0.021	7.2E-06	2336	98.2	-0.060	0.015	6.8E-05	4338	97.9
CAG-103 sp000432375	CAG-103	Oscillospiraceae	Oscillospirales	Clostridia	Bacillota A	-0.117	0.022	9.1E-08	2897	48.4	-0.112	0.055	4.2E-02	561	51.2	-0.123	0.025	5.7E-07	2336	47.8	-0.075	0.017	1.5E-05	4338	50.6

						Women					Premenopausal women					Postmenopausal women					Men				
**Species**	**Genus**	**Family**	**Order**	**Class**	**Phylum**	**β**	**SE**	**P**	**n**	**Prev**	**β**	**SE**	**P**	**n**	**Prev**	**β**	**SE**	**P**	**n**	**Prev**	**β**	**SE**	**P**	**n**	**Prev**
UBA3402 sp003478355	UBA3402	Lachnospiraceae	Lachnospirales	Clostridia	Bacillota A	0.104	0.020	1.3E-07	2897	81.8	0.161	0.050	1.3E-03	561	83.8	0.097	0.022	9.9E-06	2336	81.3	0.098	0.016	8.1E-10	4338	80.0
UMGS1766 sp900554855	UMGS1766	Oscillospiraceae	Oscillospirales	Clostridia	Bacillota A	-0.122	0.023	1.7E-07	2897	38.8	-0.185	0.060	2.3E-03	561	36.7	-0.111	0.026	1.9E-05	2336	39.3	-0.158	0.018	8.1E-18	4338	40.2
Clostridium sp900540255	Clostridium	Clostridiaceae	Clostridiales	Clostridia	Bacillota A	-0.109	0.021	1.9E-07	2897	60.1	-0.092	0.053	8.1E-02	561	59.5	-0.112	0.023	1.7E-06	2336	60.2	-0.115	0.017	2.7E-11	4338	54.5
Mediterraneibacter torques	Mediterraneibacter	Lachnospiraceae	Lachnospirales	Clostridia	Bacillota A	0.103	0.020	2.0E-07	2897	79.4	0.056	0.050	2.6E-01	561	75.9	0.109	0.022	6.4E-07	2336	80.2	0.129	0.016	2.1E-16	4338	80.4
Intestinibacter sp900540355	Intestinibacter	Peptostreptococcaceae	Peptostreptococcales	Clostridia	Bacillota A	-0.111	0.021	2.1E-07	2897	56.2	-0.141	0.054	9.5E-03	561	54.5	-0.107	0.024	7.2E-06	2336	56.6	-0.128	0.017	9.8E-14	4338	56.4
Egerieenecus sp900553315	Egerieenecus	CAG-74	Christensenellales	Clostridia	Bacillota A	-0.117	0.023	2.3E-07	2897	43.9	-0.161	0.058	5.8E-03	561	41.4	-0.105	0.025	2.9E-05	2336	44.5	-0.121	0.018	5.2E-11	4338	41.8
Dysosmobacter pullicola	Dysosmobacter	Oscillospiraceae	Oscillospirales	Clostridia	Bacillota A	0.120	0.023	2.4E-07	2897	38.7	0.106	0.059	7.4E-02	561	37.6	0.121	0.026	3.3E-06	2336	39.0	0.151	0.019	5.9E-16	4338	37.9
Bittarella massiliensis	Bittarella	Ruminococcaceae	Oscillospirales	Clostridia	Bacillota A	-0.097	0.019	2.6E-07	2897	95.5	-0.166	0.047	4.5E-04	561	96.1	-0.088	0.021	3.0E-05	2336	95.3	-0.070	0.015	3.5E-06	4338	96.0
HGM11808 sp900757025	HGM11808	Anaerotignaceae	Lachnospirales	Clostridia	Bacillota A	-0.120	0.024	3.9E-07	2897	34.6	-0.095	0.060	1.1E-01	561	35.5	-0.123	0.026	3.3E-06	2336	34.4	-0.081	0.018	1.0E-05	4338	41.8
Dysosmobacter sp900550685	Dysosmobacter	Oscillospiraceae	Oscillospirales	Clostridia	Bacillota A	-0.121	0.024	5.4E-07	2897	31.8	-0.058	0.061	3.4E-01	561	32.6	-0.133	0.027	7.0E-07	2336	31.5	-0.159	0.019	4.5E-17	4338	34.3
Streptococcus parasanguinis	Streptococcus	Streptococcaceae	Lactobacillales	Bacilli	Bacillota	-0.112	0.022	5.8E-07	2897	43.7	-0.130	0.058	2.4E-02	561	43.5	-0.112	0.025	8.7E-06	2336	43.8	-0.134	0.018	2.2E-13	4338	42.1
Pelethomonas sp900549475	Pelethomonas	Oscillospiraceae	Oscillospirales	Clostridia	Bacillota A	0.109	0.022	6.6E-07	2897	48.3	0.146	0.054	7.2E-03	561	48.7	0.102	0.025	3.7E-05	2336	48.2	0.174	0.018	2.2E-21	4338	40.2
Clostridium AQ innocuum	Clostridium AQ	Erysipelotrichaceae	Erysipelotrichales	Bacilli	Bacillota	0.103	0.021	6.8E-07	2897	64.8	0.057	0.053	2.8E-01	561	65.2	0.116	0.023	6.4E-07	2336	64.7	0.097	0.017	5.4E-09	4338	66.6
Gallacutalibacter pullicola	Gallacutalibacter	Acutalibacteraceae	Oscillospirales	Clostridia	Bacillota A	0.109	0.022	7.3E-07	2897	47.8	0.172	0.055	1.9E-03	561	46.9	0.098	0.025	7.2E-05	2336	48.0	0.131	0.018	1.9E-13	4338	45.5
Coprobacter secundus	Coprobacter	Coprobacteraceae	Bacteroidales	Bacteroidia	Bacteroidota	0.117	0.024	7.8E-07	2897	34.6	0.105	0.060	8.2E-02	561	37.1	0.128	0.027	1.4E-06	2336	33.9	0.162	0.019	7.4E-18	4338	35.7
Lentihominibacter sp900066305	Lentihominibacter	Anaerovoracaceae	Peptostreptococcales	Clostridia	Bacillota A	-0.098	0.020	8.7E-07	2897	82.8	-0.146	0.052	5.2E-03	561	83.4	-0.092	0.022	3.4E-05	2336	82.6	-0.105	0.016	2.9E-11	4338	83.1
Phocaeicola massiliensis	Phocaeicola	Bacteroidaceae	Bacteroidales	Bacteroidia	Bacteroidota	0.117	0.024	9.0E-07	2897	34.0	0.129	0.061	3.4E-02	561	34.4	0.115	0.027	1.4E-05	2336	33.9	0.208	0.018	4.7E-29	4338	38.0
Lactococcus cremoris	Lactococcus	Streptococcaceae	Lactobacillales	Bacilli	Bacillota	-0.110	0.022	9.3E-07	2897	44.1	-0.177	0.056	1.8E-03	561	44.9	-0.094	0.025	1.8E-04	2336	43.9	-0.107	0.019	1.2E-08	4338	37.1
UBA1417 sp003531055	UBA1417	Acutalibacteraceae	Oscillospirales	Clostridia	Bacillota A	-0.093	0.019	9.9E-07	2897	91.3	-0.136	0.049	5.5E-03	561	91.8	-0.087	0.021	5.1E-05	2336	91.2	-0.085	0.015	3.3E-08	4338	91.7
Veillonella parvula hMGS.05759	Veillonella	Veillonellaceae	Veillonellales	Negativicutes	Bacillota C	-0.114	0.023	1.0E-06	2897	36.5	-0.136	0.057	1.8E-02	561	40.1	-0.104	0.026	7.7E-05	2336	35.6	-0.094	0.019	5.1E-07	4338	38.2
Gemmiger sp900540775	Gemmiger	Ruminococcaceae	Oscillospirales	Clostridia	Bacillota A	-0.104	0.021	1.2E-06	2897	57.3	-0.098	0.054	6.9E-02	561	56.7	-0.102	0.024	1.8E-05	2336	57.4	-0.077	0.017	3.7E-06	4338	62.5
Copromonas sp900066535	Copromonas	Lachnospiraceae	Lachnospirales	Clostridia	Bacillota A	0.093	0.019	1.4E-06	2897	91.3	0.168	0.048	5.2E-04	561	93.8	0.081	0.022	1.9E-04	2336	90.7	0.136	0.015	6.3E-19	4338	92.1
CAG-95 sp900066375	CAG-95	Lachnospiraceae	Lachnospirales	Clostridia	Bacillota A	-0.098	0.020	1.4E-06	2897	71.2	-0.116	0.051	2.4E-02	561	70.6	-0.085	0.023	2.0E-04	2336	71.3	-0.127	0.016	1.5E-14	4338	69.5
Negativibacillus faecipullorum	Negativibacillus	Ruminococcaceae	Oscillospirales	Clostridia	Bacillota A	0.106	0.022	1.5E-06	2897	47.6	0.174	0.055	1.6E-03	561	48.8	0.089	0.025	3.4E-04	2336	47.3	0.131	0.018	4.7E-13	4338	42.0
Dysosmobacter sp900544615	Dysosmobacter	Oscillospiraceae	Oscillospirales	Clostridia	Bacillota A	-0.109	0.023	1.5E-06	2897	43.3	-0.061	0.058	2.9E-01	561	42.8	-0.118	0.025	3.4E-06	2336	43.4	-0.140	0.018	7.9E-15	4338	44.6
Ventrisoma faecale	Ventrisoma	Lachnospiraceae	Lachnospirales	Clostridia	Bacillota A	0.101	0.021	1.7E-06	2897	59.8	0.054	0.054	3.2E-01	561	62.6	0.110	0.023	2.7E-06	2336	59.2	0.099	0.017	5.5E-09	4338	58.1
Avoscillospira A sp019422005	Avoscillospira A	Oscillospiraceae	Oscillospirales	Clostridia	Bacillota A	0.102	0.021	2.1E-06	2897	53.8	0.124	0.054	2.2E-02	561	54.0	0.095	0.024	9.0E-05	2336	53.8	0.114	0.018	8.4E-11	4338	50.0
Blautia A sp934364795	Blautia A	Lachnospiraceae	Lachnospirales	Clostridia	Bacillota A	-0.114	0.024	2.4E-06	2897	31.0	-0.066	0.062	2.9E-01	561	31.6	-0.117	0.027	1.4E-05	2336	30.9	-0.069	0.019	3.5E-04	4338	32.2
Roseburia intestinalis	Roseburia	Lachnospiraceae	Lachnospirales	Clostridia	Bacillota A	-0.092	0.019	2.5E-06	2897	85.6	-0.065	0.051	2.0E-01	561	83.8	-0.103	0.022	2.2E-06	2336	86.0	-0.021	0.016	1.8E-01	4338	84.3
Pauljensenia sp916439125	Pauljensenia	Actinomycetaceae	Actinomycetales	Actinomycetia	Actinomycetota	-0.107	0.023	2.5E-06	2897	40.1	-0.192	0.057	8.1E-04	561	39.9	-0.100	0.025	9.4E-05	2336	40.2	-0.081	0.018	9.8E-06	4338	40.9
Faecousia sp900544705	Faecousia	Oscillospiraceae	Oscillospirales	Clostridia	Bacillota A	-0.108	0.023	3.5E-06	2897	37.8	-0.118	0.060	5.0E-02	561	35.3	-0.109	0.026	2.8E-05	2336	38.4	-0.146	0.019	3.4E-14	4338	33.0
Phocea massiliensis	Phocea	Ruminococcaceae	Oscillospirales	Clostridia	Bacillota A	0.094	0.020	4.0E-06	2897	69.4	0.095	0.053	7.1E-02	561	68.1	0.093	0.023	4.7E-05	2336	69.7	0.143	0.016	3.9E-19	4338	74.9
Leuconostoc mesenteroides	Leuconostoc	Lactobacillaceae	Lactobacillales	Bacilli	Bacillota	-0.101	0.022	4.6E-06	2897	49.2	-0.201	0.055	2.9E-04	561	48.0	-0.089	0.025	2.9E-04	2336	49.5	-0.108	0.018	1.9E-09	4338	45.7
Faecalibacterium prausnitzii I	Faecalibacterium	Ruminococcaceae	Oscillospirales	Clostridia	Bacillota A	-0.090	0.020	6.1E-06	2897	77.7	-0.132	0.050	8.1E-03	561	81.1	-0.079	0.022	3.8E-04	2336	76.9	-0.057	0.016	3.8E-04	4338	76.3
Faecalibacterium sp900539945	Faecalibacterium	Ruminococcaceae	Oscillospirales	Clostridia	Bacillota A	-0.087	0.019	6.3E-06	2897	85.6	-0.039	0.049	4.2E-01	561	87.3	-0.097	0.022	8.4E-06	2336	85.2	-0.045	0.016	3.7E-03	4338	86.4
Marvinbryantia shaoxingensis	Marvinbryantia	Lachnospiraceae	Lachnospirales	Clostridia	Bacillota A	-0.106	0.023	6.3E-06	2897	37.6	-0.048	0.060	4.3E-01	561	37.1	-0.114	0.026	1.3E-05	2336	37.8	-0.086	0.018	2.6E-06	4338	42.3
RUG11247 sp902767315	RUG11247	CAG-74	Christensenellales	Clostridia	Bacillota A	-0.102	0.023	6.7E-06	2897	45.8	-0.112	0.056	4.6E-02	561	48.1	-0.093	0.025	2.6E-04	2336	45.2	-0.115	0.018	4.7E-10	4338	41.8
Schaedlerella sp900066545	Schaedlerella	Lachnospiraceae	Lachnospirales	Clostridia	Bacillota A	-0.104	0.023	6.7E-06	2897	39.2	-0.086	0.057	1.3E-01	561	43.7	-0.109	0.026	2.5E-05	2336	38.2	-0.148	0.018	1.2E-16	4338	46.4
Terrisporobacter sp900557165	Terrisporobacter	Peptostreptococcaceae	Peptostreptococcales	Clostridia	Bacillota A	-0.096	0.021	7.0E-06	2897	54.8	-0.083	0.056	1.4E-01	561	50.8	-0.103	0.024	1.5E-05	2336	55.8	-0.088	0.017	3.3E-07	4338	56.2
Collinsella aerofaciens hMGS.05755	Collinsella	Coriobacteriaceae	Coriobacteriales	Coriobacteriia	Actinomycetota	-0.099	0.022	7.2E-06	2897	48.7	-0.071	0.055	2.0E-01	561	47.4	-0.107	0.025	1.5E-05	2336	49.1	-0.079	0.017	5.1E-06	4338	53.5
UBA738 sp003522945	UBA738	Oscillospiraceae	Oscillospirales	Clostridia	Bacillota A	-0.100	0.022	8.0E-06	2897	47.6	-0.045	0.057	4.3E-01	561	43.3	-0.110	0.025	1.0E-05	2336	48.6	-0.115	0.018	1.5E-10	4338	45.7
Scatomorpha sp900545405	Scatomorpha	Oscillospiraceae	Oscillospirales	Clostridia	Bacillota A	0.092	0.021	8.2E-06	2897	63.7	0.073	0.053	1.7E-01	561	64.5	0.101	0.023	1.2E-05	2336	63.4	0.083	0.017	1.1E-06	4338	57.9
Ventricola sp900542445	Ventricola	CAG-74	Christensenellales	Clostridia	Bacillota A	-0.089	0.020	8.4E-06	2897	75.1	-0.170	0.051	8.6E-04	561	76.6	-0.072	0.022	1.3E-03	2336	74.7	-0.062	0.016	1.3E-04	4338	73.8
Gemmiger sp900539695	Gemmiger	Ruminococcaceae	Oscillospirales	Clostridia	Bacillota A	-0.088	0.020	8.4E-06	2897	80.4	-0.138	0.048	4.8E-03	561	82.7	-0.080	0.022	3.0E-04	2336	79.9	-0.088	0.016	3.1E-08	4338	81.3
Bacteroides faecis	Bacteroides	Bacteroidaceae	Bacteroidales	Bacteroidia	Bacteroidota	0.108	0.024	1.1E-05	2897	30.5	0.083	0.062	1.8E-01	561	28.5	0.121	0.027	9.9E-06	2336	31.0	0.173	0.019	2.0E-19	4338	34.1
Merdisoma sp014290175	Merdisoma	Lachnospiraceae	Lachnospirales	Clostridia	Bacillota A	-0.089	0.020	1.2E-05	2897	69.5	-0.115	0.052	2.8E-02	561	72.0	-0.083	0.023	2.5E-04	2336	68.9	-0.073	0.016	6.8E-06	4338	70.5
Avimonas sp900551425	Avimonas	Acutalibacteraceae	Oscillospirales	Clostridia	Bacillota A	0.097	0.022	1.3E-05	2897	45.0	0.027	0.057	6.3E-01	561	46.7	0.118	0.025	2.5E-06	2336	44.6	0.151	0.018	2.6E-16	4338	39.6
Bittarella massiliensis A	Bittarella	Ruminococcaceae	Oscillospirales	Clostridia	Bacillota A	-0.094	0.022	1.6E-05	2897	51.2	-0.195	0.055	4.3E-04	561	47.6	-0.073	0.024	2.5E-03	2336	52.1	-0.101	0.017	8.9E-09	4338	51.0
JAHZIE01 sp019419895	JAHZIE01	Acutalibacteraceae	Oscillospirales	Clostridia	Bacillota A	0.100	0.023	1.6E-05	2897	37.7	0.048	0.059	4.2E-01	561	38.3	0.110	0.026	2.5E-05	2336	37.5	0.103	0.018	4.8E-09	4338	49.9
Eisenbergiella porci	Eisenbergiella	Lachnospiraceae	Lachnospirales	Clostridia	Bacillota A	0.093	0.022	1.7E-05	2897	52.4	0.026	0.055	6.3E-01	561	50.4	0.113	0.024	2.9E-06	2336	52.8	0.130	0.018	2.2E-13	4338	48.3
Ventricola sp900542395	Ventricola	CAG-74	Christensenellales	Clostridia	Bacillota A	-0.094	0.022	1.9E-05	2897	51.2	-0.047	0.056	4.1E-01	561	52.8	-0.110	0.025	7.7E-06	2336	50.8	-0.143	0.017	3.2E-16	4338	52.1
Dysosmobacter avistercoris	Dysosmobacter	Oscillospiraceae	Oscillospirales	Clostridia	Bacillota A	0.091	0.021	1.9E-05	2897	56.4	0.105	0.054	5.5E-02	561	54.0	0.090	0.024	1.6E-04	2336	56.9	0.158	0.017	4.6E-20	4338	53.7
Enterocloster sp005845215	Enterocloster	Lachnospiraceae	Lachnospirales	Clostridia	Bacillota A	0.098	0.023	2.0E-05	2897	39.2	-0.018	0.060	7.6E-01	561	37.3	0.130	0.026	4.1E-07	2336	39.6	0.128	0.019	3.0E-11	4338	33.2
Copromonas sp000435795	Copromonas	Lachnospiraceae	Lachnospirales	Clostridia	Bacillota A	0.089	0.021	2.2E-05	2897	61.8	0.047	0.053	3.8E-01	561	65.6	0.094	0.023	5.4E-05	2336	60.9	0.150	0.016	7.6E-20	4338	66.1
Lachnospira eligens A	Lachnospira	Lachnospiraceae	Lachnospirales	Clostridia	Bacillota A	0.083	0.020	2.8E-05	2897	80.3	0.063	0.050	2.1E-01	561	80.7	0.087	0.022	7.6E-05	2336	80.2	0.120	0.016	3.7E-14	4338	78.9
Feifania hominis	Feifania	Feifaniaceae	Oscillospirales	Clostridia	Bacillota A	0.088	0.021	2.8E-05	2897	60.6	0.052	0.054	3.3E-01	561	59.4	0.093	0.024	7.8E-05	2336	61.0	0.102	0.017	5.3E-09	4338	53.8
JAHZGC01 sp019421015	JAHZGC01	UMGS124	Coriobacteriales	Coriobacteriia	Actinomycetota	-0.088	0.021	3.0E-05	2897	60.3	-0.105	0.054	5.2E-02	561	59.0	-0.081	0.024	5.9E-04	2336	60.7	-0.020	0.017	2.3E-01	4338	61.6
Rothia mucilaginosa A	Rothia	Micrococcaceae	Actinomycetales	Actinomycetia	Actinomycetota	-0.100	0.024	3.0E-05	2897	33.1	-0.110	0.064	8.4E-02	561	29.8	-0.092	0.027	5.6E-04	2336	33.9	-0.117	0.019	1.4E-09	4338	32.5

						Women					Premenopausal women					Postmenopausal women					Men				
Species	Genus	Family	Order	Class	Phylum	β	SE	P	n	Prev	β	SE	P	n	Prev	β	SE	P	n	Prev	β	SE	P	n	Prev
Faecalibacterium sp900758465	Faecalibacterium	Ruminococcaceae	Oscillospirales	Clostridia	Bacillota A	0.082	0.020	3.1E-05	2897	80.8	0.068	0.049	1.7E-01	561	81.8	0.082	0.022	1.9E-04	2336	80.5	0.052	0.016	9.6E-04	4338	80.5
Acetatifactor sp900755865	Acetatifactor	Lachnospiraceae	Lachnospirales	Clostridia	Bacillota A	-0.097	0.023	3.2E-05	2897	38.8	-0.081	0.059	1.7E-01	561	42.1	-0.106	0.026	4.9E-05	2336	38.1	-0.090	0.019	1.7E-06	4338	36.5
Lachnospira sp000437735	Lachnospira	Lachnospiraceae	Lachnospirales	Clostridia	Bacillota A	0.082	0.020	3.3E-05	2897	78.7	0.102	0.051	4.5E-02	561	79.0	0.076	0.022	5.7E-04	2336	78.6	0.097	0.016	1.2E-09	4338	77.4
Lactococcus lactis	Lactococcus	Streptococcaceae	Lactobacillales	Bacilli	Bacillota	-0.087	0.021	3.4E-05	2897	60.8	-0.077	0.054	1.5E-01	561	61.9	-0.095	0.023	5.4E-05	2336	60.5	-0.066	0.017	1.5E-04	4338	52.0
Lachnospira sp900316325	Lachnospira	Lachnospiraceae	Lachnospirales	Clostridia	Bacillota A	0.086	0.021	3.5E-05	2897	64.3	0.066	0.053	2.1E-01	561	65.6	0.093	0.023	6.0E-05	2336	64.0	0.089	0.017	1.4E-07	4338	59.3
Ventrimonas sp003480315	Ventrimonas	Lachnospiraceae	Lachnospirales	Clostridia	Bacillota A	0.081	0.020	3.6E-05	2897	81.9	0.134	0.050	7.3E-03	561	82.2	0.068	0.022	1.9E-03	2336	81.8	0.089	0.016	1.8E-08	4338	81.4
UBA737 sp900549055	UBA737	Acutalibacteraceae	Oscillospirales	Clostridia	Bacillota A	-0.088	0.021	3.7E-05	2897	57.5	-0.060	0.054	2.6E-01	561	57.4	-0.093	0.024	9.7E-05	2336	57.5	-0.101	0.017	4.1E-09	4338	55.0
Marvinbryantia sp900550755	Marvinbryantia	Lachnospiraceae	Lachnospirales	Clostridia	Bacillota A	0.093	0.023	4.5E-05	2897	40.3	-0.004	0.059	9.5E-01	561	37.8	0.119	0.025	2.9E-06	2336	40.9	0.076	0.020	1.0E-04	4338	30.8
Woodwardibium sp900760275	Woodwardibium	CAG-382	Oscillospirales	Clostridia	Bacillota A	0.092	0.023	5.3E-05	2897	41.8	0.041	0.057	4.7E-01	561	43.0	0.098	0.025	1.2E-04	2336	41.5	0.106	0.019	2.8E-08	4338	34.1
Spyradocola merdavium	Spyradocola	UBA1750	Christensenellales	Clostridia	Bacillota A	0.088	0.022	5.3E-05	2897	51.7	0.114	0.054	3.6E-02	561	48.7	0.083	0.024	6.8E-04	2336	52.4	0.110	0.018	5.9E-10	4338	46.9
Blautia A massiliensis	Blautia A	Lachnospiraceae	Lachnospirales	Clostridia	Bacillota A	-0.077	0.019	5.4E-05	2897	92.1	-0.115	0.050	2.1E-02	561	92.0	-0.077	0.021	2.8E-04	2336	92.2	-0.075	0.015	9.2E-07	4338	94.1
Faecousia sp000434635	Faecousia	Oscillospiraceae	Oscillospirales	Clostridia	Bacillota A	-0.084	0.021	5.5E-05	2897	64.5	-0.070	0.053	1.8E-01	561	64.2	-0.083	0.023	3.5E-04	2336	64.6	-0.086	0.017	3.4E-07	4338	62.1
Bacteroides stercoris	Bacteroides	Bacteroidaceae	Bacteroidales	Bacteroidia	Bacteroidota	0.092	0.023	6.0E-05	2897	40.1	0.140	0.059	1.7E-02	561	39.9	0.089	0.025	4.8E-04	2336	40.1	0.215	0.017	2.7E-34	4338	47.6
Limiplasma sp900548145	Limiplasma	CAG-74	Christensenellales	Clostridia	Bacillota A	-0.090	0.022	6.3E-05	2897	46.4	-0.178	0.058	2.3E-03	561	48.0	-0.071	0.025	4.9E-03	2336	46.1	-0.132	0.018	5.0E-13	4338	44.0
Pseudoscilispira sp020555605	Pseudoscilispira	Oscillospiraceae	Oscillospirales	Clostridia	Bacillota A	0.093	0.023	6.6E-05	2897	37.5	0.119	0.059	4.5E-02	561	39.9	0.093	0.026	3.8E-04	2336	36.9	0.136	0.019	3.8E-13	4338	37.0
Clostridium sp001916075	Clostridium	Clostridiaceae	Clostridiales	Clostridia	Bacillota A	-0.087	0.022	7.1E-05	2897	48.3	-0.111	0.055	4.6E-02	561	53.1	-0.072	0.025	3.8E-03	2336	47.1	-0.076	0.018	2.4E-05	4338	46.4
UMGS1484 sp900552285	UMGS1484	Acutalibacteraceae	Oscillospirales	Clostridia	Bacillota A	-0.096	0.024	7.3E-05	2897	30.8	-0.090	0.063	1.5E-01	561	30.7	-0.094	0.027	5.8E-04	2336	30.9	-0.128	0.019	5.1E-11	4338	31.7
UMGS1071 sp900542375	UMGS1071	Acutalibacteraceae	Oscillospirales	Clostridia	Bacillota A	0.083	0.021	7.9E-05	2897	58.7	0.020	0.055	7.1E-01	561	57.9	0.097	0.023	3.9E-05	2336	58.9	0.105	0.017	2.0E-09	4338	51.7
UMGS973 sp900547295	UMGS973	Anaerovoracaceae	Peptostreptococcales	Clostridia	Bacillota A	-0.093	0.024	8.3E-05	2897	38.2	-0.181	0.061	3.1E-03	561	37.8	-0.075	0.026	4.5E-03	2336	38.3	-0.123	0.019	9.7E-11	4338	35.6
Hydrogeniiclostridium mannosilyticum	Hydrogeniiclostridium	Acutalibacteraceae	Oscillospirales	Clostridia	Bacillota A	0.083	0.021	8.5E-05	2897	58.6	-0.023	0.053	6.6E-01	561	59.2	0.105	0.023	7.3E-06	2336	58.5	0.098	0.017	5.3E-09	4338	61.0
Enterocloster excrementigallinarum	Enterocloster	Lachnospiraceae	Lachnospirales	Clostridia	Bacillota A	0.082	0.021	8.6E-05	2897	60.7	0.133	0.053	1.3E-02	561	63.1	0.074	0.024	1.7E-03	2336	60.1	0.078	0.017	5.4E-06	4338	55.4
Faecalibacterium duncaniae	Faecalibacterium	Ruminococcaceae	Oscillospirales	Clostridia	Bacillota A	-0.075	0.019	8.6E-05	2897	90.2	-0.028	0.048	5.6E-01	561	92.0	-0.086	0.021	6.8E-05	2336	89.8	-0.053	0.015	6.2E-04	4338	90.9
Vescimonas sp900548615	Vescimonas	Oscillospiraceae	Oscillospirales	Clostridia	Bacillota A	-0.091	0.023	8.7E-05	2897	38.8	-0.083	0.061	1.7E-01	561	36.0	-0.084	0.026	1.2E-03	2336	39.4	-0.066	0.019	5.1E-04	4338	36.3
CAG-170 sp900545925	CAG-170	Oscillospiraceae	Oscillospirales	Clostridia	Bacillota A	-0.081	0.021	9.1E-05	2897	65.0	-0.061	0.053	2.5E-01	561	66.5	-0.089	0.023	1.4E-04	2336	64.6	-0.020	0.017	2.4E-01	4338	61.0
CAG-313 sp000433035	CAG-313	CAG-313	ML615J-28	Bacilli	Bacillota	0.091	0.023	9.9E-05	2897	36.0	0.052	0.059	3.8E-01	561	35.8	0.102	0.026	1.1E-04	2336	36.0	0.137	0.020	3.0E-12	4338	30.0
Adlercreutzia hattorii	Adlercreutzia	Eggerthellaceae	Coriobacteriales	Coriobacteriia	Actinomycetota	0.077	0.020	1.1E-04	2897	80.8	0.059	0.050	2.4E-01	561	79.1	0.086	0.022	9.1E-05	2336	81.2	0.116	0.016	3.3E-13	4338	80.6
Clostridium Q saccharolyticum A	Clostridium Q	Lachnospiraceae	Lachnospirales	Clostridia	Bacillota A	0.091	0.023	1.1E-04	2897	37.1	0.121	0.058	3.7E-02	561	39.2	0.086	0.026	1.2E-03	2336	36.6	0.109	0.018	3.0E-09	4338	40.3
Lachnoclostridium B sp900066555	Lachnoclostridium B	Lachnospiraceae	Lachnospirales	Clostridia	Bacillota A	-0.072	0.019	1.3E-04	2897	95.3	-0.150	0.048	1.8E-03	561	95.5	-0.058	0.021	6.3E-03	2336	95.3	-0.050	0.015	1.1E-03	4338	95.6
Yeguia hominis	Yeguia	Acutalibacteraceae	Oscillospirales	Clostridia	Bacillota A	-0.077	0.020	1.5E-04	2897	72.9	-0.066	0.053	2.1E-01	561	71.3	-0.083	0.023	2.4E-04	2336	73.2	-0.074	0.017	9.4E-06	4338	66.6
UMGS1264 sp904399395	UMGS1264	Acutalibacteraceae	Oscillospirales	Clostridia	Bacillota A	0.074	0.020	1.5E-04	2897	83.1	0.105	0.049	3.3E-02	561	84.1	0.069	0.022	1.8E-03	2336	82.9	0.088	0.016	2.0E-08	4338	80.8
Agathobacter rectalis	Agathobacter	Lachnospiraceae	Lachnospirales	Clostridia	Bacillota A	-0.072	0.019	1.6E-04	2897	93.1	-0.077	0.049	1.2E-01	561	93.2	-0.074	0.021	5.1E-04	2336	93.1	-0.064	0.015	2.4E-05	4338	95.0
Anaerobutyricum soehngenii	Anaerobutyricum	Lachnospiraceae	Lachnospirales	Clostridia	Bacillota A	-0.074	0.020	1.8E-04	2897	80.3	-0.123	0.050	1.5E-02	561	78.6	-0.063	0.022	4.3E-03	2336	80.7	-0.073	0.016	3.9E-06	4338	82.1
ER4 sp003522105	ER4	Oscillospiraceae	Oscillospirales	Clostridia	Bacillota A	-0.092	0.025	1.8E-04	2897	30.4	-0.094	0.066	1.5E-01	561	28.3	-0.085	0.027	1.8E-03	2336	31.0	-0.088	0.019	6.3E-06	4338	31.7
Dysosmobacter sp004553545	Dysosmobacter	Oscillospiraceae	Oscillospirales	Clostridia	Bacillota A	-0.089	0.024	1.9E-04	2897	35.3	-0.014	0.060	8.1E-01	561	34.8	-0.101	0.027	1.3E-04	2336	35.4	-0.124	0.019	1.8E-10	4338	31.4
Eubacterium F sp003491505	Eubacterium F	Lachnospiraceae	Lachnospirales	Clostridia	Bacillota A	0.073	0.019	2.0E-04	2897	81.3	0.086	0.049	8.3E-02	561	81.1	0.072	0.022	1.0E-03	2336	81.3	0.057	0.016	3.5E-04	4338	77.5
Mediterraneibacter sp900541505	Mediterraneibacter	Lachnospiraceae	Lachnospirales	Clostridia	Bacillota A	0.084	0.023	2.2E-04	2897	41.5	0.016	0.058	7.8E-01	561	42.6	0.093	0.026	2.9E-04	2336	41.3	0.048	0.018	9.2E-03	4338	40.5
CAG-115 sp003531585	CAG-115	Ruminococcaceae	Oscillospirales	Clostridia	Bacillota A	0.078	0.021	2.3E-04	2897	59.9	0.069	0.053	2.0E-01	561	63.5	0.084	0.024	4.0E-04	2336	59.0	0.089	0.018	4.4E-07	4338	52.2
Ruminococcus D bicirculans	Ruminococcus D	Ruminococcaceae	Oscillospirales	Clostridia	Bacillota A	0.073	0.020	2.3E-04	2897	79.7	0.125	0.050	1.3E-02	561	79.7	0.061	0.022	5.8E-03	2336	79.7	0.089	0.016	2.8E-08	4338	76.0
Acetatifactor intestinalis	Acetatifactor	Lachnospiraceae	Lachnospirales	Clostridia	Bacillota A	-0.070	0.019	3.0E-04	2897	85.5	-0.036	0.050	4.7E-01	561	86.5	-0.075	0.022	6.2E-04	2336	85.2	-0.042	0.015	7.0E-03	4338	88.2
HGM13006 sp900756575	HGM13006	Oscillospiraceae	Oscillospirales	Clostridia	Bacillota A	-0.076	0.021	3.2E-04	2897	58.2	-0.118	0.054	2.8E-02	561	53.7	-0.067	0.024	4.9E-03	2336	59.3	-0.037	0.017	3.4E-02	4338	54.4
Turicibacter sanguinis	Turicibacter	Turicibacteraceae	Haloplasmatales	Bacilli	Bacillota	-0.078	0.022	3.4E-04	2897	51.6	-0.082	0.056	1.4E-01	561	50.4	-0.080	0.024	1.0E-03	2336	51.8	-0.088	0.017	3.8E-07	4338	52.7
RUG115 sp900066395	RUG115	Lachnospiraceae	Lachnospirales	Clostridia	Bacillota A	-0.070	0.020	3.7E-04	2897	80.7	-0.071	0.049	1.5E-01	561	80.4	-0.070	0.022	1.5E-03	2336	80.7	-0.059	0.016	1.4E-04	4338	86.4
Agathobaculum sp003481705	Agathobaculum	Butyricicoccaceae	Oscillospirales	Clostridia	Bacillota A	-0.077	0.022	4.2E-04	2897	50.7	-0.055	0.055	3.2E-01	561	52.2	-0.085	0.024	4.6E-04	2336	50.4	-0.033	0.018	6.0E-02	4338	48.8
Limiplasma merdipullorum	Limiplasma	CAG-74	Christensenellales	Clostridia	Bacillota A	0.071	0.020	4.3E-04	2897	71.1	0.063	0.051	2.2E-01	561	72.2	0.075	0.023	9.4E-04	2336	70.9	0.090	0.017	6.0E-08	4338	64.7
Roseburia hominis	Roseburia	Lachnospiraceae	Lachnospirales	Clostridia	Bacillota A	-0.066	0.019	5.2E-04	2897	94.9	-0.171	0.047	3.5E-04	561	95.0	-0.041	0.021	5.6E-02	2336	94.9	-0.043	0.015	5.0E-03	4338	94.9
CAG-103 sp900543625	CAG-103	Oscillospiraceae	Oscillospirales	Clostridia	Bacillota A	-0.083	0.024	5.4E-04	2897	32.7	-0.116	0.063	6.5E-02	561	32.6	-0.078	0.027	3.5E-03	2336	32.7	-0.079	0.019	2.8E-05	4338	34.7
Eisenbergiella sp900555195	Eisenbergiella	Lachnospiraceae	Lachnospirales	Clostridia	Bacillota A	0.076	0.022	5.5E-04	2897	47.2	0.064	0.055	2.5E-01	561	47.2	0.080	0.025	1.3E-03	2336	47.1	0.047	0.019	1.0E-02	4338	38.5
Thomasclavelia spiroformis	Thomasclavelia	Coprobacillaceae	Erysipelotrichales	Bacilli	Bacillota	0.084	0.024	5.6E-04	2897	30.7	0.132	0.062	3.6E-02	561	28.3	0.076	0.027	5.1E-03	2336	31.3	0.081	0.018	1.2E-05	4338	40.6
CAG-170 sp002404795	CAG-170	Oscillospiraceae	Oscillospirales	Clostridia	Bacillota A	-0.070	0.020	5.6E-04	2897	73.7	-0.046	0.051	3.6E-01	561	73.6	-0.073	0.023	1.3E-03	2336	73.7	-0.057	0.016	5.5E-04	4338	69.4
Fusicatenibacter saccharivorans	Fusicatenibacter	Lachnospiraceae	Lachnospirales	Clostridia	Bacillota A	-0.065	0.019	5.8E-04	2897	97.7	-0.053	0.048	2.7E-01	561	98.4	-0.067	0.021	1.5E-03	2336	97.6	-0.027	0.015	7.3E-02	4338	97.4
Merdisoma sp900553635	Merdisoma	Lachnospiraceae	Lachnospirales	Clostridia	Bacillota A	-0.074	0.021	5.9E-04	2897	54.5	-0.064	0.055	2.4E-01	561	55.6	-0.081	0.024	8.0E-04	2336	54.2	-0.075	0.017	1.4E-05	4338	55.1
Methanobrevibacter A smithii	Methanobrevibacter A	Methanobacteriaceae	Methanobacteriales	Methanobacteria	Methanobacteriota	-0.080	0.024	7.4E-04	2897	36.5	-0.044	0.061	4.7E-01	561	35.7	-0.088	0.027	9.7E-04	2336	36.6	-0.082	0.019	1.5E-05	4338	35.7
Pauljensenia bouchesdurhonensis	Pauljensenia	Actinomycetaceae	Actinomycetales	Actinomycetia	Actinomycetota	-0.076	0.023	7.6E-04	2897	42.4	-0.173	0.058	2.9E-03	561	46.2	-0.044	0.026	8.8E-02	2336	41.5	-0.075	0.017	1.7E-05	4338	50.7
Gordonibacter pamelaeae	Gordonibacter	Eggerthellaceae	Coriobacteriales	Coriobacteriia	Actinomycetota	0.070	0.021	7.8E-04	2897	63.1	-0.012	0.054	8.2E-01	561	64.0	0.089	0.023	1.4E-04	2336	62.9	0.079	0.017	4.4E-06	4338	54.8
Roseburia inulinivorans	Roseburia	Lachnospiraceae	Lachnospirales	Clostridia	Bacillota A	-0.064	0.019	8.0E-04	2897	94.6	-0.106	0.048	2.6E-02	561	93.8	-0.066	0.021	1.9E-03	2336	94.9	-0.022	0.015	1.4E-01	4338	96.0
Fimisoma sp000435715	Fimisoma	Anaerovoracaceae	Peptostreptococcales	Clostridia	Bacillota A	0.065	0.020	1.1E-03	2897	72.4	-0.018	0.051	7.3E-01	561	73.4	0.085	0.022	1.6E-04	2336	72.1	0.058	0.017	5.2E-04	4338	66.3
Heritagella sp905200265	Heritagella	Acutalibacteraceae	Oscillospirales	Clostridia	Bacillota A	0.078	0.024	1.1E-03	2897	33.4	0.126	0.059	3.4E-02	561	34.0	0.067	0.027	1.2E-02	2336	33.2	0.070	0.020	3.9E-04	4338	30.3
CAG-170 sp003516765	CAG-170	Oscillospiraceae	Oscillospirales	Clostridia	Bacillota A	-0.068	0.021	1.1E-03	2897	62.6	-0.033	0.054	5.4E-01	561	61.0	-0.074	0.023	1.6E-03	2336	63.0	-0.077	0.017	6.1E-06	4338	58.6

						Women					Premenopausal women					Postmenopausal women					Men				
**Species**	**Genus**	**Family**	**Order**	**Class**	**Phylum**	**β**	**SE**	**P**	**n**	**Prev**	**β**	**SE**	**P**	**n**	**Prev**	**β**	**SE**	**P**	**n**	**Prev**	**β**	**SE**	**P**	**n**	**Prev**
Vescimonas sp900552475	Vescimonas	Oscillospiraceae	Oscillospirales	Clostridia	Bacillota A	-0.067	0.021	1.1E-03	2897	66.6	-0.088	0.052	9.0E-02	561	68.1	-0.063	0.023	6.1E-03	2336	66.2	-0.043	0.017	9.8E-03	4338	64.5
Pauljensenia sp902373545	Pauljensenia	Actinomycetaceae	Actinomycetales	Actinomycetia	Actinomycetota	-0.069	0.021	1.2E-03	2897	56.1	-0.162	0.053	2.5E-03	561	60.1	-0.047	0.024	4.9E-02	2336	55.1	-0.078	0.017	4.8E-06	4338	57.2
Pauljensenia sp000466265	Pauljensenia	Actinomycetaceae	Actinomycetales	Actinomycetia	Actinomycetota	-0.069	0.021	1.3E-03	2897	54.2	-0.103	0.054	5.6E-02	561	58.3	-0.062	0.024	9.9E-03	2336	53.2	-0.071	0.017	4.2E-05	4338	53.3
Lawsonibacter sp014287875	Lawsonibacter	Oscillospiraceae	Oscillospirales	Clostridia	Bacillota A	0.065	0.020	1.3E-03	2897	72.1	0.057	0.051	2.7E-01	561	72.5	0.067	0.023	3.3E-03	2336	72.0	0.058	0.016	4.1E-04	4338	74.0
KLE1615 sp900066985	KLE1615	Lachnospiraceae	Lachnospirales	Clostridia	Bacillota A	-0.061	0.019	1.3E-03	2897	90.4	-0.087	0.048	6.8E-02	561	90.6	-0.060	0.021	5.1E-03	2336	90.3	-0.055	0.015	3.1E-04	4338	92.6
CAG-317 sp000433215	CAG-317	Lachnospiraceae	Lachnospirales	Clostridia	Bacillota A	-0.064	0.020	1.5E-03	2897	78.0	-0.120	0.051	2.0E-02	561	79.7	-0.054	0.022	1.5E-02	2336	77.7	-0.086	0.016	6.8E-08	4338	82.1
Guopingia tenuis	Guopingia	Christensenellaceae	Christensenellales	Clostridia	Bacillota A	0.061	0.019	1.5E-03	2897	93.1	0.033	0.049	5.1E-01	561	93.0	0.065	0.021	2.3E-03	2336	93.1	0.073	0.015	2.5E-06	4338	90.1
Massiliimalia timonensis	Massiliimalia	Ruminococcaceae	Oscillospirales	Clostridia	Bacillota A	0.064	0.020	1.7E-03	2897	67.9	0.039	0.052	4.6E-01	561	68.4	0.066	0.023	4.1E-03	2336	67.8	0.056	0.016	6.1E-04	4338	70.8
Christensenella minuta	Christensenella	Christensenellaceae	Christensenellales	Clostridia	Bacillota A	-0.071	0.023	1.7E-03	2897	43.7	-0.148	0.058	1.1E-02	561	43.5	-0.062	0.025	1.4E-02	2336	43.8	-0.022	0.018	2.2E-01	4338	43.5
Mediterraneibacter sp014287475	Mediterraneibacter	Lachnospiraceae	Lachnospirales	Clostridia	Bacillota A	0.066	0.021	1.9E-03	2897	57.6	-0.018	0.053	7.4E-01	561	61.3	0.079	0.024	8.7E-04	2336	56.7	0.033	0.017	5.2E-02	4338	56.3
Phil1 sp001940855	Phil1	CAG-138	Christensenellales	Clostridia	Bacillota A	-0.072	0.023	2.0E-03	2897	39.6	-0.102	0.058	8.2E-02	561	39.4	-0.057	0.026	3.0E-02	2336	39.6	-0.078	0.019	5.4E-05	4338	33.2
Acutalibacter ornithocaccae	Acutalibacter	Acutalibacteraceae	Oscillospirales	Clostridia	Bacillota A	0.064	0.021	2.1E-03	2897	63.0	0.131	0.053	1.4E-02	561	58.6	0.056	0.023	1.6E-02	2336	64.0	0.093	0.017	2.6E-08	4338	62.4
Blautia A faecis	Blautia A	Lachnospiraceae	Lachnospirales	Clostridia	Bacillota A	-0.057	0.019	2.4E-03	2897	98.4	-0.123	0.048	1.1E-02	561	98.8	-0.050	0.021	1.7E-02	2336	98.4	-0.011	0.015	4.7E-01	4338	98.7
Ruminococcus E bromii B	Ruminococcus E	Acutalibacteraceae	Oscillospirales	Clostridia	Bacillota A	-0.063	0.021	2.5E-03	2897	61.1	0.006	0.053	9.1E-01	561	63.3	-0.071	0.024	2.5E-03	2336	60.6	-0.055	0.017	1.3E-03	4338	58.3
Pygmaiobacter sp014287275	Pygmaiobacter	Ruminococcaceae	Oscillospirales	Clostridia	Bacillota A	0.061	0.020	2.6E-03	2897	73.6	0.056	0.051	2.8E-01	561	72.0	0.060	0.023	7.8E-03	2336	73.9	0.039	0.016	1.8E-02	4338	70.3
Veillonella rogosae	Veillonella	Veillonellaceae	Veillonellales	Negativicutes	Bacillota C	-0.071	0.024	2.7E-03	2897	33.9	-0.151	0.058	9.7E-03	561	35.8	-0.065	0.027	1.6E-02	2336	33.4	-0.045	0.019	1.8E-02	4338	35.8
Akkermansia muciniphila	Akkermansia	Akkermansiaceae	Verrucomicrobiales	Verrucomicrobiae	Verrucomicrobiota	0.063	0.021	2.7E-03	2897	60.6	-0.019	0.053	7.2E-01	561	57.8	0.088	0.023	1.7E-04	2336	61.3	0.082	0.017	1.1E-06	4338	58.9
Harryflintia acetispora	Harryflintia	Ruminococcaceae	Oscillospirales	Clostridia	Bacillota A	0.065	0.022	2.9E-03	2897	52.3	-0.002	0.054	9.7E-01	561	54.9	0.079	0.024	1.2E-03	2336	51.7	0.044	0.018	1.2E-02	4338	48.5
Onthomonas sp900545815	Onthomonas	Oscillospiraceae	Oscillospirales	Clostridia	Bacillota A	-0.066	0.022	2.9E-03	2897	46.5	-0.047	0.055	4.0E-01	561	47.8	-0.067	0.025	6.9E-03	2336	46.2	-0.056	0.018	2.0E-03	4338	45.4
Agathobaculum sp900291975	Agathobaculum	Butyricicoccaceae	Oscillospirales	Clostridia	Bacillota A	0.057	0.019	2.9E-03	2897	90.0	0.017	0.048	7.2E-01	561	90.9	0.062	0.022	4.2E-03	2336	89.7	0.094	0.015	9.9E-10	4338	89.2
Dysosmobacter sp944387015	Dysosmobacter	Oscillospiraceae	Oscillospirales	Clostridia	Bacillota A	0.059	0.020	3.2E-03	2897	76.1	0.127	0.051	1.3E-02	561	75.8	0.042	0.022	6.1E-02	2336	76.2	0.060	0.016	2.5E-04	4338	72.8
Coprococcus A catus	Coprococcus A	Lachnospiraceae	Lachnospirales	Clostridia	Bacillota A	-0.069	0.023	3.2E-03	2897	35.4	-0.047	0.059	4.3E-01	561	36.4	-0.075	0.026	4.2E-03	2336	35.1	-0.057	0.019	2.4E-03	4338	35.4
Pseudoscilispira sp018369915	Pseudoscilispira	Oscillospiraceae	Oscillospirales	Clostridia	Bacillota A	0.062	0.021	3.3E-03	2897	61.2	0.081	0.053	1.2E-01	561	61.9	0.056	0.023	1.7E-02	2336	61.0	0.077	0.017	3.5E-06	4338	64.5
Scatomorpha merdavium	Scatomorpha	Oscillospiraceae	Oscillospirales	Clostridia	Bacillota A	0.066	0.023	3.4E-03	2897	43.5	0.052	0.058	3.7E-01	561	43.5	0.070	0.025	5.2E-03	2336	43.5	0.090	0.019	3.1E-06	4338	33.3
Blautia A hydrogenotrophica	Blautia A	Lachnospiraceae	Lachnospirales	Clostridia	Bacillota A	0.066	0.023	3.5E-03	2897	43.3	-0.032	0.056	5.8E-01	561	44.7	0.084	0.025	9.0E-04	2336	42.9	0.045	0.018	1.3E-02	4338	41.2
Parachristensenella sp900542005	Parachristensenella	UMGS416	Christensenellales	Clostridia	Bacillota A	0.064	0.022	3.6E-03	2897	51.8	0.076	0.056	1.8E-01	561	50.6	0.066	0.024	6.8E-03	2336	52.1	0.071	0.018	7.0E-05	4338	46.4
Blautia A obeum	Blautia A	Lachnospiraceae	Lachnospirales	Clostridia	Bacillota A	-0.054	0.019	4.0E-03	2897	96.0	-0.116	0.047	1.5E-02	561	96.1	-0.047	0.021	2.5E-02	2336	96.0	-0.061	0.015	6.2E-05	4338	96.2
Pararuminococcus gallinarum	Pararuminococcus	Ruminococcaceae	Oscillospirales	Clostridia	Bacillota A	0.056	0.019	4.0E-03	2897	86.7	0.038	0.049	4.3E-01	561	88.2	0.061	0.022	4.9E-03	2336	86.3	0.067	0.016	1.9E-05	4338	83.9
Acetatifactor sp003447295	Acetatifactor	Lachnospiraceae	Lachnospirales	Clostridia	Bacillota A	-0.059	0.021	4.2E-03	2897	65.0	-0.109	0.052	3.5E-02	561	64.5	-0.051	0.023	2.7E-02	2336	65.1	-0.061	0.017	2.3E-04	4338	68.1
Ruminococcus B gnavus	Ruminococcus B	Lachnospiraceae	Lachnospirales	Clostridia	Bacillota A	0.062	0.022	5.0E-03	2897	48.3	0.018	0.057	7.5E-01	561	47.8	0.067	0.025	6.7E-03	2336	48.4	0.064	0.017	2.5E-04	4338	55.4
Merdibacter merdigallinarum	Merdibacter	Erysipelotrichaceae	Erysipelotrichales	Bacilli	Bacillota	0.055	0.020	5.3E-03	2897	77.3	0.097	0.050	5.6E-02	561	79.3	0.048	0.022	3.1E-02	2336	76.8	0.123	0.016	2.3E-14	4338	71.6
CAG-217 sp000436335	CAG-217	Acutalibacteraceae	Oscillospirales	Clostridia	Bacillota A	-0.064	0.023	5.5E-03	2897	39.1	-0.111	0.058	5.4E-02	561	37.6	-0.066	0.026	1.1E-02	2336	39.4	-0.081	0.018	1.1E-05	4338	41.0
Zhenpiania hominis	Zhenpiania	Anaerovoracaceae	Peptostreptococcales	Clostridia	Bacillota A	0.058	0.021	5.6E-03	2897	64.1	-0.026	0.054	6.4E-01	561	65.1	0.076	0.023	1.2E-03	2336	63.8	0.063	0.017	2.5E-04	4338	57.6
Angelakisella sp900547385	Angelakisella	Ruminococcaceae	Oscillospirales	Clostridia	Bacillota A	-0.060	0.022	6.1E-03	2897	51.6	-0.048	0.056	4.0E-01	561	51.2	-0.068	0.025	6.0E-03	2336	51.8	-0.066	0.018	2.6E-04	4338	46.7
Faecalibacterium prausnitzii J	Faecalibacterium	Ruminococcaceae	Oscillospirales	Clostridia	Bacillota A	0.062	0.023	7.1E-03	2897	41.4	0.100	0.057	8.2E-02	561	45.8	0.051	0.026	5.0E-02	2336	40.4	0.130	0.019	9.0E-12	4338	35.2
Dialister invisus	Dialister	Dialisteraceae	Veillonellales	Negativicutes	Bacillota C	-0.060	0.022	7.2E-03	2897	45.8	-0.103	0.056	6.8E-02	561	46.9	-0.045	0.025	7.3E-02	2336	45.6	0.018	0.018	3.2E-01	4338	43.1
Agathobacter sp000434275	Agathobacter	Lachnospiraceae	Lachnospirales	Clostridia	Bacillota A	-0.062	0.023	7.3E-03	2897	38.2	-0.033	0.060	5.8E-01	561	34.4	-0.069	0.026	7.4E-03	2336	39.1	-0.029	0.019	1.3E-01	4338	35.5
Ruthenibacterium lactatiformans	Ruthenibacterium	Ruminococcaceae	Oscillospirales	Clostridia	Bacillota A	0.050	0.019	7.3E-03	2897	99.2	-0.020	0.049	6.8E-01	561	99.6	0.065	0.021	1.9E-03	2336	99.1	0.089	0.015	3.8E-09	4338	99.0
Eubacterium F sp000433735	Eubacterium F	Lachnospiraceae	Lachnospirales	Clostridia	Bacillota A	0.059	0.022	7.7E-03	2897	49.7	0.024	0.055	6.6E-01	561	50.1	0.068	0.025	5.7E-03	2336	49.6	0.078	0.018	2.0E-05	4338	43.2
Alectryocaccomicrobium faecavium	Alectryocaccomicrobium	CAG-74	Christensenellales	Clostridia	Bacillota A	0.060	0.023	8.6E-03	2897	39.7	0.052	0.059	3.8E-01	561	39.8	0.063	0.026	1.4E-02	2336	39.7	0.096	0.020	9.4E-07	4338	30.5
Oxalobacter sp900760095	Oxalobacter	Burkholderiaceae	Burkholderiales	Gammaproteobacteria	Pseudomonadota	0.061	0.023	8.9E-03	2897	38.2	0.152	0.057	8.2E-03	561	40.1	0.035	0.026	1.8E-01	2336	37.7	0.052	0.019	6.2E-03	4338	36.2
CAG-170 sp900553545	CAG-170	Oscillospiraceae	Oscillospirales	Clostridia	Bacillota A	-0.052	0.020	9.6E-03	2897	73.9	0.026	0.052	6.2E-01	561	75.2	-0.073	0.022	1.1E-03	2336	73.5	-0.027	0.016	1.0E-01	4338	71.8
Scatomorpha intestinigallinarum	Scatomorpha	Oscillospiraceae	Oscillospirales	Clostridia	Bacillota A	0.060	0.023	1.0E-02	2897	36.5	0.017	0.059	7.7E-01	561	36.9	0.067	0.026	1.0E-02	2336	36.4	0.105	0.020	1.0E-07	4338	30.2
Bianquea renquensis	Bianquea	Bianqueaceae	Lachnospirales	Clostridia	Bacillota A	0.058	0.023	1.0E-02	2897	43.1	0.059	0.058	3.1E-01	561	40.3	0.059	0.025	1.9E-02	2336	43.8	0.119	0.019	4.4E-10	4338	34.4
Emergencia sp904420065	Emergencia	Anaerovoracaceae	Peptostreptococcales	Clostridia	Bacillota A	0.058	0.023	1.2E-02	2897	42.0	0.009	0.057	8.8E-01	561	44.0	0.068	0.026	7.9E-03	2336	41.6	0.056	0.019	3.0E-03	4338	37.5
CAG-170 sp000432135	CAG-170	Oscillospiraceae	Oscillospirales	Clostridia	Bacillota A	-0.051	0.020	1.2E-02	2897	68.9	-0.040	0.053	4.5E-01	561	68.8	-0.056	0.023	1.5E-02	2336	68.9	-0.023	0.017	1.6E-01	4338	66.9
Faecimorpha stercoravium	Faecimorpha	Bianqueaceae	Lachnospirales	Clostridia	Bacillota A	0.057	0.023	1.2E-02	2897	42.8	0.063	0.058	2.7E-01	561	41.4	0.053	0.025	3.5E-02	2336	43.1	0.060	0.019	1.4E-03	4338	35.5
CAJJPW01 sp905193725	CAJJPW01	Christensenellaceae	Christensenellales	Clostridia	Bacillota A	0.051	0.021	1.3E-02	2897	65.0	0.062	0.053	2.4E-01	561	65.4	0.052	0.023	2.4E-02	2336	64.9	0.096	0.017	1.9E-08	4338	57.0
PeH17 sp000435055	PeH17	CAG-138	Christensenellales	Clostridia	Bacillota A	0.051	0.021	1.5E-02	2897	60.4	0.067	0.054	2.1E-01	561	59.9	0.051	0.024	2.9E-02	2336	60.6	0.015	0.017	3.8E-01	4338	52.1
Acutalibacter sp905215055	Acutalibacter	Acutalibacteraceae	Oscillospirales	Clostridia	Bacillota A	0.055	0.023	1.5E-02	2897	41.1	0.030	0.058	6.1E-01	561	42.8	0.058	0.026	2.4E-02	2336	40.8	0.080	0.019	2.1E-05	4338	37.0
JAGZMM01 sp018364155	JAGZMM01	Acutalibacteraceae	Oscillospirales	Clostridia	Bacillota A	-0.055	0.023	1.6E-02	2897	42.2	-0.113	0.059	5.7E-02	561	38.5	-0.049	0.025	5.2E-02	2336	43.1	-0.008	0.019	6.6E-01	4338	35.3
UMGS1312 sp900550625	UMGS1312	CAG-272	Oscillospirales	Clostridia	Bacillota A	-0.055	0.023	1.9E-02	2897	37.6	-0.019	0.061	7.6E-01	561	34.6	-0.062	0.026	1.7E-02	2336	38.3	-0.065	0.019	6.8E-04	4338	34.6
Anaerotardibacter sp000435675	Anaerotardibacter	Eggerthellaceae	Coriobacteriales	Coriobacteriia	Actinomycetota	0.053	0.023	2.2E-02	2897	39.0	0.010	0.058	8.6E-01	561	40.5	0.053	0.026	3.9E-02	2336	38.7	0.063	0.019	8.0E-04	4338	35.9
Streptococcus thermophilus	Streptococcus	Streptococcaceae	Lactobacillales	Bacilli	Bacillota	-0.045	0.020	2.5E-02	2897	75.7	-0.124	0.052	1.7E-02	561	75.9	-0.030	0.022	1.9E-01	2336	75.6	-0.065	0.017	9.6E-05	4338	66.3
Gordonibacter urolithinfaciens	Gordonibacter	Eggerthellaceae	Coriobacteriales	Coriobacteriia	Actinomycetota	0.051	0.023	2.5E-02	2897	41.7	-0.062	0.058	2.9E-01	561	41.5	0.069	0.025	6.4E-03	2336	41.8	0.069	0.019	2.1E-04	4338	38.3
RGIG4076 sp905198155	RGIG4076	Acutalibacteraceae	Oscillospirales	Clostridia	Bacillota A	0.051	0.023	2.6E-02	2897	39.6	0.120	0.056	3.4E-02	561	40.8	0.045	0.026	8.0E-02	2336	39.3	0.017	0.019	3.7E-01	4338	35.9
Eisenbergiella sp900066775	Eisenbergiella	Lachnospiraceae	Lachnospirales	Clostridia	Bacillota A	-0.042	0.019	2.7E-02	2897	92.0	-0.021	0.047	6.5E-01	561	93.0	-0.048	0.021	2.3E-02	2336	91.8	0.017	0.015	2.7E-01	4338	91.6
Ligaoa zhengdingensis	Ligaoa	Ruminococcaceae	Oscillospirales	Clostridia	Bacillota A	0.048	0.022	2.8E-02	2897	49.6	0.033	0.055	5.5E-01	561	52.0	0.058	0.024	1.8E-02	2336	49.0	0.074	0.018	3.6E-05	4338	46.9

						Women					Premenopausal women					Postmenopausal women					Men				
**Species**	**Genus**	**Family**	**Order**	**Class**	**Phylum**	**β**	**SE**	**P**	**n**	**Prev**	**β**	**SE**	**P**	**n**	**Prev**	**β**	**SE**	**P**	**n**	**Prev**	**β**	**SE**	**P**	**n**	**Prev**
Anaerotruncus sp014385085	Anaerotruncus	Ruminococcaceae	Oscillospirales	Clostridia	Bacillota A	0.043	0.020	3.0E-02	2897	74.8	0.069	0.050	1.7E-01	561	75.4	0.038	0.022	9.3E-02	2336	74.7	0.025	0.016	1.3E-01	4338	71.0
Blautia A sp000436615	Blautia A	Lachnospiraceae	Lachnospirales	Clostridia	Bacillota A	0.046	0.021	3.2E-02	2897	59.4	-0.004	0.054	9.4E-01	561	60.8	0.055	0.024	2.1E-02	2336	59.0	0.024	0.017	1.5E-01	4338	56.1
UMGS1975 sp900546685	UMGS1975	QAND01	Christensenellales	Clostridia	Bacillota A	0.042	0.020	3.2E-02	2897	83.2	0.023	0.049	6.3E-01	561	83.8	0.043	0.022	5.1E-02	2336	83.0	0.080	0.016	4.8E-07	4338	79.0
Lawsonibacter sp900545895	Lawsonibacter	Oscillospiraceae	Oscillospirales	Clostridia	Bacillota A	0.047	0.022	3.3E-02	2897	49.1	0.085	0.056	1.3E-01	561	51.3	0.037	0.025	1.3E-01	2336	48.5	0.112	0.018	5.8E-10	4338	44.5
UBA866 sp900543295	UBA866	Ruminococcaceae	Oscillospirales	Clostridia	Bacillota A	0.044	0.021	3.4E-02	2897	63.5	-0.008	0.053	8.9E-01	561	66.1	0.058	0.023	1.3E-02	2336	62.9	0.055	0.017	1.2E-03	4338	59.7
Holdemania massiliensis	Holdemania	Erysipelotrichaceae	Erysipelotrichales	Bacilli	Bacillota	0.048	0.023	3.4E-02	2897	43.3	0.010	0.057	8.5E-01	561	43.3	0.059	0.025	2.0E-02	2336	43.2	0.022	0.018	2.0E-01	4338	50.0
Faecalibacterium sp900539885	Faecalibacterium	Ruminococcaceae	Oscillospirales	Clostridia	Bacillota A	-0.042	0.020	3.5E-02	2897	74.9	-0.102	0.051	4.8E-02	561	77.9	-0.028	0.022	2.2E-01	2336	74.2	-0.058	0.016	4.2E-04	4338	72.8
Anaerotignum faecicola	Anaerotignum	Anaerotignaceae	Lachnospirales	Clostridia	Bacillota A	0.042	0.020	3.8E-02	2897	69.2	0.015	0.051	7.7E-01	561	73.3	0.047	0.023	4.0E-02	2336	68.3	0.093	0.016	1.4E-08	4338	70.0
Sellimonas intestinalis	Sellimonas	Lachnospiraceae	Lachnospirales	Clostridia	Bacillota A	0.049	0.024	3.8E-02	2897	37.5	-0.026	0.060	6.6E-01	561	34.6	0.063	0.026	1.7E-02	2336	38.1	0.084	0.019	8.2E-06	4338	37.0
Lentihominibacter excrementipullorum	Lentihominibacter	Anaerovoracaceae	Peptostreptococcales	Clostridia	Bacillota A	0.044	0.021	4.0E-02	2897	60.3	-0.006	0.053	9.1E-01	561	62.6	0.050	0.024	3.4E-02	2336	59.8	0.021	0.017	2.2E-01	4338	54.8
CAG-45 sp000438375	CAG-45	Lachnospiraceae	Lachnospirales	Clostridia	Bacillota A	-0.046	0.023	4.0E-02	2897	44.9	-0.069	0.058	2.3E-01	561	44.6	-0.045	0.025	7.8E-02	2336	45.0	-0.003	0.019	8.8E-01	4338	38.8
Dysosmobacter excrementavium	Dysosmobacter	Oscillospiraceae	Oscillospirales	Clostridia	Bacillota A	0.047	0.024	4.5E-02	2897	35.0	0.059	0.060	3.3E-01	561	33.5	0.045	0.026	9.0E-02	2336	35.4	0.095	0.019	7.8E-07	4338	33.2
COE1 sp001916965	COE1	Lachnospiraceae	Lachnospirales	Clostridia	Bacillota A	-0.045	0.023	4.8E-02	2897	42.4	0.026	0.056	6.4E-01	561	43.7	-0.057	0.025	2.6E-02	2336	42.1	-0.008	0.019	6.7E-01	4338	39.7
Gemmiger qucibialis	Gemmiger	Ruminococcaceae	Oscillospirales	Clostridia	Bacillota A	-0.037	0.019	4.8E-02	2897	94.1	-0.028	0.049	5.7E-01	561	94.3	-0.041	0.021	5.1E-02	2336	94.0	0.029	0.015	6.1E-02	4338	94.4
Bifidobacterium longum	Bifidobacterium	Bifidobacteriaceae	Actinomycetales	Actinomycetia	Actinomycetota	-0.038	0.020	4.9E-02	2897	86.1	0.002	0.051	9.7E-01	561	87.2	-0.038	0.022	8.4E-02	2336	85.8	0.025	0.016	1.1E-01	4338	89.4
SFEL01 sp004557245	SFEL01	CAG-138	Christensenellales	Clostridia	Bacillota A	-0.041	0.021	5.4E-02	2897	58.0	-0.015	0.055	7.9E-01	561	57.9	-0.041	0.024	8.8E-02	2336	58.0	-0.050	0.018	4.7E-03	4338	51.5
Merdisoma sp900066385	Merdisoma	Lachnospiraceae	Lachnospirales	Clostridia	Bacillota A	-0.038	0.020	5.8E-02	2897	77.6	-0.049	0.049	3.2E-01	561	78.1	-0.033	0.022	1.4E-01	2336	77.5	-0.042	0.016	9.2E-03	4338	79.0
QANA01 sp900554725	QANA01	Christensenellaceae	Christensenellales	Clostridia	Bacillota A	0.043	0.023	5.8E-02	2897	40.0	0.012	0.058	8.4E-01	561	40.6	0.044	0.026	8.6E-02	2336	39.8	0.010	0.019	5.9E-01	4338	34.0
Zhenpiania massiliensis	Zhenpiania	Anaerovoracaceae	Peptostreptococcales	Clostridia	Bacillota A	0.045	0.024	5.9E-02	2897	34.5	0.039	0.061	5.3E-01	561	32.4	0.047	0.026	7.6E-02	2336	35.0	0.016	0.020	4.2E-01	4338	31.4
Ruthenibacterium merdipullorum	Ruthenibacterium	Ruminococcaceae	Oscillospirales	Clostridia	Bacillota A	0.039	0.021	6.2E-02	2897	63.4	-0.023	0.052	6.6E-01	561	68.1	0.055	0.023	2.0E-02	2336	62.3	-0.001	0.017	9.7E-01	4338	58.9
Metalachnospira sp900553995	Metalachnospira	Anaerotignaceae	Lachnospirales	Clostridia	Bacillota A	-0.040	0.022	6.5E-02	2897	55.3	-0.014	0.055	8.0E-01	561	57.6	-0.048	0.024	4.6E-02	2336	54.8	0.001	0.017	9.6E-01	4338	54.4
Eubacterium G ventriosum	Eubacterium G	Lachnospiraceae	Lachnospirales	Clostridia	Bacillota A	-0.037	0.020	6.8E-02	2897	73.0	-0.137	0.050	6.7E-03	561	73.1	-0.016	0.022	4.7E-01	2336	73.0	0.004	0.016	8.2E-01	4338	77.7
Butyribacter sp003529475	Butyribacter	Lachnospiraceae	Lachnospirales	Clostridia	Bacillota A	-0.037	0.020	7.0E-02	2897	70.8	-0.029	0.052	5.8E-01	561	70.4	-0.039	0.023	9.1E-02	2336	70.9	-0.004	0.016	8.3E-01	4338	73.4
Faecalibacterium longum	Faecalibacterium	Ruminococcaceae	Oscillospirales	Clostridia	Bacillota A	0.034	0.019	7.1E-02	2897	94.5	0.073	0.049	1.3E-01	561	95.7	0.025	0.021	2.4E-01	2336	94.2	0.071	0.015	3.3E-06	4338	93.7
Blautia A sp003477525	Blautia A	Lachnospiraceae	Lachnospirales	Clostridia	Bacillota A	-0.042	0.023	7.4E-02	2897	38.0	-0.060	0.059	3.1E-01	561	40.1	-0.042	0.026	1.1E-01	2336	37.5	0.004	0.018	8.2E-01	4338	45.7
ER4 sp900317525	ER4	Oscillospiraceae	Oscillospirales	Clostridia	Bacillota A	0.035	0.020	7.4E-02	2897	79.7	0.045	0.051	3.7E-01	561	79.7	0.035	0.022	1.2E-01	2336	79.7	0.001	0.016	9.6E-01	4338	76.6
Ruminiclostridium E siraeum	Ruminiclostridium E	Ruminococcaceae	Oscillospirales	Clostridia	Bacillota A	0.036	0.020	7.8E-02	2897	71.4	0.021	0.051	6.8E-01	561	71.5	0.036	0.023	1.1E-01	2336	71.4	0.047	0.016	3.0E-03	4338	77.0
Gemmiger formicilis	Gemmiger	Ruminococcaceae	Oscillospirales	Clostridia	Bacillota A	-0.034	0.019	7.8E-02	2897	90.9	-0.084	0.048	8.5E-02	561	91.8	-0.024	0.021	2.6E-01	2336	90.7	-0.031	0.015	4.3E-02	4338	90.8
Egerieenecus merdigallinarum	Egerieenecus	CAG-74	Christensenellales	Clostridia	Bacillota A	0.036	0.021	7.8E-02	2897	65.9	0.009	0.053	8.6E-01	561	66.0	0.044	0.023	5.6E-02	2336	65.9	0.065	0.017	1.0E-04	4338	60.9
Coprococcus eutactus A	Coprococcus	Lachnospiraceae	Lachnospirales	Clostridia	Bacillota A	-0.039	0.022	8.0E-02	2897	49.7	0.024	0.057	6.7E-01	561	46.3	-0.050	0.025	4.0E-02	2336	50.5	-0.062	0.018	5.3E-04	4338	48.1
CAG-245 sp000435175	CAG-245	CAG-508	TANB77	Clostridia	Bacillota A	-0.037	0.022	8.5E-02	2897	53.5	-0.043	0.054	4.3E-01	561	54.4	-0.039	0.024	1.1E-01	2336	53.3	-0.041	0.018	1.9E-02	4338	52.2
Eubacterium I ramulus A	Eubacterium I	Lachnospiraceae	Lachnospirales	Clostridia	Bacillota A	-0.041	0.024	9.3E-02	2897	30.9	-0.024	0.060	6.9E-01	561	35.1	-0.049	0.027	7.2E-02	2336	29.8	-0.075	0.020	1.4E-04	4338	30.2
Blautia A sp900066145	Blautia A	Lachnospiraceae	Lachnospirales	Clostridia	Bacillota A	-0.033	0.020	1.1E-01	2897	71.4	-0.102	0.050	4.2E-02	561	73.4	-0.019	0.023	3.9E-01	2336	70.9	0.039	0.016	1.6E-02	4338	70.0
CAG-238 sp900542245	CAG-238	Anaerovoracaceae	Peptostreptococcales	Clostridia	Bacillota A	-0.033	0.021	1.1E-01	2897	63.5	-0.102	0.054	5.8E-02	561	62.2	-0.017	0.023	4.6E-01	2336	63.8	-0.042	0.017	1.4E-02	4338	61.3
Lachnospira rogosae	Lachnospira	Lachnospiraceae	Lachnospirales	Clostridia	Bacillota A	0.033	0.021	1.1E-01	2897	66.2	0.028	0.053	5.9E-01	561	64.7	0.031	0.023	1.9E-01	2336	66.6	0.068	0.017	3.6E-05	4338	67.5
Dysosmobacter A sp014334055	Dysosmobacter A	Oscillospiraceae	Oscillospirales	Clostridia	Bacillota A	0.035	0.023	1.2E-01	2897	43.1	0.074	0.059	2.1E-01	561	41.9	0.033	0.025	2.0E-01	2336	43.5	0.052	0.019	6.2E-03	4338	35.6
Schaedlerella glycyrrhizinilytica A	Schaedlerella	Lachnospiraceae	Lachnospirales	Clostridia	Bacillota A	0.038	0.024	1.2E-01	2897	31.7	-0.017	0.062	7.9E-01	561	30.7	0.048	0.027	7.6E-02	2336	31.9	0.044	0.019	1.9E-02	4338	39.1
HGM12669 sp900761935	HGM12669	CAG-272	Oscillospirales	Clostridia	Bacillota A	0.035	0.023	1.2E-01	2897	42.0	0.069	0.056	2.1E-01	561	45.5	0.033	0.025	1.9E-01	2336	41.1	0.046	0.019	1.5E-02	4338	36.1
Merdibacter merdipullorum	Merdibacter	Erysipelotrichaceae	Erysipelotrichales	Bacilli	Bacillota	0.035	0.023	1.3E-01	2897	40.2	0.053	0.058	3.6E-01	561	40.6	0.039	0.026	1.3E-01	2336	40.2	0.075	0.019	7.8E-05	4338	35.0
CAG-269 sp003525075	CAG-269	CAG-508	TANB77	Clostridia	Bacillota A	-0.034	0.023	1.4E-01	2897	40.2	-0.012	0.057	8.3E-01	561	41.9	-0.044	0.026	9.2E-02	2336	39.9	-0.047	0.019	1.2E-02	4338	37.3
Christensenella hongkongensis	Christensenella	Christensenellaceae	Christensenellales	Clostridia	Bacillota A	0.034	0.023	1.4E-01	2897	39.7	0.006	0.058	9.2E-01	561	46.7	0.039	0.026	1.4E-01	2336	38.1	0.026	0.019	1.6E-01	4338	37.9
Copromonas sp900066055	Copromonas	Lachnospiraceae	Lachnospirales	Clostridia	Bacillota A	-0.032	0.023	1.5E-01	2897	43.9	0.014	0.059	8.1E-01	561	42.2	-0.041	0.025	1.1E-01	2336	44.3	-0.024	0.018	1.9E-01	4338	44.7
Vescimonas sp001916855	Vescimonas	Oscillospiraceae	Oscillospirales	Clostridia	Bacillota A	0.032	0.022	1.5E-01	2897	47.5	0.036	0.056	5.1E-01	561	46.9	0.037	0.025	1.4E-01	2336	47.6	0.020	0.018	2.6E-01	4338	43.9
HGM13222 sp900757485	HGM13222	Ruminococcaceae	Oscillospirales	Clostridia	Bacillota A	0.030	0.022	1.7E-01	2897	49.9	0.082	0.055	1.4E-01	561	49.7	0.020	0.024	4.2E-01	2336	49.9	0.047	0.018	9.3E-03	4338	48.0
UBA11524 sp000437595	UBA11524	CAG-74	Christensenellales	Clostridia	Bacillota A	-0.028	0.021	1.8E-01	2897	65.9	-0.040	0.052	4.4E-01	561	68.6	-0.023	0.023	3.3E-01	2336	65.2	-0.024	0.017	1.5E-01	4338	64.8
Pseudobutyricicoccus lothianensis	Pseudobutyricicoccus	Butyricicoccaceae	Oscillospirales	Clostridia	Bacillota A	0.028	0.021	1.8E-01	2897	58.3	0.071	0.054	1.9E-01	561	59.0	0.018	0.024	4.6E-01	2336	58.1	0.058	0.017	7.4E-04	4338	54.0
Faecousia sp900544405	Faecousia	Oscillospiraceae	Oscillospirales	Clostridia	Bacillota A	-0.029	0.022	1.8E-01	2897	48.6	-0.037	0.057	5.2E-01	561	51.2	-0.033	0.025	1.8E-01	2336	48.0	-0.070	0.018	9.0E-05	4338	47.3
Pullichristensenella sp904420265	Pullichristensenella	CAG-74	Christensenellales	Clostridia	Bacillota A	0.030	0.022	1.9E-01	2897	44.7	0.024	0.057	6.7E-01	561	45.3	0.027	0.025	2.8E-01	2336	44.6	0.042	0.019	2.8E-02	4338	35.3
Muricomes sp000509105	Muricomes	Lachnospiraceae	Lachnospirales	Clostridia	Bacillota A	0.027	0.021	1.9E-01	2897	67.4	-0.025	0.053	6.4E-01	561	71.1	0.037	0.023	1.0E-01	2336	66.6	0.045	0.017	6.8E-03	4338	63.2
Vescimonas coprocola	Vescimonas	Oscillospiraceae	Oscillospirales	Clostridia	Bacillota A	0.024	0.019	2.1E-01	2897	87.1	-0.039	0.050	4.3E-01	561	88.8	0.029	0.022	1.8E-01	2336	86.7	0.068	0.015	1.1E-05	4338	88.9
F23-B02 sp001916715	F23-B02	Oscillospiraceae	Oscillospirales	Clostridia	Bacillota A	-0.029	0.023	2.1E-01	2897	38.7	-0.080	0.059	1.8E-01	561	38.0	-0.020	0.026	4.4E-01	2336	38.9	0.016	0.020	4.1E-01	4338	31.9
Blautia A sp900066205	Blautia A	Lachnospiraceae	Lachnospirales	Clostridia	Bacillota A	-0.026	0.021	2.2E-01	2897	61.9	0.036	0.052	4.9E-01	561	64.2	-0.040	0.024	8.8E-02	2336	61.3	-0.018	0.017	3.0E-01	4338	58.3
Neobittarella massiliensis	Neobittarella	Ruminococcaceae	Oscillospirales	Clostridia	Bacillota A	0.030	0.024	2.2E-01	2897	31.1	-0.132	0.059	2.6E-02	561	36.4	0.061	0.027	2.6E-02	2336	29.8	0.044	0.019	1.9E-02	4338	36.8
RGIG7067 sp944384895	RGIG7067	Acutalibacteraceae	Oscillospirales	Clostridia	Bacillota A	0.025	0.021	2.2E-01	2897	64.2	-0.002	0.053	9.6E-01	561	62.9	0.030	0.023	1.9E-01	2336	64.6	0.081	0.017	1.3E-06	4338	63.3
Escherichia coli	Escherichia	Enterobacteriaceae	Enterobacterales	Gammaproteobacteria	Pseudomonadota	0.024	0.020	2.3E-01	2897	80.9	0.034	0.052	5.2E-01	561	77.5	0.028	0.022	1.9E-01	2336	81.8	0.034	0.016	3.2E-02	4338	81.3
Eubacterium G sp000434315	Eubacterium G	Lachnospiraceae	Lachnospirales	Clostridia	Bacillota A	0.026	0.022	2.3E-01	2897	52.5	-0.016	0.056	7.8E-01	561	51.5	0.041	0.024	9.0E-02	2336	52.7	0.085	0.018	2.6E-06	4338	44.5
Hominisplanchenecus faecis	Hominisplanchenecus	Lachnospiraceae	Lachnospirales	Clostridia	Bacillota A	-0.023	0.020	2.4E-01	2897	79.3	-0.055	0.050	2.8E-01	561	78.8	-0.025	0.022	2.6E-01	2336	79.4	-0.017	0.016	2.7E-01	4338	82.1
Massilistercora timonensis	Massilistercora	Lachnospiraceae	Lachnospirales	Clostridia	Bacillota A	0.027	0.023	2.5E-01	2897	38.9	-0.012	0.059	8.4E-01	561	37.4	0.044	0.026	9.0E-02	2336	39.3	0.080	0.019	2.8E-05	4338	34.3

						Women					Premenopausal women					Postmenopausal women					Men				
Species	Genus	Family	Order	Class	Phylum	β	SE	P	n	Prev	β	SE	P	n	Prev	β	SE	P	n	Prev	β	SE	P	n	Prev
UBA3818 sp900557155	UBA3818	Ruminococcaceae	Oscillospirales	Clostridia	Bacillota A	-0.023	0.020	2.6E-01	2897	76.0	-0.046	0.051	3.6E-01	561	77.7	-0.017	0.022	4.4E-01	2336	75.6	-0.026	0.016	1.1E-01	4338	73.7
Choladocola sp003481535	Choladocola	Lachnospiraceae	Lachnospirales	Clostridia	Bacillota A	0.022	0.019	2.6E-01	2897	87.1	0.037	0.050	4.6E-01	561	87.5	0.018	0.022	4.0E-01	2336	87.0	0.038	0.016	1.4E-02	4338	88.4
Holdemania sp900120005	Holdemania	Erysipelotrichaceae	Erysipelotrichales	Bacilli	Bacillota	0.025	0.022	2.6E-01	2897	49.7	-0.091	0.055	9.9E-02	561	49.6	0.040	0.024	1.0E-01	2336	49.8	0.070	0.018	6.9E-05	4338	50.5
CAG-273 sp000438355	CAG-273	CAG-508	TANB77	Clostridia	Bacillota A	-0.024	0.022	2.9E-01	2897	44.0	-0.028	0.058	6.3E-01	561	41.7	-0.026	0.025	3.0E-01	2336	44.5	0.016	0.019	3.8E-01	4338	37.6
Choladocola sp018223365	Choladocola	Lachnospiraceae	Lachnospirales	Clostridia	Bacillota A	-0.021	0.020	3.0E-01	2897	80.0	-0.028	0.050	5.8E-01	561	81.1	-0.021	0.022	3.4E-01	2336	79.8	-0.008	0.016	6.0E-01	4338	80.8
Butyribacter intestini	Butyribacter	Lachnospiraceae	Lachnospirales	Clostridia	Bacillota A	-0.023	0.022	3.0E-01	2897	48.8	0.014	0.056	8.0E-01	561	48.7	-0.030	0.025	2.2E-01	2336	48.8	0.034	0.018	5.3E-02	4338	48.9
Faecalibacterium prausnitzii D	Faecalibacterium	Ruminococcaceae	Oscillospirales	Clostridia	Bacillota A	-0.019	0.019	3.1E-01	2897	95.5	-0.083	0.048	8.6E-02	561	96.4	-0.002	0.021	9.1E-01	2336	95.3	-0.019	0.015	2.0E-01	4338	94.6
Vescimonas sp000435555	Vescimonas	Oscillospiraceae	Oscillospirales	Clostridia	Bacillota A	-0.021	0.021	3.1E-01	2897	59.6	0.020	0.053	7.1E-01	561	57.9	-0.031	0.024	1.9E-01	2336	60.0	-0.016	0.017	3.7E-01	4338	54.4
CAG-170 sp900549635	CAG-170	Oscillospiraceae	Oscillospirales	Clostridia	Bacillota A	0.020	0.020	3.2E-01	2897	75.7	0.055	0.051	2.9E-01	561	74.3	0.014	0.022	5.3E-01	2336	76.1	-0.003	0.016	8.5E-01	4338	71.9
Geddesella stercoravicola	Geddesella	UBA644	Oscillospirales	Clostridia	Bacillota A	0.022	0.022	3.3E-01	2897	47.4	0.001	0.054	9.9E-01	561	49.2	0.027	0.025	2.7E-01	2336	46.9	0.052	0.018	4.0E-03	4338	45.2
CAJFUR01 sp904420575	CAJFUR01	Eggerthellaceae	Coriobacteriales	Coriobacteriia	Actinomycetota	0.019	0.019	3.3E-01	2897	89.0	-0.050	0.051	3.2E-01	561	89.3	0.034	0.022	1.2E-01	2336	88.9	0.029	0.016	6.5E-02	4338	85.5
UBA11774 sp003507655	UBA11774	Lachnospiraceae	Lachnospirales	Clostridia	Bacillota A	-0.018	0.019	3.5E-01	2897	90.4	-0.036	0.050	4.7E-01	561	90.7	-0.011	0.022	6.2E-01	2336	90.4	0.002	0.016	9.0E-01	4338	87.0
Enterocloster sp000431375	Enterocloster	Lachnospiraceae	Lachnospirales	Clostridia	Bacillota A	0.018	0.019	3.6E-01	2897	89.4	-0.032	0.049	5.1E-01	561	89.7	0.026	0.022	2.3E-01	2336	89.3	0.047	0.016	2.4E-03	4338	88.7
Faecousia sp000435995	Faecousia	Oscillospiraceae	Oscillospirales	Clostridia	Bacillota A	-0.020	0.022	3.6E-01	2897	53.5	-0.044	0.053	4.1E-01	561	53.8	-0.011	0.024	6.6E-01	2336	53.4	0.044	0.018	1.6E-02	4338	42.3
Blautia A sp900548245	Blautia A	Lachnospiraceae	Lachnospirales	Clostridia	Bacillota A	-0.020	0.022	3.6E-01	2897	50.1	-0.018	0.057	7.6E-01	561	53.7	-0.021	0.025	4.0E-01	2336	49.2	0.012	0.017	4.8E-01	4338	52.5
Lawsonibacter sp900549405	Lawsonibacter	Oscillospiraceae	Oscillospirales	Clostridia	Bacillota A	0.019	0.021	3.6E-01	2897	65.8	0.058	0.054	2.9E-01	561	66.5	0.015	0.023	5.2E-01	2336	65.6	0.052	0.017	1.6E-03	4338	67.6
Faecalibacillus intestinalis	Faecalibacillus	Coprobacillaceae	Erysipelotrichales	Bacilli	Bacillota	0.017	0.019	3.8E-01	2897	87.4	-0.060	0.049	2.2E-01	561	88.4	0.027	0.022	2.1E-01	2336	87.2	0.017	0.016	2.7E-01	4338	86.0
Massiliimalia massiliensis	Massiliimalia	Ruminococcaceae	Oscillospirales	Clostridia	Bacillota A	-0.017	0.020	4.1E-01	2897	80.6	-0.038	0.050	4.5E-01	561	79.7	-0.010	0.022	6.5E-01	2336	80.8	-0.026	0.016	1.1E-01	4338	75.4
QAKD01 sp003343965	QAKD01	Anaerovoracaceae	Peptostreptococcales	Clostridia	Bacillota A	-0.017	0.021	4.1E-01	2897	61.3	0.005	0.052	9.3E-01	561	61.0	-0.021	0.023	3.8E-01	2336	61.4	0.036	0.017	3.7E-02	4338	54.7
Acutalibacter sp900548545	Acutalibacter	Acutalibacteraceae	Oscillospirales	Clostridia	Bacillota A	0.016	0.020	4.2E-01	2897	76.4	-0.020	0.051	6.9E-01	561	75.6	0.024	0.022	2.9E-01	2336	76.6	0.023	0.016	1.6E-01	4338	75.4
Vescimonas sp900545585	Vescimonas	Oscillospiraceae	Oscillospirales	Clostridia	Bacillota A	-0.018	0.022	4.2E-01	2897	51.8	0.002	0.057	9.7E-01	561	51.2	-0.015	0.024	5.3E-01	2336	52.0	-0.034	0.018	5.9E-02	4338	49.0
Faecalibacillus faecis	Faecalibacillus	Coprobacillaceae	Erysipelotrichales	Bacilli	Bacillota	-0.017	0.022	4.2E-01	2897	51.4	-0.001	0.056	9.8E-01	561	52.0	-0.016	0.024	5.1E-01	2336	51.2	0.053	0.018	2.9E-03	4338	50.8
Ventrimonas sp003478505	Ventrimonas	Lachnospiraceae	Lachnospirales	Clostridia	Bacillota A	0.016	0.020	4.3E-01	2897	71.5	0.005	0.053	9.2E-01	561	71.1	0.010	0.023	6.7E-01	2336	71.6	0.057	0.016	4.4E-04	4338	71.4
Mediterraneibacter lactaris	Mediterraneibacter	Lachnospiraceae	Lachnospirales	Clostridia	Bacillota A	0.016	0.021	4.4E-01	2897	65.4	-0.025	0.053	6.4E-01	561	63.3	0.021	0.023	3.5E-01	2336	65.9	0.058	0.017	5.7E-04	4338	61.4
Luoshenia tenuis	Luoshenia	GCA-900066905	Christensenellales	Clostridia	Bacillota A	0.016	0.020	4.4E-01	2897	74.3	-0.015	0.052	7.7E-01	561	74.7	0.025	0.022	2.7E-01	2336	74.2	0.006	0.016	7.2E-01	4338	69.1
Muricomes contortus B	Muricomes	Lachnospiraceae	Lachnospirales	Clostridia	Bacillota A	0.015	0.022	4.8E-01	2897	52.8	-0.075	0.054	1.6E-01	561	54.5	0.034	0.024	1.6E-01	2336	52.4	0.049	0.017	5.5E-03	4338	52.2
Enterocloster sp900541315	Enterocloster	Lachnospiraceae	Lachnospirales	Clostridia	Bacillota A	-0.013	0.019	4.9E-01	2897	91.5	-0.097	0.049	4.8E-02	561	91.6	0.000	0.022	1.0E+00	2336	91.4	-0.022	0.015	1.6E-01	4338	93.1
Sutterella wadsworthensis A	Sutterella	Burkholderiaceae A	Burkholderiales	Gammaproteobacteria	Pseudomonadota	-0.017	0.024	4.9E-01	2897	31.1	-0.027	0.061	6.5E-01	561	29.9	-0.019	0.027	4.8E-01	2336	31.4	0.040	0.019	3.2E-02	4338	37.7
Scatomonas hejianensis	Scatomonas	Lachnospiraceae	Lachnospirales	Clostridia	Bacillota A	0.013	0.019	5.0E-01	2897	96.7	-0.083	0.049	9.1E-02	561	97.1	0.032	0.021	1.4E-01	2336	96.6	-0.004	0.015	7.7E-01	4338	95.5
Clostridium A leptum	Clostridium A	Acutalibacteraceae	Oscillospirales	Clostridia	Bacillota A	-0.012	0.019	5.2E-01	2897	97.3	-0.050	0.049	3.1E-01	561	97.1	-0.004	0.021	8.5E-01	2336	97.4	0.012	0.015	4.4E-01	4338	95.9
Anaerostipes hadrus	Anaerostipes	Lachnospiraceae	Lachnospirales	Clostridia	Bacillota A	-0.010	0.019	5.9E-01	2897	98.4	-0.089	0.048	6.3E-02	561	98.6	0.007	0.021	7.6E-01	2336	98.4	0.007	0.015	6.5E-01	4338	98.5
Copromonas faecavium	Copromonas	Lachnospiraceae	Lachnospirales	Clostridia	Bacillota A	-0.012	0.022	6.0E-01	2897	48.0	-0.051	0.057	3.7E-01	561	46.7	-0.002	0.025	9.2E-01	2336	48.3	0.037	0.018	4.5E-02	4338	41.7
Massilistercora sp902406105	Massilistercora	Lachnospiraceae	Lachnospirales	Clostridia	Bacillota A	0.011	0.023	6.3E-01	2897	44.9	0.021	0.058	7.2E-01	561	44.9	0.006	0.025	8.0E-01	2336	44.9	0.040	0.019	3.0E-02	4338	39.3
Alangreenwoodia sp905206455	Alangreenwoodia	Anaerovoracaceae	Peptostreptococcales	Clostridia	Bacillota A	-0.010	0.022	6.4E-01	2897	49.2	-0.106	0.055	5.7E-02	561	51.2	0.000	0.025	9.8E-01	2336	48.7	0.030	0.018	9.2E-02	4338	50.7
Lactonifactor sp009677585	Lactonifactor	Lachnospiraceae	Lachnospirales	Clostridia	Bacillota A	0.011	0.024	6.5E-01	2897	35.2	-0.023	0.058	6.9E-01	561	37.3	0.017	0.027	5.3E-01	2336	34.8	0.018	0.019	3.7E-01	4338	32.3
QAKW01 sp003343605	QAKW01	QAKW01	Oscillospirales	Clostridia	Bacillota A	-0.009	0.021	6.7E-01	2897	62.4	-0.032	0.053	5.5E-01	561	62.6	-0.002	0.023	9.2E-01	2336	62.4	0.074	0.017	2.1E-05	4338	55.6
Fimisoma avicola	Fimisoma	Anaerovoracaceae	Peptostreptococcales	Clostridia	Bacillota A	0.010	0.023	6.8E-01	2897	38.3	-0.044	0.058	4.5E-01	561	38.1	0.016	0.026	5.3E-01	2336	38.4	0.037	0.019	5.5E-02	4338	30.9
CAG-41 sp900066215	CAG-41	UBA1381	UBA1381	Clostridia	Bacillota A	0.007	0.019	7.0E-01	2897	98.1	-0.083	0.047	7.7E-02	561	98.2	0.021	0.021	3.2E-01	2336	98.1	0.054	0.015	3.3E-04	4338	97.5
Eubacterium G sp000432355	Eubacterium G	Lachnospiraceae	Lachnospirales	Clostridia	Bacillota A	-0.008	0.021	7.1E-01	2897	61.7	-0.017	0.052	7.4E-01	561	63.8	0.003	0.023	9.1E-01	2336	61.2	-0.033	0.017	5.4E-02	4338	56.6
HGM12545 sp900761925	HGM12545	Lachnospiraceae	Lachnospirales	Clostridia	Bacillota A	0.008	0.021	7.2E-01	2897	56.9	0.037	0.056	5.1E-01	561	55.1	-0.002	0.024	9.5E-01	2336	57.4	0.020	0.018	2.6E-01	4338	46.0
Solibaculum mannosilyticum	Solibaculum	Acutalibacteraceae	Oscillospirales	Clostridia	Bacillota A	-0.006	0.020	7.5E-01	2897	80.7	0.018	0.050	7.1E-01	561	76.8	-0.020	0.022	3.7E-01	2336	81.6	0.038	0.016	1.8E-02	4338	77.8
Massilioclostridium coli	Massilioclostridium	Ruminococcaceae	Oscillospirales	Clostridia	Bacillota A	-0.006	0.019	7.6E-01	2897	87.6	0.008	0.050	8.7E-01	561	84.8	-0.008	0.022	7.2E-01	2336	88.3	0.025	0.015	1.1E-01	4338	89.3
Holdemania filiformis	Holdemania	Erysipelotrichaceae	Erysipelotrichales	Bacilli	Bacillota	-0.006	0.020	7.7E-01	2897	68.9	-0.042	0.051	4.1E-01	561	69.0	0.002	0.023	9.4E-01	2336	68.9	-0.034	0.016	4.0E-02	4338	68.6
Evtepia gabavorous	Evtepia	Oscillospiraceae	Oscillospirales	Clostridia	Bacillota A	-0.006	0.022	7.7E-01	2897	52.9	-0.088	0.055	1.1E-01	561	52.8	0.011	0.024	6.4E-01	2336	53.0	-0.003	0.017	8.7E-01	4338	52.5
Mediterraneibacter massiliensis	Mediterraneibacter	Lachnospiraceae	Lachnospirales	Clostridia	Bacillota A	-0.006	0.022	7.7E-01	2897	47.2	-0.019	0.058	7.4E-01	561	44.7	-0.006	0.025	8.0E-01	2336	47.7	0.014	0.018	4.4E-01	4338	48.2
Flemingibacterium sp900546315	Flemingibacterium	CAG-272	Oscillospirales	Clostridia	Bacillota A	0.006	0.020	7.8E-01	2897	72.7	0.035	0.052	5.0E-01	561	72.4	0.001	0.023	9.5E-01	2336	72.7	-0.001	0.017	9.4E-01	4338	67.5
Pullichristensenella excrementipullorum	Pullichristensenella	CAG-74	Christensenellales	Clostridia	Bacillota A	0.006	0.021	7.9E-01	2897	67.9	-0.035	0.052	5.0E-01	561	67.4	0.015	0.023	5.1E-01	2336	68.0	0.014	0.017	4.1E-01	4338	60.8
Anaeromassilibacillus stercoravium	Anaeromassilibacillus	Acutalibacteraceae	Oscillospirales	Clostridia	Bacillota A	-0.005	0.020	7.9E-01	2897	72.5	0.014	0.050	7.9E-01	561	77.0	-0.004	0.023	8.6E-01	2336	71.4	0.025	0.016	1.3E-01	4338	71.5
Eubacterium G sp000435815	Eubacterium G	Lachnospiraceae	Lachnospirales	Clostridia	Bacillota A	0.006	0.024	8.0E-01	2897	32.7	-0.025	0.062	6.8E-01	561	32.3	0.011	0.027	6.9E-01	2336	32.8	-0.005	0.019	8.0E-01	4338	36.5
UMGS1696 sp900753285	UMGS1696	CAG-272	Oscillospirales	Clostridia	Bacillota A	0.006	0.022	8.0E-01	2897	53.2	0.037	0.056	5.0E-01	561	54.9	0.004	0.024	8.6E-01	2336	52.8	-0.002	0.018	9.0E-01	4338	46.0
Eubacterium F sp900539115	Eubacterium F	Lachnospiraceae	Lachnospirales	Clostridia	Bacillota A	-0.004	0.022	8.5E-01	2897	53.3	-0.026	0.055	6.3E-01	561	53.5	0.004	0.024	8.6E-01	2336	53.3	0.026	0.018	1.5E-01	4338	47.2
Anaeromassilibacillus sp001305115	Anaeromassilibacillus	Acutalibacteraceae	Oscillospirales	Clostridia	Bacillota A	-0.003	0.024	9.0E-01	2897	33.5	0.003	0.065	9.6E-01	561	27.6	-0.006	0.026	8.2E-01	2336	34.9	0.042	0.019	3.0E-02	4338	33.0
ER4 sp000765235	ER4	Oscillospiraceae	Oscillospirales	Clostridia	Bacillota A	-0.002	0.019	9.1E-01	2897	85.5	0.016	0.050	7.5E-01	561	85.9	-0.014	0.022	5.3E-01	2336	85.4	0.002	0.016	8.8E-01	4338	85.0
Massilioclostridium methylpentosum	Massilioclostridium	Ruminococcaceae	Oscillospirales	Clostridia	Bacillota A	-0.002	0.023	9.1E-01	2897	40.3	-0.109	0.059	6.5E-02	561	41.9	0.016	0.026	5.3E-01	2336	39.9	0.027	0.019	1.5E-01	4338	34.7
Ruthenibacterium sp018380115	Ruthenibacterium	Ruminococcaceae	Oscillospirales	Clostridia	Bacillota A	0.002	0.021	9.2E-01	2897	65.0	-0.038	0.053	4.8E-01	561	65.2	0.012	0.023	6.1E-01	2336	65.0	0.047	0.017	5.3E-03	4338	63.9
Clostridium Q fessum	Clostridium Q	Lachnospiraceae	Lachnospirales	Clostridia	Bacillota A	-0.001	0.019	9.4E-01	2897	90.4	0.033	0.050	5.1E-01	561	90.4	-0.009	0.021	6.7E-01	2336	90.5	0.055	0.015	3.6E-04	4338	92.0
Paraprevotella clara	Paraprevotella	Bacteroidaceae	Bacteroidales	Bacteroidia	Bacteroidota	0.001	0.023	9.8E-01	2897	37.6	0.046	0.060	4.4E-01	561	36.4	-0.010	0.026	7.0E-01	2336	37.9	0.054	0.018	2.5E-03	4338	48.8
QAMH01 sp900544245	QAMH01	QAMH01	Coriobacteriales	Coriobacteriia	Actinomycetota	0.000	0.022	9.9E-01	2897	54.1	0.019	0.056	7.3E-01	561	53.7	0.005	0.025	8.5E-01	2336	54.2	0.061	0.018	5.6E-04	4338	54.6

Table S4. Pearsons's correlation between gut microbial species highly associated with microbial genes for 5α-reductase type 1

	Odoribacter splanchnicus *Bacteroides uniformis*		*Odoribacter splanchnicus* *Parabacteroides distasonis*		*Bacteroides uniformis* *Parabacteroides distasonis*	
	**Pearsons's r**	**P**	**Pearsons's r**	**P**	**Pearsons's r**	**P**
**All**	0.35	2.5E-201	0.43	<2.2E-308	0.37	3.3E-228
**Women**	0.31	1.7E-66	0.43	3.9E-129	0.31	1.2E-65
**Pre**	0.29	5.0E-12	0.41	2.2E-24	0.29	4.4E-12
**Post**	0.32	3.5E-56	0.43	1.8E-106	0.32	2.6E-55
**Men**	0.37	6.3E-137	0.44	4.2E-200	0.40	2.5E-169

Pearsons's correlation using inverse rank transformed data.

Table S5. Sensitivity analyses in women excluding women with recent use of antibiotics

Exposure	Outcome	Models adjusting for antibiotic use			Models excluding antibiotic users		
		β (95% CI)	P	n	β (95% CI)	P	n
Odoribacter splanchnicus	GM 5α-reductase type 1	0.54 (0.50-0.57)	9.4E-200	2897	0.55 (0.51-0.58)	8.6E-197	2737
Bacteroides uniformis	GM 5α-reductase type 1	0.50 (0.47-0.53)	7.1E-177	2897	0.50 (0.47-0.53)	9.5E-166	2737
Parabacteroides distasonis	GM 5α-reductase type 1	0.47 (0.44-0.50)	1.3E-149	2897	0.47 (0.44-0.51)	1.9E-141	2737
Odoribacter splanchnicus	Circulating DHT/T ratio	0.087 (0.019-0.127)	3.0E-06	2897	0.083 (0.046-0.120)	1.4E-05	2737
Parabacteroides distasonis	Circulating DHT/T ratio	0.075 (0.018-0.115)	5.4E-05	2897	0.061 (0.024-0.099)	1.3E-03	2737
GM 5α-reductase type 1	Circulating DHT/T ratio	0.066 (0.03-0.102)	3.2E-04	2897	0.061 (0.024-0.098)	1.2E-03	2737

Linear regressions for the relative abundance of microbial genes for 5α-reductase type 1 as outcome and relative abundance of bacterial species as exposures; circulating DHT/T ratio as outcome and relative abundance of bacterial species as exposure; and circulating DHT/T ratio as outcome and relative abundance of microbial genes for 5α-reductase type 1 as exposure with adjustment for antibiotic use or exclusion of antibiotic-treated women. All models were adjusted for age, extraction plate, and menopausal status. Exposures and outcomes are inverse rank transformed. β are expressed as standard deviation change in outcome per standard deviation of the exposure. CI, confidence interval
